# Prognostic imaging biomarkers for diabetic kidney disease (iBEAt): study protocol

**DOI:** 10.1186/s12882-020-01901-x

**Published:** 2020-06-29

**Authors:** Kim M. Gooding, Chrysta Lienczewski, Massimo Papale, Niina Koivuviita, Marlena Maziarz, Anna-Maria Dutius Andersson, Kanishka Sharma, Paola Pontrelli, Alberto Garcia Hernandez, Julie Bailey, Kay Tobin, Virva Saunavaara, Anna Zetterqvist, David Shelley, Irvin Teh, Claire Ball, Sapna Puppala, Mark Ibberson, Anil Karihaloo, Kaj Metsärinne, Rosamonde E. Banks, Peter S. Gilmour, Michael Mansfield, Mark Gilchrist, Dick de Zeeuw, Hiddo J. L. Heerspink, Pirjo Nuutila, Matthias Kretzler, Matthew Welberry Smith, Loreto Gesualdo, Dennis Andress, Nicolas Grenier, Angela C. Shore, Maria F. Gomez, Steven Sourbron, for the BEAt-DKD consortium

**Affiliations:** 1 Diabetes and Vascular Medicine, University of Exeter Medical School, Barrack Road, Exeter, EX2 5AX UK; 2 NIHR Exeter Clinical Research Facility, Royal Devon and Exeter NHS Foundation Trust, Exeter, UK; 3 Department of Nephrology, University of Michigan, Ann Arbor, USA; 4 Department of Emergency and Organ Transplantation, Nephrology Unit, University of Bari Aldo Moro, Bari, Italy; 5 Department of Medicine, Division of Nephrology, Turku University Hospital, Turku, Finland; 6 Turku PET Centre, University of Turku, Turku, Finland; 7 Department of Clinical Sciences in Malmö, Lund University Diabetes Centre, Lund University, Malmo, Sweden; 8 Department of Imaging, Infection, Immunity and Cardiovascular Disease, University of Sheffield, Sheffield, UK; 9 Astellas Pharma Europe B.V, Meppel, The Netherlands; 10 Leeds Teaching Hospitals NHS Trust, Leeds, UK; 11 Department of Renal Medicine and Renal Transplantation, Leeds Teaching Hospitals NHS Trust, Leeds, UK; 12 Department of Medical Physics, Division of Medical Imaging, Turku University Hospital, Turku, Finland; 13 Advanced Imaging Centre, University of Leeds, Leeds, UK; 14 Leeds Institute of Cardiovascular and Metabolic Medicine, University of Leeds, Leeds, UK; 15 Swiss Institute of Bioinformatics, Lausanne, Switzerland; 16 Novo Nordisk Research Center Seattle, Inc., Seattle, USA; 17 Leeds Institute of Medical Research at St James’s, University of Leeds, Leeds, UK; 18 The Drug Development Team, Leiden, The Netherlands; 19 Department of Clinical Pharmacy and Pharmacology, University Medical Center Groningen, Groningen, The Netherlands; 20 University of Groningen, Groningen, The Netherlands; 21 Computational Medicine and Bioinformatics, University of Michigan, Ann Arbour, USA; 22 AbbVie, Scottsdale, USA; 23 Service de Radiologie, CHU de Bordeaux, Université de Bordeaux, Bordeaux, France

**Keywords:** Diabetic kidney disease, Type 2 diabetes, Magnetic resonance imaging, Ultrasound, Albuminuria, Chronic kidney disease stages 1–3, Prospective cohort, Renal decline, Biomarkers, Progression

## Abstract

**Background:**

Diabetic kidney disease (DKD) remains one of the leading causes of premature death in diabetes. DKD is classified on albuminuria and reduced kidney function (estimated glomerular filtration rate (eGFR)) but these have modest value for predicting future renal status. There is an unmet need for biomarkers that can be used in clinical settings which also improve prediction of renal decline on top of routinely available data, particularly in the early stages. The iBEAt study of the BEAt-DKD project aims to determine whether renal imaging biomarkers (magnetic resonance imaging (MRI) and ultrasound (US)) provide insight into the pathogenesis and heterogeneity of DKD (primary aim) and whether they have potential as prognostic biomarkers in DKD (secondary aim).

**Methods:**

iBEAt is a prospective multi-centre observational cohort study recruiting 500 patients with type 2 diabetes (T2D) and eGFR ≥30 ml/min/1.73m^2^. At baseline, blood and urine will be collected, clinical examinations will be performed, and medical history will be obtained. These assessments will be repeated annually for 3 years. At baseline each participant will also undergo quantitative renal MRI and US with central processing of MRI images. Biological samples will be stored in a central laboratory for biomarker and validation studies, and data in a central data depository. Data analysis will explore the potential associations between imaging biomarkers and renal function, and whether the imaging biomarkers improve the prediction of DKD progression. Ancillary substudies will: (1) validate imaging biomarkers against renal histopathology; (2) validate MRI based renal blood flow measurements against H_2_O^15^ positron-emission tomography (PET); (3) validate methods for (semi-)automated processing of renal MRI; (4) examine longitudinal changes in imaging biomarkers; (5) examine whether glycocalyx and microvascular measures are associated with imaging biomarkers and eGFR decline; (6) explore whether the findings in T2D can be extrapolated to type 1 diabetes.

**Discussion:**

iBEAt is the largest DKD imaging study to date and will provide valuable insights into the progression and heterogeneity of DKD. The results may contribute to a more personalised approach to DKD management in patients with T2D.

**Trial registration:**

Clinicaltrials.gov (NCT03716401).

## Background

### The BEAt-DKD project

Diabetic kidney disease (DKD) is the leading cause of end stage renal disease [1, 2]. It is currently estimated that approximately 20–40% of people with diabetes will develop DKD [3], and this is expected to rise in the future. With the global increase in the prevalence of diabetes [4], particularly type 2 diabetes (T2D), DKD is reaching epidemic proportions, with health and quality of life implications (e.g. increased risk of cardiovascular mortality) for the individual [5]. Even with current approaches for the management of diabetes and renin-angiotensin-aldosterone system blockade, there is still a large residual risk in DKD [6].

DKD is routinely classified clinically based on albuminuria and reduced kidney function (estimated glomerular filtration rate (eGFR)). Albuminuria is traditionally viewed as a hallmark of diabetes related kidney damage. However, there are limitations of using albuminuria to classify DKD, which include the need for multiple measurements to mitigate spurious results due to factors such as infection and physical activity. Additionally, the heterogeneity of DKD is increasingly recognised, as reflected, for example, by the disparity in DKD progression (fast versus slow DKD progression) and by patients with declining kidney function but normoalbuminuria. For example, 51% of participants in the UK Prospective Diabetes Study whose eGFR declined below 60 ml/min/1.73 m^2^ had normoalbuminuria [7]. This heterogeneity in DKD highlights the need for novel biomarkers and a more personalised medicine-based approach to managing DKD.

The fundamental aim of the Biomarker Enterprise to Attack DKD (BEAt-DKD) consortium is to increase our understanding of the pathogenesis and heterogeneity of DKD, enabling the identification of novel biomarkers and treatment targets, to facilitate a more personalised medicine-based approach to managing DKD and increase the efficiency of clinical trials [8].

### Imaging biomarkers for DKD

Cross-sectional imaging, in particular MRI and US, is increasingly proposed as an alternative source of biomarkers to inform chronic kidney disease (CKD) management [9, 10]. An important example is the qualification by the Food and Drug Administration (FDA) and the European Medicines Agency (EMA) of Total Kidney Volume (TKV) as a prognostic enrichment biomarker for Autosomal Dominant Polycystic Kidney Disease (ADPKD) – one of only a handful of clinical biomarkers approved by the FDA so far [11, 12]. In recent years the interest is increasingly moving towards advanced MRI and US techniques that are sensitive to structural and functional tissue characteristics such as perfusion, oxygenation, blood flow, glomerular filtration, tubular flow, fibrosis, inflammation, metabolism and tissue composition. Additional utility derives from the fact that these characteristics can be measured separately for left and right kidney and for cortex and medulla, and that they can characterise functional and structural heterogeneity within those areas.

A number of preclinical and single-centre clinical studies have indicated a potential utility of MRI and US biomarkers in DKD specifically. For instance, US-based measurements of kidney volume have suggested that kidney enlargement is associated with poorer outcomes in early and advanced DKD, despite the often better GFR of larger kidneys [13–15]. A possible explanation is that hypertrophy indicates a sustained state of primary or secondary hyperfiltration and associated damage due to intraglomerular pressures. A mechanistic study suggested that the MRI method BOLD (Blood Oxygenation Level Dependent MRI) can highlight areas at risk of ischemic damage due to oxygen depletion after sustained hyperfiltration [16], and recent clinical studies have confirmed that the BOLD signal is predictive of CKD progression [17, 18]. Some biomarkers derived from diffusion-weighted MRI are sensitive to renal fibrosis [19, 20], can identify microstructural changes after sustained hyperfiltration [21], and can potentially detect disease progression earlier than eGFR [22]. Kidney perfusion can also be measured with MRI and has shown a correlation with eGFR in DKD [23]. Other non-renal imaging biomarkers characterising general risk factors for diabetes and its associated complications may be relevant in this context as well and can easily be measured in the same MRI scan session, such as liver and pancreatic fat fraction [24].

### iBEAt study aims and objectives

The aim of iBEAt is to evaluate the utility of imaging biomarkers in DKD in a large cohort of heterogeneous T2D patients, in the early stages of DKD where there is high potential for effective interventions to slow the rate of DKD progression.

The key hypotheses are that (1) imaging biomarkers of DKD provide additional information on the pathogenesis and histological and clinical heterogeneity of DKD compared to biomarkers sourced from blood or urine samples or physical exams, and that (2) changes in imaging biomarkers precede increases in albuminuria and decline in kidney function as measured by eGFR slope. As a result, we expect imaging biomarkers to improve the identification of DKD patients at risk of rapid decline in kidney function, either when used alone or combined with clinical data or biological fluid biomarkers.

An additional aim of the iBEAt study is to establish a biobank of biological samples (blood- and urine-based) from well-characterised patients not only for use within the BEAt-DKD programme but also for future DKD collaborative studies with scientists outside BEAt-DKD. This will facilitate biomarker discovery studies using novel blood- and urine-based biomarkers and may serve as the foundation for a comprehensive multi-scale phenotyping strategy linking data from blood, urine, tissue, microvascular assessments, imaging, physical measurements and medical histories.

The specific study objectives are:

- Primary objective: To examine whether renal imaging biomarkers are associated with severity of DKD as defined using classical biomarkers of DKD, albuminuria and eGFR, in individuals with T2D and eGFR ≥ 30 ml/min/1.73m^2^.
- Secondary objective: To examine whether renal imaging biomarkers at baseline are associated with changes in renal function over time as measured by eGFR slope over a subsequent 3-year period.

### Overview of iBEAt study design and organisation

iBEAt is a prospective observational study that will enrol 500 participants with T2D and eGFR greater than 30 mL/min/1.73m^2^ across multiple European centres.

A schematic overview of the study assessments is presented in Table 1. At baseline, medical histories will be collected for each participant and they will undergo comprehensive renal imaging (MRI and US), biological sample collection (blood and urine) and physical measurements. They will then be invited back annually for 3 years, where all measurements except the imaging will be repeated.

**Table 1:** Overview of the study showing type of data collected (rows) for each time point (columns)

*Protocol Details*	*Screening*	*Baseline*	*Year 1*	*Year 2*	*Year 3*
***Time Window***	***Day 0***	***0-3 m***	***1y ± 3 m***	***2y ± 3 m***	***3y ±*3*m***
*Informed Consent*	*X*				
*Demographics*	*X*				
*Clinical Information*		*X*	*X*	*X*	*X*
*Local lab value collection*	*X*	*X*	*X*	*X*	*X*
*Blood Collection (including DNA and RNA)*		*X*	*X*	*X*	*X*
*Urine Collection (random)*	*X*				
*Urine Collection (1st morning & additional void)*		*X*	*X*	*X*	*X*
*MRI*		*X*			
*US*		*X*			

*y* year, *m* month

**Fig. 1:**
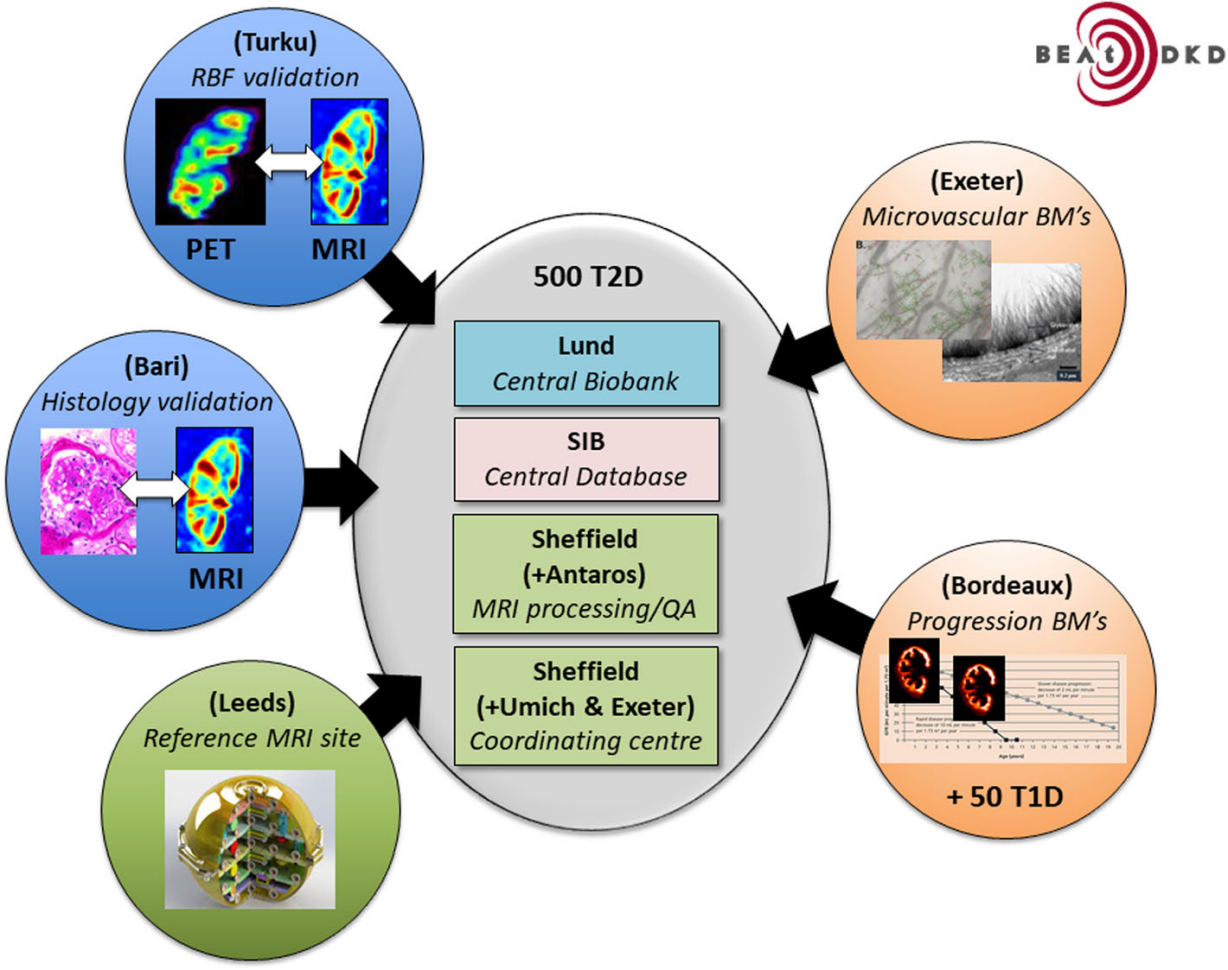
Overview of study organisation. iBEAt study organigram showing central roles (grey ellipse) and recruiting sites with ancillary studies (circles). BMs = biomarkers; QA = quality assurance; RBF = renal blood flow; PET = Positron-emission tomography; SIB = Swiss Institute of Bioinformatics; T1D = type 1 diabetes; Umich = University of Michigan

The organisation of iBEAt is shown in Fig. 1. The study is led by the coordinating centre in Sheffield with a co-lead in Exeter and a study manager in Michigan. Currently there are 5 recruiting centres (University of Leeds, University of Exeter Medical School, University of Bari, University of Bordeaux and University of Turku), a central laboratory (Lund University) and a central data repository (Swiss Institute of Bioinformatics (SIB)). University of Sheffield is the central imaging processing and quality assurance (QA) site, with support from Antaros Medical (Sweden) for biomarkers of body composition. All ethical and relevant local approvals are in place at each recruiting site. As a BEAt-DKD work package the study is supported by the BEAt-DKD consortium Steering Committee and an external Scientific Advisory Board.

## Methods

### iBEAt participants

The iBEAt study will recruit participants with a diagnosis of T2D, eGFR greater or equal to 30 mL/min/1.73m^2^, aged between 18 and 80 years, who are able to give informed consent, and do not satisfy any of the exclusion criteria. The exclusion criteria are listed in Table 2 (see also additional file 3.0).

**Table 2:** List of iBEAt exclusion criteria (see also additional file 3.0)

*Permanent dialysis*	*Current metastatic malignancy*
*Renal transplantation*	*Current malignancy with expected survival < study follow up period (4 years)*
*Known clinical history of urinary obstruction on renal US*	*Melanomatous skin cancer < 5 years ago (fully resected melanoma > 5 years ago,* i.e. *surgical cure, can be recruited)*
*Post-voiding residue over 100 ml, or pyelectasis*	*Use of investigational drug within 1 month prior to screening*
*Known clinical history of aortic endoprothesis at the renal level*	*Current pregnancy*
*Known current or clinical history of renal or urinary tract malignancy*	*Use of antiretroviral medication*
*Biopsy proven non-diabetic primary renal disease*	*History of Hepatitis B or Hepatitis C*
*Autosomal dominant polycystic kidney disease (APKD)*	*Significant comorbidities with life expectancy of < 1 year*
*Cirrhotic liver disease or non-cirrhotic chronic liver disease where alanine aminotransferase > 2 x upper limit of normal*	*Standard contrast enhanced-MRI exclusions (*e.g. *cochlear implant, aneurysm clips, claustrophobia and known allergy to Gadolinium contrast)*
*Renal stones causing CKD*	*Any other significant disease or disorder which, in the opinion of the investigators, may either put the patient at risk because of participation in the study, or may influence the result of the study, or the patient’s ability to participate in the study*

iBEAt will recruit across the A1-A3 albuminuria range (normo-, micro- and macroalbuminuria) and the G1-G3 eGFR range (G1: ≥90; G2: 60–89; G3: 30–59 ml/min/1.73m^2^). In line with the National Kidney Foundation guidelines [25], albuminuria will be classified using two independent values of ACRs measured within a 3-month period, and a third if the classification differs between the first two samples. We define normo-, micro- and macro-albuminuria as an ACR of < 2.5, 2.5–25, > 25 mg/mmol for men, respectively, and as ACR < 3.5, 3.5–35, > 35 mg/mmol for women, respectively.

Participants are enrolled in the study following provision of written informed consent. Eligibility criteria can be checked via medical records or by performing additional assessments if no data available (e.g. eGFR). If no ACR measurement is available from the previous 3 months a random urine sample for ACR analysis will be collected following consent.

### Baseline study assessments

#### Participant preparation

Participants are required to be on stable diabetes and hypertension related treatment (though dose changes to current medications are allowed) for the 3 months prior to study assessments. Study assessments will be performed in the morning following an overnight fast (> 8 h). Medications may be withheld or altered on the day of the study visit to ensure participant wellbeing (e.g. omitting morning insulin injection to maintain blood glucose levels) and integrity of the study. The study visit will include a checklist to record the adherence to instructions (additional file 3.1). A point of care glucose measurement will be performed upon participant arrival and the visit will be cancelled if glucose levels are below 3.5 mmol/L or if the participant reports a symptomatic hypoglycaemic event on the morning of the visit. If the visit goes ahead, blood samples will be taken first. All other assessments are performed following a standardised meal of 2 slices brown bread, butter and a glass of water (250 mL).

#### MRI biomarkers

Fifty nine primary MRI biomarkers will be recorded (see additional file 1.1 for a full list), characterising general body composition (e.g. visceral fat volume, pancreatic and liver fat fraction), renal morphology (e.g. parenchymal volume, cortical thickness), renal tissue structure (e.g. MR relaxation times, apparent water diffusion coefficient), renal hemodynamics (e.g. cortical perfusion, renal artery blood flow), filtration (e.g. single-kidney GFR, filtration fraction). All MR scanning is performed at 3 T on Siemens, Philips and General Electric scanners. MRI data are uploaded on a central XNAT database hosted by SIB and quality controlled within 48 h by the central processing site.

The MRI protocol takes approximately 1 h and 10mins and involves the injection of a quarter dose of clinical macrocyclic MRI contrast agent. The protocol was first developed on the reference Siemens scanner in Leeds using an iterative optimisation guided by the NIST (National Institute of Standards and Technology) phantom and healthy volunteers. The resulting final protocol was then characterised on each MRI vendor using a repeatability study in healthy volunteers to determine within-site variability (5 volunteers with 4 scans each). The NIST phantom is scanned at regular intervals in all sites to check for between-site calibration. Full details of the MRI acquisition protocol on the 3 T Siemens reference scanner in Leeds can be found in additional file 1.2.

#### Renal ultrasound

Kidney size will be non-invasively determined from longitudinal and transversal images of each kidney. Resistive index (RI), indicator of the resistance to flow within the kidney, will be determined from three measurements in each kidney (upper, mid and lower poles). The mean of the three measurements will represent RI for each respective kidney. A list of US biomarkers is provided in the additional file 3.7 and the Standard Operating Procedures (SOPs) for US scanning are in additional file 1.3.

#### Blood and urine sampling

Fasting blood samples (~ 70 ml designated for iBEAt central requirement) will be collected from each participant for participant characterisation and biomarker analysis. Glycated haemoglobin (HbA1c), full blood count and fasting glucose assessments will be performed locally (additional file 3.4). The remaining plasma and serum samples will be processed and stored following a standardised protocol (see additional files 2.1-2.3). A first morning urine void and one additional morning void (same day) are collected by all participants. A small proportion of the first morning void is sent to the local laboratories for ACR assessment. The remainder of the first morning and second void are then processed and stored following a standardised protocol (see additional files 2.1-2.3).

The standardised sample collection and processing protocol, informed by PROVALID and NEPTUNE trials [26, 27], was developed to maximise the utility of stored samples for future biomarker analysis (e.g. lipidomics, RNA analysis, urinary vesicles and urinary sediment) within BEAt-DKD and to form a biobank for future DKD related studies. A separate check is performed to confirm that all samples are collected and processed according to protocol (additional file 3.8).

The central laboratory is located at the Clinical Research Centre (CRC) facility in Malmö (University of Lund). The central lab will prepare and distribute kits with sample collection and processing materials for each patient labelled and barcoded with their unique study ID. Each kit comprises of 66 storage tubes per patient. The samples will be temporarily stored at each recruiting site, with regular shipments returning them to the biobank in Malmö. Samples will be stored under secure conditions and monitored with a dedicated electronic sample tracking system (Laboratory Information Management Systems). A small volume of blood and urine will be analysed at the central laboratory in Malmö for known clinical biomarkers according to standardised methods, e.g. renal function (serum creatinine, cystatin C, potassium and albumin), lipid profile (total cholesterol and sub-fractions, triglycerides) and c-reactive protein. The remainder will be stored for future analyses by BEAt-DKD investigators. The samples will also remain available for secondary research provided approval is granted by the iBEAt steering committee.

#### Physical examination

The core physical examination assessments include blood pressures (sitting and standing blood pressures) and anthropometrics (height, weight, waist and hip circumference). See additional file 3.2 for details.

#### Medical history

A detailed medical history (including, for example, current medications, smoking history and presence of co-morbidities) is also collected. See additional files 3.3 and 3.6 for data fields that are captured.

#### Routine laboratory data

Routine local laboratory data will be captured from medical records to aid in the interpretation of the results by tracking temporal changes at a finer time scale than the yearly follow-ups. Only laboratory values available for clinical indication will be captured at this time. Additional file 3.5 lists the data fields to be captured but missing data from the local chart is not deemed a protocol violation.

### Follow-up study assessments

The blood and urine collection, medical history and physical examination will be repeated at 1, 2 and 3 years (± 3 months) following study enrolment. For participants who are unable to attend the local research centre for an annual follow-up visit but are still willing to participate in the study, an update on their medical history will be collected via direct communication with the participant and / or by accessing their available medical records.

### Data management

Clinical images, associated data and metadata will be stored using the XNAT platform (www.xnat.org) hosted on the dedicated BEAt-DKD server at SIB. Clinical study data will be managed using REDCap (www.project-redcap.org), also installed on the dedicated BEAt-DKD server. All variables will be recorded on iBEAt central case report form (CRFs – see additional files 3.0-3.8) and uploaded onto the central RedCap instance. It is envisaged that the iBEAt clinical study will be set up as a federated node enabling remote analysis of the data generated in the future, and integration of the iBEAt data with other datasets collected in BEAt-DKD.

### Statistical considerations

#### Sample size

There are insufficient a priori data available to perform a reliable formal power calculation for this study. Thus, a more pragmatic approach was adopted, with the sample size taking into account the feasibility aspects of the study, for example, the number of imaging facilities and the estimated rate of recruitment in each centre. This produced a target population of 500 patients, which will result in the largest quantitative imaging DKD study, to date, both in terms of patient numbers and regarding patient follow-up. Participants will be recruited across various stages of DKD, as measured by ACR (A1, A2, A3) and eGFR (G1 + G2, G3). This will produce a heterogeneous population enabling the evaluation of the associations between imaging biomarkers and DKD in T2D patients.

#### Statistical analysis plan

Descriptive statistics of all covariates across the study centres will assess the bivariate relationships between sets of related covariates. A cross-sectional analysis will be performed using data collected at baseline, as well as a longitudinal analysis using the imaging data collected at baseline and blood and urine markers collected annually. Given the large number of covariates in the study, a variable selection and regularisation method such as LASSO [28] or the elastic net regularisation [29] will be used. Modelling will use linear models for the cross-sectional analyses, and linear mixed effects models for the longitudinal analysis. The analyses will be performed separately in each ACR / eGFR stratum, and the modelling of the whole cohort will account, whenever possible, for differences in potential associations across the strata. We will adjust for multiple comparisons as needed. If any renal imaging biomarkers are identified as promising early markers of DKD progression, they will be used in risk prediction. Their predictive accuracy as measured by prediction error, receiver operating characteristics curve (ROC) and the area under the ROC (area under the curve, AUC) will be evaluated using cross-validation. A conservative approach will be adopted in all analyses, for example, limiting the number of models fitted and statistical tests performed. All analyses will be treated as exploratory and hypotheses generating, rather than hypothesis testing.

### Ancillary studies

Building on the strengths and interests across the iBEAt participating centres, six ancillary studies have been incorporated within the central iBEAt study. Participants taking part in the ancillary studies will be recruited from the central iBEAt study at the relevant sites.

#### Ancillary study 1

To examine whether MRI and US based imaging biomarkers correlate with histopathological markers of DKD and discriminate different renal lesions in this T2D cohort. For this ancillary study, led by Bari University, iBEAt participants will undergo a renal tissue core biopsy. All biopsies will be digitalised and characterised by light microscopy (hematoxylin-eosin, periodic acid-Schiff, silver methenamine, and Masson’s trichrome), immunofluorescence microscopy (with the use of antisera against IgG, IgM, IgA, C3, C4, C1q and fibrinogen) and electron microscopy. Glomerular and vascular lesions, interstitial cell infiltrate, fibrosis and tubular atrophy will be quantified to classify patients accordingly [30]. Samples will also be processed and stored for later biomarker discovery. The procedures for processing, storing and capturing meta-data regarding the renal biopsy tissue are described in more detail in additional file 4.0.

#### Ancillary study 2

To examine whether MRI-based measurements of renal blood flow correlate with H_2_O^15^-positron emission tomography (PET), considered to be a reference measurement. For this ancillary study, led by Turku University, a direct comparison of MRI and PET-based measurements of renal blood flow will be performed in a cohort of iBEAt participants. Renal perfusion will be assessed during hyperaemia with both systems.

#### Ancillary study 3

To validate automated or semi-automated processing of multiparametric renal MRI. In its current form the generation of biomarkers from complex functional MRI scans involves significant manual intervention as well as automated but slow iterative optimisation methods. In this study, led by Sheffield University, a subset of the iBEAt data will be used as training data to develop an ideally automated approach for image processing, which will then be validated on the remaining test data against the manual results.

#### Ancillary study 4

To investigate the longitudinal changes in MRI and US based biomarkers, compare them against changes in eGFR and other known markers, and determine whether changes in imaging biomarkers precede DKD progression as assessed by eGFR decline. For this study, performed by the Universities of Bordeaux and Exeter, a cohort of 100 patients will receive repeat MRI and US after 2 years, and changes in imaging biomarkers over that period will be correlated against changes in eGFR and other assessments.

#### Ancillary study 5

To examine whether the glycocalyx, microvascular function and structure (retinal and skin) are (1) altered in microalbuminuria (2); associated with DKD progression as assessed by eGFR decline and (3) associated with novel MRI and US imaging DKD biomarkers. For this study, led by Exeter University, iBEAt participants will also undergo comprehensive microvascular assessments (including non-invasive estimation of sublingual endothelial glycocalyx integrity, retinal vascular oxygenation and skin maximum hyperaemia) at baseline and at 2 years follow-up.

#### Ancillary study 6

A pilot study to examine whether the findings in T2D can be extrapolated to Type 1 diabetes. In this ancillary study, led by Bordeaux University, a cohort of 50 patients with Type 1 diabetes for 15 years or more will be assessed using the same procedures as the Type 2 cohort and observed findings / trends will be compared across the two populations.

### Patient and public involvement

Patient and public involvement and engagement is a significant component of the iBEAt study. Potential participants have played an important role in iBEAt, reviewing the protocol to ensure the feasibility of the study design (core and ancillary studies) as well as contributing to the development of patient facing documents (e.g. patient information sheets), ensuring that they are clear and informative. Participants within the iBEAt study will play an integral role in the dissemination of the study results to the wider, non-expert population. Within the BEAt-DKD consortium discussions with patient representatives, ranging from experienced patient advocates to iBEAt participants, will help inform how research from the BEAt-DKD consortium is taken forward to implement a more precision medicine based approach in DKD into clinical practice; for example, validation and qualification of new biomarkers by regulatory agencies, optimising clinical study design and integration in the regulatory process of drug registration. Indeed, this has already commenced with an iBEAt participant and other patient representatives attending the 2nd BEAt-DKD Stakeholders’ symposiums in April 2019 [31].

## Discussion

Quantitative and functional imaging of the kidney has been an active topic of research in the MRI physics and radiology community for over two decades [32], but the last few years have seen an explosive growth in clinical interest. The first international meeting on functional renal MRI was held in 2015 and attendance has been increasing steadily in subsequent meetings [33–35]. In 2017, a pan-European network of researchers in renal MRI (www.renalmri.org) was funded for 4 years by the European Cooperation in Science and Technology (www.cost.eu). In 2018, Nephrology Dialysis Transplantation published a special issue on renal MRI with a clinical position statement supported by over 30 authors including leading European nephrologists [9]. In the same year, in the US, the National Institute of Diabetes and Digestive and Kidney Diseases (NIDDK) at the National Institutes of Health (NIH) conducted a workshop on renal imaging to review the state-of-the-art and plan potential future endeavours [7]. Also in 2018, the UK Renal Imaging network (UKRIN) received a 3-year partnership grant to create a national infrastructure for quantitative renal MRI. In 2020, UKRIN has received funding for a 10 year cohort study in 500 CKD patients starting 1 sept 2020 (AFiRM study; principal investigator: Nick Selby, University of Nottingham).

iBEAt setup started in September of 2016 and is the first study to respond to the clinical need for systematically collected evidence at a larger scale and across institutions, with well-validated methods linking up the imaging findings with other sources of data so the added value can be identified. In that sense, iBEAt is inspired by the landmark study CRISP (Consortium for Radiologic Imaging Studies of Polycystic Kidney Disease) [36] - the first multi-centre cohort study exploring a quantitative MRI biomarker (TKV) in CKD and a foundation for the aforementioned FDA qualification of TKV. Like CRISP, iBEAt has built in a technical validation phase of the imaging biomarkers by including a repeatability study on all scanner types deployed in iBEAt, and by calibrating between-scanner differences through a travelling test object developed by the National Institute of Standards and Technology [37]. Also following the example of CRISP, iBEAt is committed to sharing the technical details of its imaging protocols and expertise in image processing and quality assurance – not only to facilitate the cost and setup of future studies but also to maximise alignment and future opportunities for pooling the data.

The integration of the ancillary studies into iBEAt will provide valuable information on the pathogenesis of DKD and the clinical utility of these imaging biomarkers. Crucially, they will explore the association of renal based imaging biomarkers against histopathological markers and different histological lesions of DKD, validating the imaging biomarkers and substantiating their clinical utility. MRI-based renal perfusion measurements will also be validated against PET renal perfusion measurements. The potential automation of the MRI image processing will streamline a labour-intensive process, thereby increasing the clinical applicability of the assessments. The microvascular assessments, including the examination of glycocalyx integrity and endothelial function, will provide invaluable information on the pathogenesis and heterogeneity of DKD, and may well aid the identification of individuals with fast progressing DKD. For example, we hypothesise that individuals with T2D and early signs of perturbations to the glycocalyx will be at an increased risk of DKD progression.

iBEAt has greatly benefitted in its setup from study documents and standard operating procedures (SOPs) provided by other investigators, in particular the PROVALID [27] and NEPTUNE [26] studies. In turn, iBEAt is committed to a “pay-it-forward” philosophy and will aim to share its study documentation and procedures widely for use by other investigators. iBEAt collaborators are also committed to maximise the opportunities for data sharing in order to increase the lifetime value of their research data as assets for human health and to do so timely, responsibly, with as few restrictions as possible, in a way consistent with the law, regulation and recognised good practice. Beyond data, iBEAt will aim to form a powerful resource for future biomarker discovery sources by collecting a rich collection of blood and urine samples in its central biobank. These will be made available for external investigators subject to formal application and approval by the iBEAt Steering Committee.

After a 2-year setup period the first study participant was recruited into iBEAt in October 2018. First results on technical validation of MRI methods on the reference scanner are expected at the end of 2020. The projected deadline for recruitment was 1 September 2020 and first results on the primary objective (cross-sectional analysis of baseline data) were expected to be made public in 2021. Completion of follow-up data was expected in September 2023, with results on the longitudinal analysis expected to be submitted for publication in 2024. These timelines will be affected by a pause in recruitment during the COVID-19 pandemic, but at submission of this manuscript (April 2020) the exact implications are not yet clear.

## Conclusion

There is an unmet need for biomarkers that can improve prediction of renal functional decline in DKD. Imaging based biomarkers have not yet been explored in this context and may be complementary to standard clinical markers. iBEAt, the largest DKD imaging study to date, will explore this hypothesis and may provide valuable insights into the progression and heterogeneity of DKD. The results may contribute to a more personalised approach to the management of DKD.

## Supplementary information

**Additional file 1: 1.1** MRI biomarkers. File type: PDF file. Title: List of primary MRI biomarkers. Description: A table listing the biomarkers that will be derived from the MRI data to address the primary objectives. **1.2** MRI acquisition protocol. PDF file. MRI acquisition protocol (reference scanner). MRI sequence parameters for the iBEAt protocol on the reference scanner (Siemens 3 T). **1.3** Renal ultrasound SOP. PDF file. Ultrasound Standard Operating Procedures. Standard operating procedures for Ultrasound scanning in iBEAt.

**Additional file 2: 2.1** Biofluid collection SOPs. PDF file. Biofluid collection protocol. The protocol for the collection of blood and urine samples within iBEAt. **2.2** SOPs Biofluid processing. PDF file. Biofluid processing protocol. The protocol for processing blood and urine samples within iBEAt. **2.3** Biofluid schematics. PDF file. iBEAt kit contents and biofluid processing schematics. Schematics of iBEAt collection kits, and processing and storage protocols for collected blood and urine samples within iBEAt.

**Additional file 3: 3.0** CRF Screening. PDF file. Study recruitment – prescreening / screening. Clinical record form for prescreening / screening data. **3.1** CRF Adherence Checklist. PDF file. Baseline visit (V1) – adherence checklist. Clinical record form documenting participant adherence to guidance for the baseline visit. **3.2** CRF Limited Clinical Exam. PDF file. Limited Clinical Exam. Clinical record form for clinical examination data including, for example, blood pressure, height and weight. **3.3** CRF Medical and Family Hx. PDF file. Baseline (V1) – Medical and family history V2. Clinical record form for medical and family history (version 2). **3.4** CRF Local Study Labs. PDF file. Baseline (V1) – local study labs. Clinical record form for laboratory measurements performed at recruiting centre. **3.5** CRF Routine Labs. PDF file. Baseline visit (V1) – labs. Clinical record form for documenting all available laboratory values in the year prior to the baseline visit. **3.6** CRF Medications. PDF file. Medication log. Clinical record form documenting all current medications. **3.7** CRF Ultrasound. PDF file. Baseline visit (V1) – Ultrasound. Clinical record form for the renal ultrasound measurements. **3.8** CRF Biosamples. PDF file. Study biosamples. Clinical record form / checklist documenting what biofluid samples were collected and processed for the iBEAt study.

**Additional file 4: 4.0** Biopsy SOP. PDF file. Biopsy and pathology SOPs. Protocol for storing and capturing meta-data regarding the renal biopsy tissue for the iBEAt study. (EXE 543 kb)

## Data Availability

iBEAt collaborators are committed to maximise the opportunities for data sharing at study completion in order to increase the lifetime value of their research data as assets for human health and to do so timely, responsibly, in a way consistent with the law, regulation and recognised good practice.

## Abbreviations

ACR: Albumin: creatinine ratio
ADPKD: Autosomal dominant polycystic kidney disease
AUC: Area under the curve
BEAt-DKD: Biomarker Enterprise to Attack Diabetic Kidney Disease
BMs: Biomarkers
BOLD: Blood oxygenation level dependent
CKD: Chronic kidney disease
CRC: Clinical research centre
CRF: Clinical record folder
CRISP: Consortium for Radiologic Imaging Studies of Polycystic Kidney Disease
DKD: Diabetic kidney disease
EFPIA: European Federation of Pharmaceutical Industries and Associations
eGFR: Estimated glomerular filtration rate
EMA: European Medicines Agency
FDA: Food and Drug Administration
HbA1c: Glycated haemoglobin
iBEAt: Prognostic Imaging Biomarkers for Diabetic Kidney Disease
IMI: Innovative Medicines Initiative
JDRF: Juvenile Diabetes Research Foundation
MR: Magnetic resonance
MRI: Magnetic resonance imaging
NIDDK: National Institute of Diabetes and Digestive and Kidney Diseases
NIH: National Institute of Health
NIHR: National Institute of Health Research
NIST: National Institute of Standards and Technology
PET: Positron-emission tomography
QA: Quality assurance
RBF: Renal blood flow
RI: Resistive index
ROC: Receiver operating characteristics curve
SOPs: Standard operating procedures
SIB: Swiss Institute of Bioinformatics
TKV: Total kidney volume
T1D: Type 1 diabetes
T2D: Type 2 diabetes
UMICH: University of Michigan
US: Ultrasound
