# Supplementary material for: Prognostic imaging biomarkers for diabetic kidney disease (iBEAt): study protocol
Source: BMC Nephrol. 2020 Jun 29;21:242. doi: 10.1186/s12882-020-01901-x (PMC7323369; doi:10.1186/s12882-020-01901-x)
Supplement: Supplementary file 1 — Additional file 1: 1.1 MRI biomarkers. File type: PDF file. Title: List of primary MRI biomarkers. Description: A table listing the biomarkers that will be derived from the MRI data to address the primary objectives. 1.2 MRI acquisition protocol. PDF file. MRI acquisition protocol (reference scanner). MRI sequence parameters for the iBEAt protocol on the reference scanner (Siemens 3 T). 1.3 Renal ultrasound SOP. PDF file. Ultrasound Standard Operating Procedures. Standard operating procedures for Ultrasound scanning in iBEAt. [file 12882_2020_1901_MOESM1_ESM.zip › Additional file 1.1 MRI biomarkersR1.pdf]

Prognostic Imaging Biomarkers for Diabetic Kidney Disease (iBEAt)

List of primary MRI biomarkers

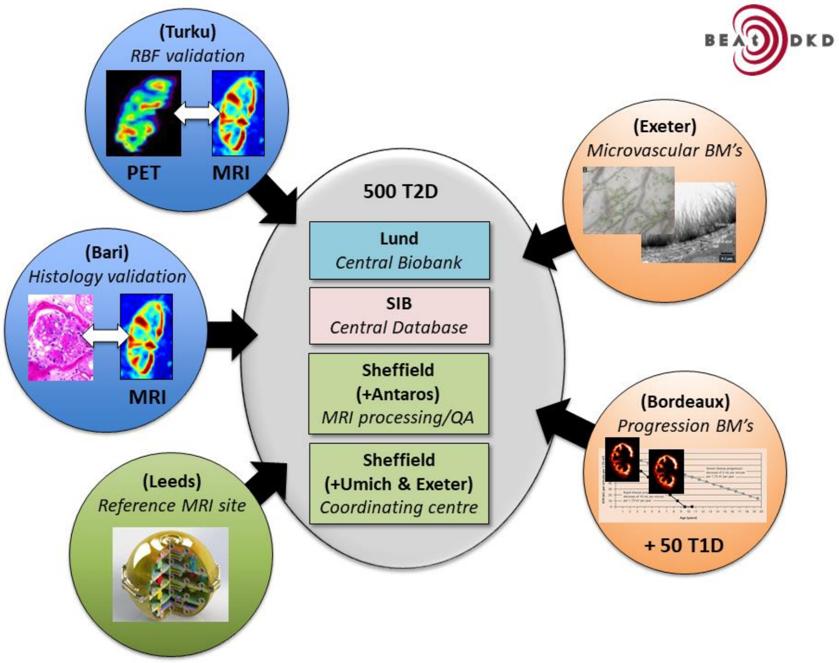

Version 1.0
09.10.2019

Authors: **Kanishka Sharma, Steven Sourbron**

Description: **List of biomarkers that will be derived from the MRI data to address the primary objectives**

Background:

In order to address the question of predicting disease progression in diabetic kidney disease, a dedicated MRI biomarker panel was developed interrogating body composition, renal morphology and tissue structure, hemodynamics and filtration. The panel will be derived by the central image processing centre from MRI data uploaded by recruiting sites on a central MRI data management platform (www.xnat.org). After image processing, MRI biomarkers will be integrated with all other data collected on the participant in the electronic data capture system of iBEAt (project-redcap.org).

The MRI biomarker panel is illustrated in the figure below, and listed in detail in the table on the following pages.

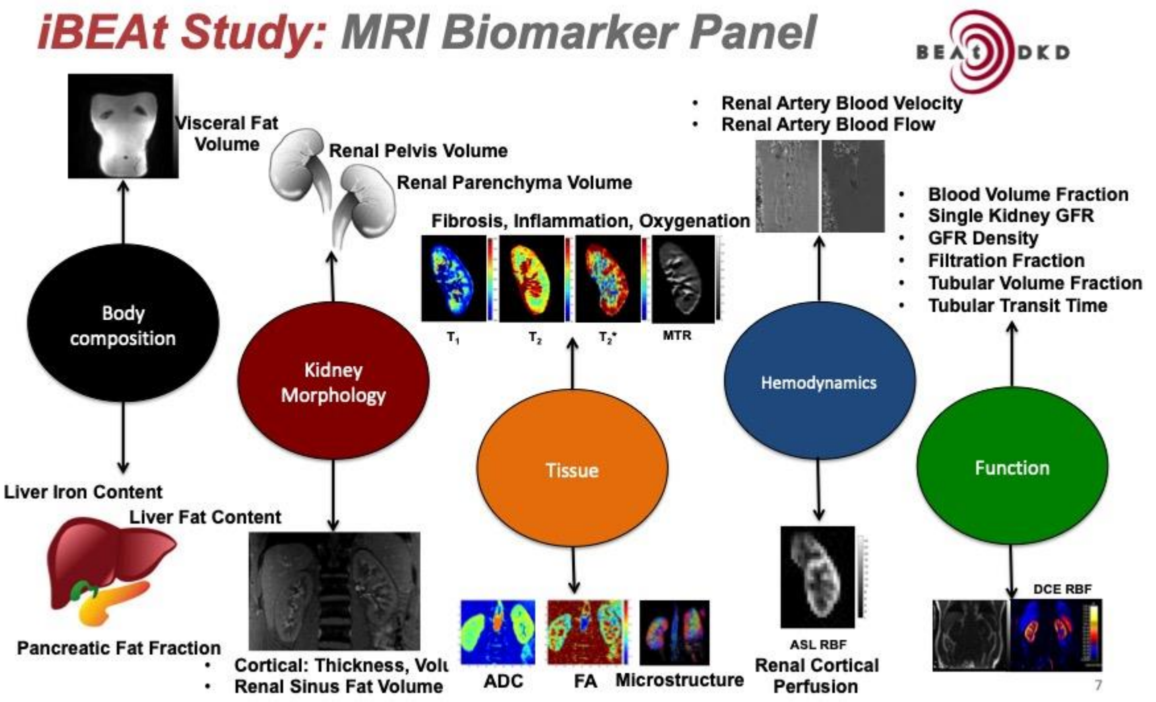

Reference:

Kanishka Sharma, Fotios Tagkalakis, Irvin Teh, Christopher Kelly, David Shelley, Virva Saunavaara, Dmitry Kuznetsov, Anil Karihaloo, Michael Mansfield, Mark Gilchrist, Roberto De Blasi, Mark Ibberson, Maria-Alexandra Olaru, Bernd Kühn, Nicolas Grenier, Steven Sourbron. *The multi-centre iBEAt study: A comprehensive multi-parametric MR imaging biomarker panel for Diabetic Kidney Disease. International Society for Magnetic Resonance in Medicine 2019*.

The table below shows all primary MRI biomarkers, including their name (2nd column), units (3rd column), the sequence in the iBEAt MRI protocol from which the biomarker is derived (4th column), and the general group of biomarkers it belongs to (5th column).

nr	Imaging Biomarker	Unit	Sequence Name	Category
1	Visceral Fat Volume	mL	T1w_abdomen_dixon_cor_bh	Body Composition
2	Pancreatic Fat Fraction	%	T2star_map_pancreas_tra_mbh	Body Composition
3	Liver Fat Fraction	%	T2star_map_pancreas_tra_mbh	Body Composition
4	Liver T2*	ms	T2star_map_pancreas_tra_mbh	Body Composition
5	Renal Sinus Fat Volume	mL	T1w_abdomen_dixon_cor_bh	Kidney Morphology
6	Left Kidney Parenchyma Volume	mL	T1w_abdomen_dixon_cor_bh	Kidney Morphology
7	Right Kidney Parenchyma Volume	mL	T1w_abdomen_dixon_cor_bh	Kidney Morphology
8	Left Cortical Thickness	cm	T1w_kidneys_cor-oblique_mbh	Kidney Morphology
9	Right Cortical Thickness	cm	T1w_kidneys_cor-oblique_mbh	Kidney Morphology
10	Left Cortical Volume	mL	T1w_kidneys_cor-oblique_mbh	Kidney Morphology
11	Right Cortical Volume	mL	T1w_kidneys_cor-oblique_mbh	Kidney Morphology
12	Left Cortical T1	ms	T1map_kidneys_cor-oblique_mbh	Tissue Characteristics
13	Right Cortical T1	ms	T1map_kidneys_cor-oblique_mbh	Tissue Characteristics
14	Left Medullary T1	ms	T1map_kidneys_cor-oblique_mbh	Tissue Characteristics
15	Right Medullary T1	ms	T1map_kidneys_cor-oblique_mbh	Tissue Characteristics
16	Left Cortical T2	ms	T2map_kidneys_cor-oblique_mbh	Tissue Characteristics
17	Right Cortical T2	ms	T2map_kidneys_cor-oblique_mbh	Tissue Characteristics
18	Left Medullary T2	ms	T2map_kidneys_cor-oblique_mbh	Tissue Characteristics
19	Right Medullary T2	ms	T2map_kidneys_cor-oblique_mbh	Tissue Characteristics

20	Left Cortical T2*	ms	T2star_map_kidneys_cor-oblique_mbh	Tissue Characteristics
21	Right Cortical T2*	ms	T2star_map_kidneys_cor-oblique_mbh	Tissue Characteristics
22	Left Medullary T2*	ms	T2star_map_kidneys_cor-oblique_mbh	Tissue Characteristics
23	Right Medullary T2*	ms	T2star_map_kidneys_cor-oblique_mbh	Tissue Characteristics
24	Left Cortical ADC	mm2/s	IVIM_kidneys_cor-oblique_fb	Tissue Characteristics
25	Right Cortical ADC	mm2/s	IVIM_kidneys_cor-oblique_fb	Tissue Characteristics
26	Left Medullary ADC	mm2/s	IVIM_kidneys_cor-oblique_fb	Tissue Characteristics
27	Right Medullary ADC	mm2/s	IVIM_kidneys_cor-oblique_fb	Tissue Characteristics
28	Left Cortical FA	%	DTI_kidneys_cor-oblique_fb	Tissue Characteristics
29	Right Cortical FA	%	DTI_kidneys_cor-oblique_fb	Tissue Characteristics
30	Left Medullary FA	%	DTI_kidneys_cor-oblique_fb	Tissue Characteristics
31	Right Medullary FA	%	DTI_kidneys_cor-oblique_fb	Tissue Characteristics
32	Left Cortical MTR	%	MT_kidneys_cor-oblique_bh	Tissue Characteristics
33	Right Cortical MTR	%	MT_kidneys_cor-oblique_bh	Tissue Characteristics
34	Left Medullary MTR	%	MT_kidneys_cor-oblique_bh	Tissue Characteristics
35	Right Medullary MTR	%	MT_kidneys_cor-oblique_bh	Tissue Characteristics
36	Left Renal Artery Mean Blood Velocity	cm/s	PC_RenalArtery_Left_EcgTrig_fb_120	Hemodynamics
37	Right Renal Artery Mean Blood Velocity	cm/s	PC_RenalArtery_Right_EcgTrig_fb_120	Hemodynamics
38	Left Renal Artery Peak Systolic Blood Velocity	cm/s	PC_RenalArtery_Left_EcgTrig_fb_120	Hemodynamics
39	Right Renal Artery Peak Systolic Blood Velocity	cm/s	PC_RenalArtery_Right_EcgTrig_fb_120	Hemodynamics
40	Left Renal Artery End Diastolic Blood Velocity	cm/s	PC_RenalArtery_Left_EcgTrig_fb_120	Hemodynamics
41	Right Renal Artery End Diastolic Blood Velocity	cm/s	PC_RenalArtery_Right_EcgTrig_fb_120	Hemodynamics
42	Left Renal Artery Mean Blood Flow	mL/min	PC_RenalArtery_Left_EcgTrig_fb_120	Hemodynamics

43	Right Renal Artery Mean Blood Flow	mL/min	PC_RenalArtery_Right_EcgTrig_fb_120	Hemodynamics
44	ASL Left Cortical Perfusion	mL/min /100mL	ASL_kidneys_pCASL_cor-oblique_fb	Hemodynamics
45	ASL Right Cortical Perfusion	mL/min /100mL	ASL_kidneys_pCASL_cor-oblique_fb	Hemodynamics
46	MRR Left Cortical Perfusion	mL/min /100mL	DCE_kidneys_cor-oblique_fb	Renal Function
47	MRR Right Cortical Perfusion	mL/min /100mL	DCE_kidneys_cor-oblique_fb	Renal Function
48	Left Kidney Filtration Fraction	%	DCE_kidneys_cor-oblique_fb	Renal Function
49	Right Kidney Filtration Fraction	%	DCE_kidneys_cor-oblique_fb	Renal Function
50	Left Cortical Tubular Volume Fraction	%	DCE_kidneys_cor-oblique_fb	Renal Function
51	Right Cortical Tubular Volume Fraction	%	DCE_kidneys_cor-oblique_fb	Renal Function
52	Left Medullary Tubular Volume Fraction	%	DCE_kidneys_cor-oblique_fb	Renal Function
53	Right Medullary Tubular Volume Fraction	%	DCE_kidneys_cor-oblique_fb	Renal Function
54	Left Cortical Blood Volume Fraction	%	DCE_kidneys_cor-oblique_fb	Renal Function
55	Right Cortical Blood Volume Fraction	%	DCE_kidneys_cor-oblique_fb	Renal Function
56	Left Medullary Blood Volume Fraction	%	DCE_kidneys_cor-oblique_fb	Renal Function
57	Right Medullary Blood Volume Fraction	%	DCE_kidneys_cor-oblique_fb	Renal Function
58	Left Renal Pelvis Volume	mL	T1w_abdomen_post_contrast_dixon_cor_bh	Kidney Morphology
59	Right Renal Pelvis Volume	mL	T1w_abdomen_post_contrast_dixon_cor_bh	Kidney Morphology
