# Supplementary material for: Prognostic imaging biomarkers for diabetic kidney disease (iBEAt): study protocol
Source: BMC Nephrol. 2020 Jun 29;21:242. doi: 10.1186/s12882-020-01901-x (PMC7323369; doi:10.1186/s12882-020-01901-x)
Supplement: Supplementary file 1 — Additional file 1: 1.1 MRI biomarkers. File type: PDF file. Title: List of primary MRI biomarkers. Description: A table listing the biomarkers that will be derived from the MRI data to address the primary objectives. 1.2 MRI acquisition protocol. PDF file. MRI acquisition protocol (reference scanner). MRI sequence parameters for the iBEAt protocol on the reference scanner (Siemens 3 T). 1.3 Renal ultrasound SOP. PDF file. Ultrasound Standard Operating Procedures. Standard operating procedures for Ultrasound scanning in iBEAt. [file 12882_2020_1901_MOESM1_ESM.zip › Additional file 1.2 MRI acquistion protocolR1.pdf]

Prognostic Imaging Biomarkers for Diabetic Kidney Disease (iBEAt)

MRI acquisition protocol (reference scanner)

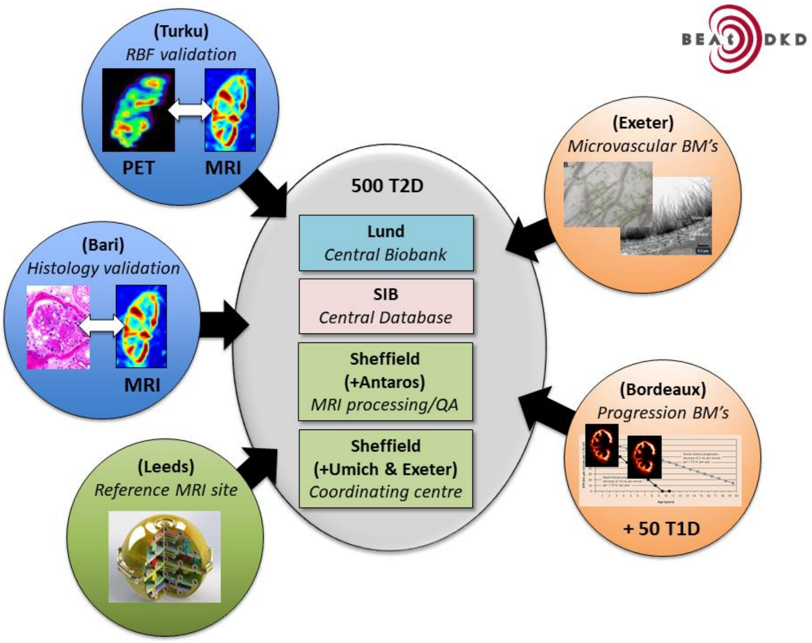

Version 10.6
09.10.2019

Authors: **Kanishka Sharma, Steven Sourbron**

Description: **MRI sequence parameters for the iBEAt protocol on the reference scanner**

- Vendor: Siemens
- Model: MAGNETOM PRISMA 3T
- Software version: VE11C
- Location: Advanced Imaging Centre, University of Leeds

Provenance:

The iBEAt MRI protocol was developed on the reference scanner in Leeds between August 2017 and March 2018. The final version number 10.6 has been used in Leeds since the start of iBEAt recruitment in October 2018, and has since been implemented and applied successfully for iBEAt MRI in Bordeaux (same scanner and software version). Translation to other vendors and models is currently in progress.

References:

1. Kanishka Sharma, Fotios Tagkalakis, Irvin Teh, David Shelley, Virva Saunavaara, Dmitry Kuznetsov, Anil Karihaloo, Michael Mansfield, Mark Gilchrist, Roberto De Blasi, Mark Ibberson, Nicolas Grenier, Steven Sourbron. *The iBEAT MRI protocol: Prognostic Imaging Biomarkers for Diabetic Kidney Disease. Am Soc Nephrol (San Diego 2018).*

Table of contents

\\Research

Abdominal

SS BEAT Kidney

iBEAT_DKDv10.6

localizer_bh_fix
localizer_bh_ISO
T2w_abdomen_haste_tra_mbh
T1w_abdomen_dixon_cor_bh
PC_RenalArtery_Right_EcgTrig_fb_120
PC_RenalArtery_Left_EcgTrig_fb_120
T2star_map_pancreas_tra_mbh
T1w_kidneys_cor-oblique_mbh
T1map_kidneys_cor-oblique_mbh
T2map_kidneys_cor-oblique_mbh
T2star_map_kidneys_cor-oblique_mbh
IVIM_kidneys_cor-oblique_fb
DTI_kidneys_cor-oblique_fb
MT_OFF_kidneys_cor-oblique_bh
MT_ON_kidneys_cor-oblique_bh
ASL_planning_bh
ASL_kidneys_pCASL_cor-oblique_fb
DCE_kidneys_cor-oblique_fb
T1w_abdomen_post_contrast_dixon_cor_bh

Copy References

localizer_bh_fix		
	None	
localizer_bh_ISO		
	None	
T2w_abdomen_haste_tra_mbh		
	None	
T1w_abdomen_dixon_cor_bh		
	None	
PC_RenalArtery_Right_EcgTrig_fb_120		
	None	
PC_RenalArtery_Left_EcgTrig_fb_120		
	None	
T2star_map_pancreas_tra_mbh		
	None	
T1w_kidneys_cor-oblique_mbh		
	None	
T1map_kidneys_cor-oblique_mbh		
	Source protocol step	T1w_kidneys_cor-oblique_mbh
	Copy Parameter group	Centre of slice group and saturation regions
T2map_kidneys_cor-oblique_mbh		
	Source protocol step	T1w_kidneys_cor-oblique_mbh
	Copy Parameter group	Centre of slice group and saturation regions
T2star_map_kidneys_cor-oblique_mbh		
	Source protocol step	T1w_kidneys_cor-oblique_mbh
	Copy Parameter group	Centre of slice group and saturation regions
IVIM_kidneys_cor-oblique_fb		
	Source protocol step	T1w_kidneys_cor-oblique_mbh
	Copy Parameter group	Centre of slice group and saturation regions
DTI_kidneys_cor-oblique_fb		
	Source protocol step	T1w_kidneys_cor-oblique_mbh
	Copy Parameter group	Centre of slice group and saturation regions
MT_OFF_kidneys_cor-oblique_bh		
	Source protocol step	T1w_kidneys_cor-oblique_mbh
	Copy Parameter group	Centre of slice group and saturation regions
MT_ON_kidneys_cor-oblique_bh		
	Source protocol step	T1w_kidneys_cor-oblique_mbh
	Copy Parameter group	Centre of slice group and saturation regions
ASL_planning_bh		
	None	
ASL_kidneys_pCASL_cor-oblique_fb		
	None	
DCE_kidneys_cor-oblique_fb		
	Source protocol step	T1w_kidneys_cor-oblique_mbh
	Copy Parameter group	Centre of slice group and saturation regions
T1w_abdomen_post_contrast_dixon_cor_bh		
	Source protocol step	T1w_abdomen_dixon_cor_bh
	Copy Parameter group	Everything

\\Research\Abdominal\SS BEAT Kidney\iBEAT_DKDv10.6\localizer_bh_fix
TA: 7.5 s PM: FIX Voxel size: 1.0×1.0×8.5 mmPAT: Off Rel. SNR: 1.00 : tfi

Properties

Prio recon	Off
Load images to viewer	Off
Inline movie	Off
Auto store images	On
Load images to stamp segments	On
Load images to graphic segments	On
Auto open inline display	Off
Auto close inline display	Off
Start measurement without further preparation	On
Wait for user to start	Off
Start measurements	Single measurement

Routine

Slice group	1
Slices	3
Dist. factor	500 %
Position	R2.1 A34.4 F25.1 mm
Orientation	Transversal
Phase enc. dir.	A >> P
Slice group	2
Slices	7
Dist. factor	250 %
Position	R1.0 A32.3 F26.0 mm
Orientation	Coronal
Phase enc. dir.	R >> L
Slice group	3
Slices	1
Dist. factor	20 %
Position	R41.8 A33.3 F24.1 mm
Orientation	Sagittal
Phase enc. dir.	A >> P
Slice group	4
Slices	1
Dist. factor	20 %
Position	L37.7 A28.4 F25.1 mm
Orientation	Sagittal
Phase enc. dir.	A >> P
AutoAlign	---
Phase oversampling	0 %
FoV read	400 mm
FoV phase	100.0 %
Slice thickness	8.5 mm
TR	498.73 ms
TE	1.05 ms
Averages	1
Concatenations	12
Filter	Distortion Corr.(2D), Prescan Normalize
Coil elements	BO1-3;SP1-4

Contrast - Common

TR	498.73 ms
TE	1.05 ms
TD	0 ms
Magn. preparation	None
Flip angle	31 deg
Fat suppr.	None
Wrap-up Magn.	None

Contrast - Dynamic

Averages	1
Averaging mode	Short term
Reconstruction	Magnitude
Measurements	1
Multiple series	Off

Resolution - Common

FoV read	400 mm
FoV phase	100.0 %
Slice thickness	8.5 mm
Base resolution	192
Phase resolution	100 %
Phase partial Fourier	Off
Trajectory	Cartesian
Interpolation	On

Resolution - iPAT

PAT mode	None

Resolution - Filter Image

Image Filter	Off
Distortion Corr.	On
Mode	2D
Unfiltered images	Off
Prescan Normalize	On
Unfiltered images	Off
Normalize	Off
B1 filter	Off

Resolution - Filter Rawdata

Raw filter	Off
Elliptical filter	Off
POCS	Off

Geometry - Common

Slice group	1
Slices	3
Dist. factor	500 %
Position	R2.1 A34.4 F25.1 mm
Orientation	Transversal
Phase enc. dir.	A >> P
Slice group	2
Slices	7
Dist. factor	250 %
Position	R1.0 A32.3 F26.0 mm
Orientation	Coronal
Phase enc. dir.	R >> L
Slice group	3
Slices	1
Dist. factor	20 %
Position	R41.8 A33.3 F24.1 mm
Orientation	Sagittal
Phase enc. dir.	A >> P
Slice group	4
Slices	1
Dist. factor	20 %
Position	L37.7 A28.4 F25.1 mm
Orientation	Sagittal
Phase enc. dir.	A >> P
FoV read	400 mm

Geometry - Common

FoV phase	100.0 %
Slice thickness	8.5 mm
TR	498.73 ms
Multi-slice mode	Sequential
Series	Interleaved
Concatenations	12

Geometry - AutoAlign

Slice group	1
Position	R2.1 A34.4 F25.1 mm
Orientation	Transversal
Phase enc. dir.	A >> P
Slice group	2
Position	R1.0 A32.3 F26.0 mm
Orientation	Coronal
Phase enc. dir.	R >> L
Slice group	3
Position	R41.8 A33.3 F24.1 mm
Orientation	Sagittal
Phase enc. dir.	A >> P
Slice group	4
Position	L37.7 A28.4 F25.1 mm
Orientation	Sagittal
Phase enc. dir.	A >> P
AutoAlign	---
Initial Position	R2.1 A34.4 F25.1
R	2.1 mm
A	34.4 mm F
25.1 mm Initial Rotation	
0.00 deg Initial Orientation	
Transversal	

Geometry - Saturation

Fat suppr.	None
Wrap-up Magn.	None
Special sat.	None

Geometry - Navigator

Geometry - Tim Planning Suite

Set-n-Go Protocol	Off
Table position	H
Table position	0 mm
Inline Composing	Off

System - Miscellaneous

Positioning mode	FIX
Table position	H
Table position	0 mm
MSMA	S - C - T
Sagittal	R >> L
Coronal	A >> P
Transversal	H >> F
Coil Combine Mode	Adaptive Combine
Save uncombined	Off
Matrix Optimization	Off
Coil Focus	Flat
AutoAlign	---
Coil Select Mode	Default

System - Adjustments

B0 Shim mode	Standard
B1 Shim mode	TrueForm
Adjust with body coil	On

System - Adjustments

Confirm freq. adjustment	Off
Assume Dominant Fat	Off
Assume Silicone	Off
Adjustment Tolerance	Auto

System - Adjust Volume

Position	R1.5 A31.4 F25.1 mm
Orientation	Sagittal
Rotation	90.00 deg
F >> H	402 mm
A >> P	406 mm
R >> L	402 mm
Reset	Off

System - pTx Volumes

B1 Shim mode	TrueForm
Excitation	Slice-sel.

System - Tx/Rx

Frequency 1H	123.255690 MHz
Correction factor	1
Gain	High
Img. Scale Cor.	1.000
Reset	Off
? Ref. amplitude 1H	0.000 V

Physio - Signal1

1st Signal/Mode	None
TR	498.73 ms
Concatenations	12
Segments	192

Physio - Cardiac

Tagging	None
Magn. preparation	None
Fat suppr.	None
Dark blood	Off
FoV read	400 mm
FoV phase	100.0 %
Phase resolution	100 %
Cine	Off
Trajectory	Cartesian
Dummy heartbeats	0

Physio - PACE

Resp. control	Off
Concatenations	12

Inline - Common

Subtract	Off
Measurements	1
StdDev	Off
Save original images	On

Inline - Cardiac

Inline Evaluation	Off
Magn. preparation	None
Contrasts	1
TE	1.05 ms
TR	498.73 ms
Save original images	On

Inline - MIP

MIP-Sag	Off
MIP-Cor	Off
MIP-Tra	Off
MIP-Time	Off
Save original images	On

Inline - Composing

Inline Composing	Off
Distortion Corr.	On
Mode	2D
Unfiltered images	Off

Sequence - Part 1

Introduction	On
Dimension	2D
Reordering	Linear
Asymmetric echo	Weak
Contrasts	1
Optimization	Min. TE TR
Multi-slice mode	Sequential
Echo spacing	2.5 ms
Sequence type	Trufi
Bandwidth	1132 Hz/Px

Sequence - Part 2

Define	Shots
Shots per slice	1
Segments	192
Trufi delta freq.	0 Hz RF
pulse type	Fast
Gradient mode	Fast
Excitation	Slice-sel.
Flip angle mode	Constant
Cine	Off

Sequence - Assistant

Mode	Min flip angle
Min flip angle	35 deg
Allowed delay	0 s

\\Research\Abdominal\SS BEAT Kidney\iBEAT_DKDv10.6\localizer_bh_ISO
TA: 1:17 PM: ISO Voxel size: 1.0×1.0×4.0 mmPAT: Off Rel. SNR: 1.00 : tfi

Properties

Prio recon	Off
Load images to viewer	Off
Inline movie	Off
Auto store images	On
Load images to stamp segments	On
Load images to graphic segments	On
Auto open inline display	Off
Auto close inline display	Off
Start measurement without further preparation	Off
Wait for user to start	Off
Start measurements	Single measurement

Routine

Slice group	1
Slices	22
Dist. factor	10 %
Position	L23.1 P18.2 H1.5 mm
Orientation	Transversal
Phase enc. dir.	A >> P
Slice group	2
Slices	20
Dist. factor	10 %
Position	L18.0 P18.6 H1.4 mm
Orientation	Coronal
Phase enc. dir.	R >> L
Slice group	3
Slices	12
Dist. factor	10 %
Position	R2.2 P23.4 F0.5 mm
Orientation	Sagittal
Phase enc. dir.	A >> P
Slice group	4
Slices	12
Dist. factor	10 %
Position	L54.8 P24.7 H0.0 mm
Orientation	Sagittal
Phase enc. dir.	A >> P
AutoAlign	---
Phase oversampling	0 %
FoV read	400 mm
FoV phase	100.0 %
Slice thickness	4.0 mm
TR	567.41 ms
TE	1.3 ms
Averages	2
Concatenations	6
Filter	Distortion Corr.(2D), Prescan Normalize
Coil elements	BO1-3;SP6-8

Contrast - Common

TR	567.41 ms
TE	1.3 ms
Magn. preparation	None
Flip angle	26 deg
Fat suppr.	None
Wrap-up Magn.	None

Contrast - Dynamic

Averages	2
Averaging mode	Short term
Reconstruction	Magnitude
Measurements	1
Multiple series	Off

Resolution - Common

FoV read	400 mm
FoV phase	100.0 %
Slice thickness	4.0 mm
Base resolution	192
Phase resolution	100 %
Phase partial Fourier	Off
Trajectory	Cartesian
Interpolation	On

Resolution - iPAT

PAT mode	None

Resolution - Filter Image

Image Filter	Off
Distortion Corr.	On
Mode	2D
Unfiltered images	Off
Prescan Normalize	On
Unfiltered images	Off
Normalize	Off
B1 filter	Off

Resolution - Filter Rawdata

Raw filter	Off
Elliptical filter	Off
POCS	Off

Geometry - Common

Slice group	1
Slices	22
Dist. factor	10 %
Position	L23.1 P18.2 H1.5 mm
Orientation	Transversal
Phase enc. dir.	A >> P
Slice group	2
Slices	20
Dist. factor	10 %
Position	L18.0 P18.6 H1.4 mm
Orientation	Coronal
Phase enc. dir.	R >> L
Slice group	3
Slices	12
Dist. factor	10 %
Position	R2.2 P23.4 F0.5 mm
Orientation	Sagittal
Phase enc. dir.	A >> P
Slice group	4
Slices	12
Dist. factor	10 %
Position	L54.8 P24.7 H0.0 mm
Orientation	Sagittal
Phase enc. dir.	A >> P
FoV read	400 mm
FoV phase	100.0 %

Geometry - Common

Slice thickness	4.0 mm
TR	567.41 ms
Multi-slice mode	Sequential
Series	Interl. in B.-h.
Concatenations	6

Geometry - AutoAlign

Slice group	1
Position	L23.1 P18.2 H1.5 mm
Orientation	Transversal
Phase enc. dir.	A >> P
Slice group	2
Position	L18.0 P18.6 H1.4 mm
Orientation	Coronal
Phase enc. dir.	R >> L
Slice group	3
Position	R2.2 P23.4 F0.5 mm
Orientation	Sagittal
Phase enc. dir.	A >> P
Slice group	4
Position	L54.8 P24.7 H0.0 mm
Orientation	Sagittal
Phase enc. dir.	A >> P
AutoAlign	---
Initial Position	L23.1 P18.2 H1.5
L	23.1 mm
P	18.2 mm
H	1.5 mm
Initial Rotation	0.00 deg
Initial Orientation	Transversal

Geometry - Saturation

Fat suppr.	None
Wrap-up Magn.	None
Special sat.	None

Geometry - Navigator

Geometry - Tim Planning Suite

Set-n-Go Protocol	Off
Table position	H
Table position	0 mm
Inline Composing	Off

System - Miscellaneous

Positioning mode	ISO
Table position	H
Table position	0 mm
MSMA	S - C - T
Sagittal	R >> L
Coronal	A >> P
Transversal	H >> F
Coil Combine Mode	Adaptive Combine
Save uncombined	Off
Matrix Optimization	Off
Coil Focus	Flat
AutoAlign	---
Coil Select Mode	Default

System - Adjustments

B0 Shim mode	Standard
B1 Shim mode	TrueForm
Adjust with body coil	On
Confirm freq. adjustment	Off

System - Adjustments

Assume Dominant Fat	Off
Assume Silicone	Off
Adjustment Tolerance	Auto

System - Adjust Volume

Position	L20.5 P21.4 H0.5 mm
Orientation	Transversal
Rotation	90.00 deg
R >> L	406 mm
A >> P	407 mm
F >> H	402 mm
Reset	Off

System - pTx Volumes

B1 Shim mode	TrueForm
Excitation	Slice-sel.

System - Tx/Rx

Frequency 1H	123.255690 MHz
Correction factor	1
Gain	High
Img. Scale Cor.	1.000
Reset	Off
? Ref. amplitude 1H	0.000 V

Physio - Signal1

1st Signal/Mode	None
TR	567.41 ms
Concatenations	6
Segments	192

Physio - Cardiac

Tagging	None
Magn. preparation	None
Fat suppr.	None
Dark blood	Off
FoV read	400 mm
FoV phase	100.0 %
Phase resolution	100 %
Cine	Off
Trajectory	Cartesian
Dummy heartbeats	0

Physio - PACE

Resp. control	Breath-hold
Concatenations	6

Inline - Common

Subtract	Off
Measurements	1
StdDev	Off
Save original images	On

Inline - Cardiac

Inline Evaluation	Off
Magn. preparation	None
Contrasts	1
TE	1.3 ms
TR	567.41 ms
Save original images	On

Inline - MIP

MIP-Sag	Off

Inline - MIP

MIP-Cor	Off
MIP-Tra	Off
MIP-Time	Off
Save original images	On

Inline - Composing

Inline Composing	Off
Distortion Corr.	On
Mode	2D
Unfiltered images	Off

Sequence - Part 1

Introduction	On
Dimension	2D
Reordering	Linear
Asymmetric echo	Weak
Contrasts	1
Optimization	Min. TE TR
Multi-slice mode	Sequential
Echo spacing	2.8 ms
Sequence type	Trufi
Bandwidth	1132 Hz/Px

Sequence - Part 2

Define	Shots
Shots per slice	1
Segments	192
Trufi delta freq.	0 Hz RF
pulse type	Fast
Gradient mode	Fast
Excitation	Slice-sel.
Flip angle mode	Constant
Cine	Off

Sequence - Assistant

Mode	Min flip angle
Min flip angle	35 deg
Allowed delay	0 s

\\Research\Abdominal\SS BEAT Kidney\iBEAT_DKDv10.6\T2w_abdomen_haste_tra_mbh
TA: 0:57 PM: REF Voxel size: 1.3×1.3×5.0 mmPAT: 2 Rel. SNR: 1.00 : h

Properties

Prio recon	Off
Load images to viewer	Off
Inline movie	Off
Auto store images	On
Load images to stamp segments	On
Load images to graphic segments	On
Auto open inline display	On
Auto close inline display	On
Start measurement without further preparation	Off
Wait for user to start	Off
Start measurements	Single measurement

Routine

Slice group	1
Slices	35
Dist. factor	20 %
Position	L17.7 P3.2 H85.7 mm
Orientation	Transversal
Phase enc. dir.	A >> P
AutoAlign	---
Phase oversampling	50 %
FoV read	400 mm
FoV phase	100.0 %
Slice thickness	5.0 mm
TR	1400.0 ms
TE	91 ms
Averages	1
Concatenations	3
Filter	Distortion Corr.(2D), Prescan Normalize
Coil elements	BO2,3;SP7,8

Contrast - Common

TR	1400.0 ms
TE	91 ms
MTC	Off
Magn. preparation	None
Flip angle	101 deg
Fat suppr.	None
Water suppr.	None
Restore magn.	Off

Contrast - Dynamic

Averages	1
Averaging mode	Long term
Reconstruction	Magnitude
Measurements	1
Multiple series	Off

Resolution - Common

FoV read	400 mm
FoV phase	100.0 %
Slice thickness	5.0 mm
Base resolution	320
Phase resolution	100 %
Phase partial Fourier	4/8
Interpolation	Off

Resolution - iPAT

PAT mode	GRAPPA
Accel. factor PE	2
Ref. lines PE	42
Reference scan mode	Integrated

Resolution - Filter Image

Image Filter	Off
Distortion Corr.	On
Mode	2D
Unfiltered images	Off
Prescan Normalize	On
Unfiltered images	Off
Normalize	Off
B1 filter	Off

Resolution - Filter Rawdata

Raw filter	Off
Elliptical filter	Off

Geometry - Common

Slice group	1
Slices	35
Dist. factor	20 %
Position	L17.7 P3.2 H85.7 mm
Orientation	Transversal
Phase enc. dir.	A >> P
FoV read	400 mm
FoV phase	100.0 %
Slice thickness	5.0 mm
TR	1400.0 ms
Multi-slice mode	Single shot
Series	Interl. in B.-h.
Concatenations	3

Geometry - AutoAlign

Slice group	1
Position	L17.7 P3.2 H85.7 mm
Orientation	Transversal
Phase enc. dir.	A >> P
AutoAlign	---
Initial Position	L17.7 P3.2 H21.7
L	17.7 mm
P	3.2 mm
H	21.7 mm
Initial Rotation	0.00 deg
Initial Orientation	Transversal

Geometry - Saturation

Fat suppr.	None
Water suppr.	None
Restore magn.	Off
Special sat.	Parallel F/H
Gap	10 mm
Thickness	60 mm

Geometry - Navigator

Geometry - Tim Planning Suite

Set-n-Go Protocol	Off
Table position	H

Geometry - Tim Planning Suite

Table position	64 mm
Inline Composing	Off

System - Miscellaneous

Positioning mode	REF
Table position	H
Table position	64 mm
MSMA	S - C - T
Sagittal	R >> L
Coronal	A >> P
Transversal	H >> F
Coil Combine Mode	Adaptive Combine
Save uncombined	Off
Matrix Optimization	Off
Coil Focus	Flat
AutoAlign	---
Coil Select Mode	Default

System - Adjustments

B0 Shim mode	Standard
B1 Shim mode	TrueForm
Adjust with body coil	Off
Confirm freq. adjustment	Off
Assume Dominant Fat	Off
Assume Silicone	Off
Adjustment Tolerance	Auto

System - Adjust Volume

Position	L17.7 P3.2 H85.7 mm
Orientation	Transversal
Rotation	0.00 deg
A >> P	400 mm
R >> L	400 mm
F >> H	209 mm
Reset	Off

System - pTx Volumes

B1 Shim mode	TrueForm

System - Tx/Rx

Frequency 1H	123.255690 MHz
Correction factor	1
Gain	High
Img. Scale Cor.	1.000
Reset	Off
? Ref. amplitude 1H	0.000 V

Physio - Signal1

1st Signal/Mode	None
TR	1400.0 ms
Concatenations	3

Physio - Cardiac

Magn. preparation	None
Fat suppr.	None
Dark blood	Off
FoV read	400 mm
FoV phase	100.0 %
Phase resolution	100 %

Physio - PACE

Resp. control	Breath-hold
Concatenations	3

Inline - Common

Subtract	Off
Measurements	1
StdDev	Off
Save original images	On

Inline - MIP

MIP-Sag	Off
MIP-Cor	Off
MIP-Tra	Off
MIP-Time	Off
Save original images	On

Inline - Composing

Inline Composing	Off
Distortion Corr.	On
Mode	2D
Unfiltered images	Off

Sequence - Part 1

Introduction	Off
Dimension	2D
Contrasts	1
Flow comp.	No
Multi-slice mode	Single shot
Echo spacing	3.94 ms
Bandwidth	710 Hz/Px

Sequence - Part 2

RF pulse type	Fast
Gradient mode	Fast
Hyperecho	Off
Turbo factor	320

Sequence - Assistant

Mode	Off
Allowed delay	30 s

\\Research\Abdominal\SS BEAT Kidney\iBEAT_DKDv10.6\T1w_abdomen_dixon_cor_bh
TA: 0:20 PM: REF Voxel size: 1.3×1.3×1.5 mmPAT: 6 Rel. SNR: 1.00 : fl

Properties

Prio recon	Off
Load images to viewer	On
Inline movie	Off
Auto store images	On
Load images to stamp segments	On
Load images to graphic segments	On
Auto open inline display	On
Auto close inline display	Off
Start measurement without further preparation	Off
Wait for user to start	On
Start measurements	Single measurement

Routine

Slab group	1
Slabs	1
Dist. factor	20 %
Position	L15.3 P10.3 H70.1 mm
Orientation	Coronal
Phase enc. dir.	R >> L
AutoAlign	---
Phase oversampling	20 %
Slice oversampling	22.2 %
Slices per slab	144
FoV read	400 mm
FoV phase	100.0 %
Slice thickness	1.5 mm
TR	4.01 ms
TE 1	1.34 ms
TE 2	2.57 ms
Averages	1
Concatenations	1
Filter	Distortion Corr.(2D), Prescan Normalize
Coil elements	BO1-3;SP6-8

Contrast - Common

TR	4.01 ms
TE 1	1.34 ms
TE 2	2.57 ms
Flip angle	9.0 deg
Fat suppr.	None
Water suppr.	None
Dixon	On

Contrast - Dynamic

Averages	1
Averaging mode	Long term
Reconstruction	Magnitude
Measurements	1
Multiple series	Off

Resolution - Common

FoV read	400 mm
FoV phase	100.0 %
Slice thickness	1.5 mm
Base resolution	320
Phase resolution	80 %
Slice resolution	60 %
Phase partial Fourier	7/8

Resolution - Common

Slice partial Fourier	7/8
Trajectory	Cartesian
View sharing	Off
Interpolation	Off

Resolution - iPAT

PAT mode	CAIPIRINHA
Accel. factor PE	3
Ref. lines PE	24
Accel. factor 3D	2
Ref. lines 3D	28
Reordering Shift 3D	0
Reference scan mode	GRE/separate
CAIPIRINHA mode	Body Tra
Total PAT factor	6

Resolution - Filter Image

Image Filter	Off
Distortion Corr.	On
Mode	2D
Unfiltered images	Off
Prescan Normalize	On
Unfiltered images	Off
Normalize	Off
B1 filter	Off

Resolution - Filter Rawdata

Raw filter	Off
Elliptical filter	Off
POCS	Off

Geometry - Common

Slab group	1
Slabs	1
Dist. factor	20 %
Position	L15.3 P10.3 H70.1 mm
Orientation	Coronal
Phase enc. dir.	R >> L
Slice oversampling	22.2 %
Slices per slab	144
FoV read	400 mm
FoV phase	100.0 %
Slice thickness	1.5 mm
TR	4.01 ms
Multi-slice mode	Sequential
Series	Ascending
Concatenations	1

Geometry - AutoAlign

Slab group	1
Position	L15.3 P10.3 H70.1 mm
Orientation	Coronal
Phase enc. dir.	R >> L
AutoAlign	---
Initial Position	L15.3 P10.3 H6.1
L	15.3 mm
P	10.3 mm
H	6.1 mm
Initial Rotation	0.00 deg
Initial Orientation	Coronal

Geometry - Saturation

Fat suppr.	None
Water suppr.	None
Dixon	On
Special sat.	None

Geometry - Tim Planning Suite

Set-n-Go Protocol	Off
Table position	H
Table position	64 mm
Inline Composing	Off

System - Miscellaneous

Positioning mode	REF
Table position	H
Table position	64 mm
MSMA	S - C - T
Sagittal	R >> L
Coronal	A >> P
Transversal	H >> F
Coil Combine Mode	Adaptive Combine
Save uncombined	Off
Matrix Optimization	Off
Coil Focus	Flat
AutoAlign	---
Coil Select Mode	Default

System - Adjustments

B0 Shim mode	Standard
B1 Shim mode	TrueForm
Adjust with body coil	Off
Confirm freq. adjustment	Off
Assume Dominant Fat	Off
Assume Silicone	Off
Adjustment Tolerance	Auto

System - Adjust Volume

Position	L15.3 P10.3 H70.1 mm
Orientation	Coronal
Rotation	0.00 deg
R >> L	400 mm
F >> H	400 mm
A >> P	216 mm
Reset	Off

System - pTx Volumes

B1 Shim mode	TrueForm
Excitation	Slab-sel.

System - Tx/Rx

Frequency 1H	123.255690 MHz
Correction factor	1
Gain	Low
Img. Scale Cor.	1.000
Reset	Off
? Ref. amplitude 1H	0.000 V

Physio - PACE

Resp. control	Breath-hold
Concatenations	1

Inline - Common

View sharing	Off
Flip angle	9.0 deg

Inline - Common

Measurements	1
Burn time-to-center	Off
Temporal interpolation	1
3D centric reordering	Off
Time to center	10.1 s

Inline - Inline

Subtract	Off
Measurements	1
StdDev	Off
Liver registration	Off
Save original images	On

Inline - MIP

MIP-Sag	Off
MIP-Cor	Off
MIP-Tra	Off
MIP-Time	Off
Save original images	On

Inline - Soft Tissue

Wash - In	Off
Wash - Out	Off
TTP	Off
PEI	Off
MIP - time	Off
Measurements	1

Inline - Composing

Inline Composing	Off
Distortion Corr.	On
Mode	2D
Unfiltered images	Off

Inline - MapIt

Save original images	On
MapIt	None
Flip angle	9.0 deg
Measurements	1
Contrasts	2
TR	4.01 ms
TE 1	1.34 ms
TE 2	2.57 ms

Sequence - Part 1

Introduction	Off
Dimension	3D
Elliptical scanning	Off
Asymmetric echo	Weak
Contrasts	2
Readout mode	Bipolar
Optimization	Opp/In
Multi-slice mode	Sequential
Bandwidth 1	820 Hz/Px
Bandwidth 2	1040 Hz/Px

Sequence - Part 2

RF pulse type	Fast
Gradient mode	Fast
Excitation	Slab-sel.
RF spoiling	On
Incr. Gradient spoiling	On

Sequence - Assistant

Mode	Off
Allowed delay	60 s

\\Research\Abdominal\SS BEAT Kidney\iBEAT_DKDv10.6\PC_RenalArtery_Right_EcgTrig_fb_120
TA: 1:40 PM: REF Voxel size: 0.6×0.6×6.0 mmPAT: 2 Rel. SNR: 1.00 : fl_r

Properties

Prio recon	Off
Load images to viewer	On
Inline movie	Off
Auto store images	On
Load images to stamp segments	On
Load images to graphic segments	On
Auto open inline display	Off
Auto close inline display	Off
Start measurement without further preparation	Off
Wait for user to start	Off
Start measurements	Single measurement

Routine

Slice group	1
Slices	1
Dist. factor	20 %
Position	L5.3 A3.7 H3.6 mm
Orientation	S > C31.6 > T-12.4
Phase enc. dir.	A >> P
AutoAlign	---
Phase oversampling	11 %
FoV read	350 mm
FoV phase	68.8 %
Slice thickness	6.0 mm
TR	40.48 ms
TE	2.74 ms
Averages	5
Concatenations	1
Filter	Distortion Corr.(2D)
Coil elements	BO1-3;SP6-8

Contrast - Common

TR	40.48 ms
TE	2.74 ms
TD	0 ms
Flip angle	25 deg
Wrap-up Magn.	None

Contrast - Dynamic

Averages	5
Averaging mode	Long term
Reconstruction	Magn./Phase
Measurements	1
Multiple series	Off

Resolution - Common

FoV read	350 mm
FoV phase	68.8 %
Slice thickness	6.0 mm
Base resolution	288
Phase resolution	70 %
Phase partial Fourier	Off
Trajectory	Cartesian
View sharing	Off
Interpolation	On

Resolution - iPAT

PAT mode	GRAPPA
Accel. factor PE	2

Resolution - iPAT

Ref. lines PE	24
Reference scan mode	GRE/separate

Resolution - Filter Image

Image Filter	Off
Distortion Corr.	On
Mode	2D
Unfiltered images	Off
Prescan Normalize	Off
Normalize	Off
B1 filter	Off

Resolution - Filter Rawdata

Raw filter	Off
Elliptical filter	Off
POCS	Off

Geometry - Common

Slice group	1
Slices	1
Dist. factor	20 %
Position	L5.3 A3.7 H3.6 mm
Orientation	S > C31.6 > T-12.4
Phase enc. dir.	A >> P
FoV read	350 mm
FoV phase	68.8 %
Slice thickness	6.0 mm
TR	40.48 ms
Multi-slice mode	Sequential
Series	Ascending
Concatenations	1

Geometry - AutoAlign

Slice group	1
Position	L5.3 A3.7 H3.6 mm
Orientation	S > C31.6 > T-12.4
Phase enc. dir.	A >> P
AutoAlign	---
Initial Position	L5.3 A3.7 H3.6
L	5.3 mm
A	3.7 mm
H	3.6 mm
Initial Rotation	3.40 deg
Initial Orientation	S > C
S > C	31.6
> T	-12.4

Geometry - Saturation

Wrap-up Magn.	None
Special sat.	None

Geometry - Navigator

Geometry - Tim Planning Suite

Set-n-Go Protocol	Off
Table position	H
Table position	0 mm
Inline Composing	Off

System - Miscellaneous

Positioning mode	REF
Table position	H
Table position	0 mm
MSMA	S - C - T
Sagittal	R >> L
Coronal	A >> P
Transversal	F >> H
Coil Combine Mode	Sum of Squares
Matrix Optimization	Off
Coil Focus	Flat
AutoAlign	---
Coil Select Mode	Default

System - Adjustments

B0 Shim mode	Tune up
B1 Shim mode	TrueForm
Adjust with body coil	Off
Confirm freq. adjustment	Off
Assume Dominant Fat	Off
Assume Silicone	Off
Adjustment Tolerance	Auto

System - Adjust Volume

Position	Isocenter
Orientation	Transversal
Rotation	0.00 deg
A >> P	263 mm
R >> L	350 mm
F >> H	350 mm
Reset	Off

System - pTx Volumes

B1 Shim mode	TrueForm
Excitation	Slice-sel.

System - Tx/Rx

Frequency 1H	123.255690 MHz
Correction factor	1
Gain	High
Img. Scale Cor.	1.000
Reset	Off
? Ref. amplitude 1H	0.000 V

Physio - Signal1

1st Signal/Mode	ECG/Retro
Average cycle	990 ± 10 ms
Average cycle	1000 ± 10 ms
Calculated phases	20
TR	40.48 ms
Concatenations	1
Segments	4
Arrhythmia detection	None

Physio - PACE

Resp. control	Off
Concatenations	1

Angio - Common

Flow mode	Single dir.
Encodings	1
Velocity enc.	120 cm/s
Direction	Through plane
Rephased images	On

Angio - Common

Magnitude images	On
Magnitude sum	Off
Phase images	On

Angio - Inline

Subtract	Off
Measurements	1
StdDev	Off
Save original images	On

Angio - Cardiac

Inline Evaluation	Off
TE	2.74 ms
TR	40.48 ms
Save original images	On

Angio - MIP

MIP-Sag	Off
MIP-Cor	Off
MIP-Tra	Off
MIP-Time	Off
Save original images	On

Angio - Composing

Inline Composing	Off
Distortion Corr.	On
Mode	2D
Unfiltered images	Off

Sequence - Part 1

Introduction	Off
Dimension	2D
Reordering	Linear
Asymmetric echo	Strong
Flow comp.	Yes
Optimization	Min. TE TR
Multi-slice mode	Sequential
Echo spacing	5.1 ms
Sequence type	Gre
Bandwidth	445 Hz/Px

Sequence - Part 2

Define	Segments
Segments	4
RF pulse type	Normal
Gradient mode	Normal
Excitation	Slice-sel.
Flip angle mode	Constant
RF spoiling	On
Phase Enc. Rewinder	On
Cine	On

Sequence - Assistant

Mode	Off
Allowed delay	0 s

\\Research\Abdominal\SS BEAT Kidney\iBEAT_DKDv10.6\PC_RenalArtery_Left_EcgTrig_fb_120
TA: 1:40 PM: REF Voxel size: 0.6×0.6×6.0 mmPAT: 2 Rel. SNR: 1.00 : fl_r

Properties

Prio recon	Off
Load images to viewer	On
Inline movie	Off
Auto store images	On
Load images to stamp segments	On
Load images to graphic segments	On
Auto open inline display	Off
Auto close inline display	Off
Start measurement without further preparation	Off
Wait for user to start	Off
Start measurements	Single measurement

Routine

Slice group	1
Slices	1
Dist. factor	20 %
Position	L40.2 P0.7 F1.9 mm
Orientation	S > C-35.9 > T7.3
Phase enc. dir.	A >> P
AutoAlign	---
Phase oversampling	11 %
FoV read	350 mm
FoV phase	68.8 %
Slice thickness	6.0 mm
TR	40.48 ms
TE	2.74 ms
Averages	5
Concatenations	1
Filter	Distortion Corr.(2D)
Coil elements	BO1-3;SP6-8

Contrast - Common

TR	40.48 ms
TE	2.74 ms
TD	0 ms
Flip angle	25 deg
Wrap-up Magn.	None

Contrast - Dynamic

Averages	5
Averaging mode	Long term
Reconstruction	Magn./Phase
Measurements	1
Multiple series	Off

Resolution - Common

FoV read	350 mm
FoV phase	68.8 %
Slice thickness	6.0 mm
Base resolution	288
Phase resolution	70 %
Phase partial Fourier	Off
Trajectory	Cartesian
View sharing	Off
Interpolation	On

Resolution - iPAT

PAT mode	GRAPPA
Accel. factor PE	2

Resolution - iPAT

Ref. lines PE	24
Reference scan mode	GRE/separate

Resolution - Filter Image

Image Filter	Off
Distortion Corr.	On
Mode	2D
Unfiltered images	Off
Prescan Normalize	Off
Normalize	Off
B1 filter	Off

Resolution - Filter Rawdata

Raw filter	Off
Elliptical filter	Off
POCS	Off

Geometry - Common

Slice group	1
Slices	1
Dist. factor	20 %
Position	L40.2 P0.7 F1.9 mm
Orientation	S > C-35.9 > T7.3
Phase enc. dir.	A >> P
FoV read	350 mm
FoV phase	68.8 %
Slice thickness	6.0 mm
TR	40.48 ms
Multi-slice mode	Sequential
Series	Ascending
Concatenations	1

Geometry - AutoAlign

Slice group	1
Position	L40.2 P0.7 F1.9 mm
Orientation	S > C-35.9 > T7.3
Phase enc. dir.	A >> P
AutoAlign	---
Initial Position	L40.2 P0.7 F1.9
L	40.2 mm
P	0.7 mm F
1.9 mm Initial Rotation	-
2.50 deg Initial Orientation	
S > C	
S > C	-35.9
> T	7.3

Geometry - Saturation

Wrap-up Magn.	None
Special sat.	None

Geometry - Navigator

Geometry - Tim Planning Suite

Set-n-Go Protocol	Off
Table position	H
Table position	0 mm
Inline Composing	Off

System - Miscellaneous

Positioning mode	REF
Table position	H
Table position	0 mm
MSMA	S - C - T
Sagittal	R >> L
Coronal	A >> P
Transversal	F >> H
Coil Combine Mode	Sum of Squares
Matrix Optimization	Off
Coil Focus	Flat
AutoAlign	---
Coil Select Mode	Default

System - Adjustments

B0 Shim mode	Tune up
B1 Shim mode	TrueForm
Adjust with body coil	Off
Confirm freq. adjustment	Off
Assume Dominant Fat	Off
Assume Silicone	Off
Adjustment Tolerance	Auto

System - Adjust Volume

Position	Isocenter
Orientation	Transversal
Rotation	0.00 deg
A >> P	263 mm
R >> L	350 mm
F >> H	350 mm
Reset	Off

System - pTx Volumes

B1 Shim mode	TrueForm
Excitation	Slice-sel.

System - Tx/Rx

Frequency 1H	123.255690 MHz
Correction factor	1
Gain	High
Img. Scale Cor.	1.000
Reset	Off
? Ref. amplitude 1H	0.000 V

Physio - Signal1

1st Signal/Mode	ECG/Retro
Average cycle	990 ± 10 ms
Average cycle	1000 ± 10 ms
Calculated phases	20
TR	40.48 ms
Concatenations	1
Segments	4
Arrhythmia detection	None

Physio - PACE

Resp. control	Off
Concatenations	1

Angio - Common

Flow mode	Single dir.
Encodings	1
Velocity enc.	120 cm/s
Direction	Through plane
Rephased images	On

Angio - Common

Magnitude images	On
Magnitude sum	Off
Phase images	On

Angio - Inline

Subtract	Off
Measurements	1
StdDev	Off
Save original images	On

Angio - Cardiac

Inline Evaluation	Off
TE	2.74 ms
TR	40.48 ms
Save original images	On

Angio - MIP

MIP-Sag	Off
MIP-Cor	Off
MIP-Tra	Off
MIP-Time	Off
Save original images	On

Angio - Composing

Inline Composing	Off
Distortion Corr.	On
Mode	2D
Unfiltered images	Off

Sequence - Part 1

Introduction	Off
Dimension	2D
Reordering	Linear
Asymmetric echo	Strong
Flow comp.	Yes
Optimization	Min. TE TR
Multi-slice mode	Sequential
Echo spacing	5.1 ms
Sequence type	Gre
Bandwidth	445 Hz/Px

Sequence - Part 2

Define	Segments
Segments	4
RF pulse type	Normal
Gradient mode	Normal
Excitation	Slice-sel.
Flip angle mode	Constant
RF spoiling	On
Phase Enc. Rewinder	On
Cine	On

Sequence - Assistant

Mode	Off
Allowed delay	0 s

\\Research\Abdominal\SS BEAT Kidney\iBEAT_DKDv10.6\T2star_map_pancreas_tra_mbh
TA: 1:15 PM: REF Voxel size: 1.6×1.6×5.0 mmPAT: 2 Rel. SNR: 1.00 : fl

Properties

Prio recon	Off
Load images to viewer	Off
Inline movie	Off
Auto store images	On
Load images to stamp segments	On
Load images to graphic segments	On
Auto open inline display	On
Auto close inline display	Off
Start measurement without further preparation	Off
Wait for user to start	Off
Start measurements	Single measurement

Routine

Slice group	1
Slices	12
Dist. factor	0 %
Position	L12.9 P10.5 H99.0 mm
Orientation	Transversal
Phase enc. dir.	A >> P
AutoAlign	---
Phase oversampling	0 %
FoV read	400 mm
FoV phase	100.0 %
Slice thickness	5.0 mm
TR	96.0 ms
TE 1	3.69 ms
TE 2	7.38 ms
TE 3	11.07 ms
TE 4	14.76 ms
TE 5	18.45 ms
TE 6	22.14 ms
TE 7	25.83 ms
TE 8	29.52 ms
TE 9	33.21 ms
TE 10	36.90 ms
TE 11	40.59 ms
TE 12	44.28 ms
Averages	1
Concatenations	6
Filter	Distortion Corr.(2D)
Coil elements	BO3;SP8

Contrast - Common

TR	96.0 ms
TE 1	3.69 ms
TE 2	7.38 ms
TE 3	11.07 ms
TE 4	14.76 ms
TE 5	18.45 ms
TE 6	22.14 ms
TE 7	25.83 ms
TE 8	29.52 ms
TE 9	33.21 ms
TE 10	36.90 ms
TE 11	40.59 ms
TE 12	44.28 ms
MTC	Off
Magn. preparation	None
Flip angle	25 deg
Fat suppr.	None

Contrast - Common

Water suppr.	None
SWI	Off

Contrast - Dynamic

Averages	1
Averaging mode	Long term
Reconstruction	Magn./Phase
Measurements	1
Multiple series	Off

Resolution - Common

FoV read	400 mm
FoV phase	100.0 %
Slice thickness	5.0 mm
Base resolution	256
Phase resolution	100 %
Phase partial Fourier	Off
Interpolation	Off

Resolution - iPAT

PAT mode	GRAPPA
Accel. factor PE	2
Ref. lines PE	24
Reference scan mode	GRE/separate

Resolution - Filter Image

Image Filter	Off
Distortion Corr.	On
Mode	2D
Unfiltered images	Off
Prescan Normalize	Off
Normalize	Off
B1 filter	Off

Resolution - Filter Rawdata

Raw filter	Off
Elliptical filter	Off

Geometry - Common

Slice group	1
Slices	12
Dist. factor	0 %
Position	L12.9 P10.5 H99.0 mm
Orientation	Transversal
Phase enc. dir.	A >> P
FoV read	400 mm
FoV phase	100.0 %
Slice thickness	5.0 mm
TR	96.0 ms
Multi-slice mode	Interleaved
Series	Interl. in B.-h.
Concatenations	6

Geometry - AutoAlign

Slice group	1
Position	L12.9 P10.5 H99.0 mm
Orientation	Transversal
Phase enc. dir.	A >> P
AutoAlign	---
Initial Position	L12.9 P10.5 H35.0

Geometry - AutoAlign

L	12.9 mm
P	10.5 mm
H	35.0 mm
Initial Rotation	0.00 deg
Initial Orientation	Transversal

Geometry - Saturation

Saturation mode	Standard
Fat suppr.	None
Water suppr.	None
Special sat.	None

Geometry - Tim Planning Suite

Set-n-Go Protocol	Off
Table position	H
Table position	64 mm
Inline Composing	Off

Physio - Signal1

1st Signal/Mode	None
TR	96.0 ms
Concatenations	6
Segments	1

Physio - Cardiac

Tagging	None
Magn. preparation	None
Fat suppr.	None
Dark blood	Off
FoV read	400 mm
FoV phase	100.0 %
Phase resolution	100 %

Physio - PACE

Resp. control	Breath-hold
Concatenations	6

System - Miscellaneous

Positioning mode	REF
Table position	H
Table position	64 mm
MSMA	S - C - T
Sagittal	R >> L
Coronal	A >> P
Transversal	H >> F
Coil Combine Mode	Adaptive Combine
Save uncombined	Off
Matrix Optimization	Off
Coil Focus	Flat
AutoAlign	---
Coil Select Mode	Default

System - Adjustments

B0 Shim mode	Standard
B1 Shim mode	TrueForm
Adjust with body coil	Off
Confirm freq. adjustment	Off
Assume Dominant Fat	Off
Assume Silicone	Off
Adjustment Tolerance	Auto

System - Adjust Volume

Position	L12.9 P10.5 H99.0 mm
Orientation	Transversal
Rotation	0.00 deg
A >> P	400 mm
R >> L	400 mm
F >> H	60 mm
Reset	Off

System - pTx Volumes

B1 Shim mode	TrueForm
Excitation	Slice-sel.

System - Tx/Rx

Frequency 1H	123.255690 MHz
Correction factor	1
Gain	High
Img. Scale Cor.	1.000
Reset	Off
? Ref. amplitude 1H	0.000 V

Inline - Common

Subtract	Off
Measurements	1
StdDev	Off
Liver registration	Off
Save original images	On

Inline - MIP

MIP-Sag	Off
MIP-Cor	Off
MIP-Tra	Off
MIP-Time	Off
Save original images	On

Inline - Soft Tissue

Wash - In	Off
Wash - Out	Off
TTP	Off
PEI	Off
MIP - time	Off
Measurements	1

Inline - Composing

Inline Composing	Off
Distortion Corr.	On
Mode	2D
Unfiltered images	Off

Inline - MapIt

Noise threshold	15
Save original images	On
MapIt	T2* map
Flip angle	25 deg
Measurements	1
Contrasts	12
TR	96.0 ms
TE 1	3.69 ms
TE 2	7.38 ms
TE 3	11.07 ms
TE 4	14.76 ms
TE 5	18.45 ms
TE 6	22.14 ms
TE 7	25.83 ms
TE 8	29.52 ms
TE 9	33.21 ms

Inline - MapIt

TE 10	36.90 ms
TE 11	40.59 ms
TE 12	44.28 ms

Sequence - Part 1

Introduction	Off
Dimension	2D
Phase stabilisation	Off
Asymmetric echo	Off
Contrasts	12
Flow comp. 1	No
Readout mode	Bipolar
Multi-slice mode	Interleaved
Bandwidth 1	750 Hz/Px
Bandwidth 2	750 Hz/Px
Bandwidth 3	750 Hz/Px
Bandwidth 4	750 Hz/Px
Bandwidth 5	750 Hz/Px
Bandwidth 6	750 Hz/Px
Bandwidth 7	750 Hz/Px
Bandwidth 8	750 Hz/Px
Bandwidth 9	750 Hz/Px
Bandwidth 10	750 Hz/Px
Bandwidth 11	750 Hz/Px
Bandwidth 12	750 Hz/Px

Sequence - Part 2

Segments	1
Acoustic noise reduction	None RF
pulse type	Fast
Gradient mode	Fast
Excitation	Slice-sel.
RF spoiling	On

Sequence - Assistant

Mode	Off
Allowed delay	0 s

\\Research\Abdominal\SS BEAT Kidney\iBEAT_DKDv10.6\T1w_kidneys_cor-oblique_mbh
TA: 0:16 PM: REF Voxel size: 0.8×0.8×2.5 mmPAT: 2 Rel. SNR: 1.00 : fl

Properties

Prio recon	Off
Load images to viewer	On
Inline movie	Off
Auto store images	On
Load images to stamp segments	On
Load images to graphic segments	On
Auto open inline display	On
Auto close inline display	On
Start measurement without further preparation	Off
Wait for user to start	Off
Start measurements	Single measurement

Routine

Slice group	1
Slices	20
Dist. factor	40 %
Position	L15.3 P46.1 H71.0 mm
Orientation	C > T3.4
Phase enc. dir.	R >> L
AutoAlign	---
Phase oversampling	0 %
FoV read	400 mm
FoV phase	100.0 %
Slice thickness	2.5 mm
TR	148.0 ms
TE	4.92 ms
Averages	1
Concatenations	1
Filter	Distortion Corr.(2D), Prescan Normalize
Coil elements	BO1-3;SP6-8

Contrast - Common

TR	148.0 ms
TE	4.92 ms
MTC	Off
Magn. preparation	None
Flip angle	70 deg
Fat suppr.	None
Water suppr.	None
SWI	Off

Contrast - Dynamic

Averages	1
Averaging mode	Long term
Reconstruction	Magn./Phase
Measurements	1
Multiple series	Off

Resolution - Common

FoV read	400 mm
FoV phase	100.0 %
Slice thickness	2.5 mm
Base resolution	256
Phase resolution	100 %
Phase partial Fourier	6/8
Interpolation	On

Resolution - iPAT

PAT mode	GRAPPA
Accel. factor PE	2
Ref. lines PE	24
Reference scan mode	Integrated

Resolution - Filter Image

Image Filter	Off
Distortion Corr.	On
Mode	2D
Unfiltered images	Off
Prescan Normalize	On
Unfiltered images	Off
Normalize	Off
B1 filter	Off

Resolution - Filter Rawdata

Raw filter	Off
Elliptical filter	Off

Geometry - Common

Slice group	1
Slices	20
Dist. factor	40 %
Position	L15.3 P46.1 H71.0 mm
Orientation	C > T3.4
Phase enc. dir.	R >> L
FoV read	400 mm
FoV phase	100.0 %
Slice thickness	2.5 mm
TR	148.0 ms
Multi-slice mode	Interleaved
Series	Interleaved
Concatenations	1

Geometry - AutoAlign

Slice group	1
Position	L15.3 P46.1 H71.0 mm
Orientation	C > T3.4
Phase enc. dir.	R >> L
AutoAlign	---
Initial Position	L15.3 P46.1 H7.0
L	15.3 mm
P	46.1 mm
H	7.0 mm
Initial Rotation	0.00 deg
Initial Orientation	C > T
C > T	3.4
> S	0.0

Geometry - Saturation

Saturation mode	Standard
Fat suppr.	None
Water suppr.	None
Special sat.	None

Geometry - Tim Planning Suite

Set-n-Go Protocol	Off
Table position	H
Table position	64 mm
Inline Composing	Off

System - Miscellaneous

Positioning mode	REF
Table position	H
Table position	64 mm
MSMA	S - C - T
Sagittal	R >> L
Coronal	A >> P
Transversal	H >> F
Coil Combine Mode	Adaptive Combine
Save uncombined	Off
Matrix Optimization	Off
Coil Focus	Flat
AutoAlign	---
Coil Select Mode	Default

System - Adjustments

B0 Shim mode	Standard
B1 Shim mode	TrueForm
Adjust with body coil	Off
Confirm freq. adjustment	Off
Assume Dominant Fat	Off
Assume Silicone	Off
Adjustment Tolerance	Auto

System - Adjust Volume

Position	L15.3 P46.1 H71.0 mm
Orientation	C > T3.4
Rotation	0.00 deg
R >> L	400 mm
F >> H	400 mm
A >> P	69 mm
Reset	Off

System - pTx Volumes

B1 Shim mode	TrueForm
Excitation	Slice-sel.

System - Tx/Rx

Frequency 1H	123.255690 MHz
Correction factor	1
Gain	High
Img. Scale Cor.	1.000
Reset	Off
? Ref. amplitude 1H	0.000 V

Physio - Signal1

1st Signal/Mode	None
TR	148.0 ms
Concatenations	1
Segments	1

Physio - Cardiac

Tagging	None
Magn. preparation	None
Fat suppr.	None
Dark blood	Off
FoV read	400 mm
FoV phase	100.0 %
Phase resolution	100 %

Physio - PACE

Resp. control	Breath-hold
Concatenations	1

Inline - Common

Subtract	Off
Measurements	1
StdDev	Off
Liver registration	Off
Save original images	On

Inline - MIP

MIP-Sag	Off
MIP-Cor	Off
MIP-Tra	Off
MIP-Time	Off
Save original images	On

Inline - Soft Tissue

Wash - In	Off
Wash - Out	Off
TTP	Off
PEI	Off
MIP - time	Off
Measurements	1

Inline - Composing

Inline Composing	Off
Distortion Corr.	On
Mode	2D
Unfiltered images	Off

Inline - MapIt

Save original images	On
MapIt	None
Flip angle	70 deg
Measurements	1
Contrasts	1
TR	148.0 ms
TE	4.92 ms

Sequence - Part 1

Introduction	Off
Dimension	2D
Phase stabilisation	Off
Asymmetric echo	Allowed
Contrasts	1
Flow comp.	No
Multi-slice mode	Interleaved
Bandwidth	750 Hz/Px

Sequence - Part 2

Segments	1
Acoustic noise reduction	None RF
pulse type	Fast
Gradient mode	Fast
Excitation	Slice-sel.
RF spoiling	On

Sequence - Assistant

Mode	Off
Allowed delay	60

\\Research\Abdominal\SS BEAT Kidney\iBEAT_DKDv10.6\T1map_kidneys_cor-oblique_mbh
TA: 1:22 PM: FIX Voxel size: 1.0×1.0×5.0 mmPAT: 2 Rel. SNR: 1.00 : tfl_r

Properties

Prio recon	Off
Load images to viewer	On
Inline movie	Off
Auto store images	On
Load images to stamp segments	On
Load images to graphic segments	On
Auto open inline display	Off
Auto close inline display	Off
Start measurement without further preparation	On
Wait for user to start	Off
Start measurements	Single measurement

Routine

Slice group	1
Slices	5
Dist. factor	50 %
Position	L15.3 P46.1 H71.0 mm
Orientation	C > T3.4
Phase enc. dir.	R >> L
AutoAlign	---
Phase oversampling	10 %
FoV read	400 mm
FoV phase	100.0 %
Slice thickness	5.0 mm
TR	506.63 ms
TE	2.36 ms
Averages	1
Concatenations	5
Filter	Distortion Corr.(2D)
Coil elements	BO1-3;SP6-8

Contrast - Common

TR	506.63 ms
TE	2.36 ms
Magn. preparation	Non-sel. IR T1map
TI	260 ms
Flip angle	12 deg
Fat suppr.	None
Wrap-up Magn.	None

Contrast - Dynamic

Averages	1
Averaging mode	Short term
Reconstruction	Magn./Phase
Measurements	1
Multiple series	Off

Resolution - Common

FoV read	400 mm
FoV phase	100.0 %
Slice thickness	5.0 mm
Base resolution	192
Phase resolution	100 %
Phase partial Fourier	5/8
Trajectory	Cartesian
Interpolation	On

Resolution - iPAT

PAT mode	GRAPPA

Resolution - iPAT

Accel. factor PE	2
Ref. lines PE	36
Reference scan mode	GRE/separate

Resolution - Filter Image

Image Filter	Off
Distortion Corr.	On
Mode	2D
Unfiltered images	Off
Prescan Normalize	Off
Normalize	Off
B1 filter	Off

Resolution - Filter Rawdata

Raw filter	Off
Elliptical filter	Off
POCS	Off

Geometry – Common

Slice group	1
Slices	5
Dist. factor	50 %
Position	L15.3 P46.1 H71.0 mm
Orientation	C > T3.4
Phase enc. dir.	R >> L
FoV read	400 mm
FoV phase	100.0 %
Slice thickness	5.0 mm
TR	506.63 ms
Multi-slice mode	Sequential
Series	Base To Apex
Concatenations	5

Geometry - AutoAlign

Slice group	1
Position	L15.3 P46.1 H71.0 mm
Orientation	C > T3.4
Phase enc. dir.	R >> L
AutoAlign	---
Initial Position	L15.3 P46.1 H7.0
L	15.3 mm
P	46.1 mm
H	7.0 mm
Initial Rotation	0.00 deg
Initial Orientation	C > T
C > T	3.4
> S	0.0

Geometry - Saturation

Fat suppr.	None
Wrap-up Magn.	None
Special sat.	None

Geometry - Navigator

Geometry - Tim Planning Suite

Set-n-Go Protocol	Off
Table position	H
Table position	64 mm
Inline Composing	Off

System - Miscellaneous

Positioning mode	FIX
Table position	H
Table position	64 mm
MSMA	S - C - T
Sagittal	R >> L
Coronal	A >> P
Transversal	H >> F
Coil Combine Mode	Adaptive Combine
Save uncombined	Off
Matrix Optimization	Off
Coil Focus	Flat
AutoAlign	---
Coil Select Mode	Default

System - Adjustments

B0 Shim mode	Cardiac
B1 Shim mode	TrueForm
Adjust with body coil	Off
Confirm freq. adjustment	Off
Assume Dominant Fat	Off
Assume Silicone	Off
Adjustment Tolerance	Auto

System - Adjust Volume

! Position	R3.1 A87.4 H49.9 mm
! Orientation	S > T-0.1
! Rotation	0.00 deg
! A >> P	117 mm
! F >> H	174 mm
! R >> L	220 mm
Reset	Off

System - pTx Volumes

B1 Shim mode	TrueForm
Excitation	Slice-sel.

System - Tx/Rx

Frequency 1H	123.255690 MHz
Correction factor	1
Gain	High
Img. Scale Cor.	1.000
Reset	Off
? Ref. amplitude 1H	0.000 V

Physio - Signal1

1st Signal/Mode	None
TR	506.63 ms
Concatenations	5
Segments	106

Physio - Cardiac

Tagging	None
Magn. preparation	Non-sel. IR T1map
TI	260 ms
Fat suppr.	None
Dark blood	Off
FoV read	400 mm
FoV phase	100.0%
Phase resolution	100%
Cine	Off
Trajectory	Cartesian
Dummy heartbeats	0
Motion Correction	Standard

Physio - PACE

Resp. control	Breath-hold
Concatenations	5

Inline – Common

Subtract	Off
StdDev	Off
Motion Correction	Standard
Measurements	1
Save Original images	On

Inline – Cardiac

Inline Evaluation	T1 map
Magn. Preparation	Non-selective IR T1map
Num. of Preps	3
Motion Correction	Standard
Save Original images	On
Sampling Duration 1	16 beats
Sampling Duration 2	8 beats
Sampling duration 3	4 beats
Recovery Duration 1	2 beats
Recovery duration 2	2 beats
Recovery duration 3	0 beats
Contrasts	1
TE	2.36 ms
TR	506.63 ms

Inline – MIP

MIP-Sag	Off
MIP- Cor	Off
MIP- Tra	Off
MIP-Time	Off
Save original images	On

Inline - Composing

Inline Composing	Off
Distortion Corr.	On
Mode	2D
Unfiltered images	Off

Sequence - Part 1

Introduction	Off
Dimension	2D
Reordering	Linear
Asymmetric echo	Weak
Contrasts	1
Flow comp.	Slice/Read
Optimization	Min. TE TR
Multi-slice mode	Sequential
Sequence type	Gre
Bandwidth	744 Hz/Px

Sequence - Part 2

Define	Shots
Shots per slice	1
Segments	106
RF pulse type	Normal
Gradient mode	Fast
Excitation	Slice-sel.
Flip angle mode	Constant
RF spoiling	On
Phase Enc. Rewinder	On
Cine	Off

Sequence – Assistant

Mode	Off
Allowed delay	0 s

\\Research\Abdominal\SS BEAT Kidney\iBEAT_DKDv10.6\T2map_kidneys_cor-oblique_mbh
TA: 1:12 PM: FIX Voxel size: 1.0×1.0×5.0 mmPAT: 2 Rel. SNR: 1.00 : tfl_r

Properties

Prio recon	Off
Load images to viewer	On
Inline movie	Off
Auto store images	On
Load images to stamp segments	On
Load images to graphic segments	On
Auto open inline display	Off
Auto close inline display	Off
Start measurement without further preparation	On
Wait for user to start	Off
Start measurements	Single measurement

Routine

Slice group	1
Slices	5
Dist. factor	50 %
Position	L15.3 P46.1 H71.0 mm
Orientation	C > T3.4
Phase enc. dir.	R >> L
AutoAlign	---
Phase oversampling	0 %
FoV read	400 mm
FoV phase	100.0 %
Slice thickness	5.0 mm
TR	462.83 ms
TE	2.36 ms
Averages	1
Concatenations	5
Filter	Distortion Corr.(2D)
Coil elements	BO1-3;SP6-8

Contrast - Common

TR	462.83 ms
TE	2.36 ms
Magn. preparation	T2 prep. adiab.
T2 prep. duration 1	0 ms
T2 prep. duration 2	30 ms
T2 prep. duration 3	40 ms
T2 prep. duration 4	50 ms
T2 prep. duration 5	60 ms
T2 prep. duration 6	70 ms
T2 prep. duration 7	80 ms
T2 prep. duration 8	90 ms
T2 prep. duration 9	100 ms
T2 prep. duration 10	110 ms
T2 prep. duration 11	120 ms
Flip angle	12 deg
Fat suppr.	None
Wrap-up Magn.	None

Contrast - Dynamic

Averages	1
Averaging mode	Short term
Reconstruction	Magn./Phase
Measurements	1
Multiple series	Off

Resolution - Common

FoV read	400 mm
FoV phase	100.0 %
Slice thickness	5.0 mm
Base resolution	192
Phase resolution	100 %
Phase partial Fourier	6/8
Trajectory	Cartesian
Interpolation	On

Resolution - iPAT

PAT mode	GRAPPA
Accel. factor PE	2
Ref. lines PE	36
Reference scan mode	GRE/separate

Resolution - Filter Image

Image Filter	Off
Distortion Corr.	On
Mode	2D
Unfiltered images	Off
Prescan Normalize	Off
Normalize	Off
B1 filter	Off

Resolution - Filter Rawdata

Raw filter	Off
Elliptical filter	Off
POCS	Off

Geometry - Common

Slice group	1
Slices	5
Dist. factor	50 %
Position	L15.3 P46.1 H71.0 mm
Orientation	C > T3.4
Phase enc. dir.	R >> L
FoV read	400 mm
FoV phase	100.0 %
Slice thickness	5.0 mm
TR	462.83 ms
Multi-slice mode	Sequential
Series	Interl. in B.-h.
Concatenations	5

Geometry - AutoAlign

Slice group	1
Position	L15.3 P46.1 H71.0 mm
Orientation	C > T3.4
Phase enc. dir.	R >> L
AutoAlign	---
Initial Position	L15.3 P46.1 H7.0
L	15.3 mm
P	46.1 mm
H	7.0 mm
Initial Rotation	0.00 deg
Initial Orientation	C > T
C > T	3.4
> S	0.0

Geometry - Saturation

Fat suppr.	None

Geometry - Saturation

Wrap-up Magn.	None
Special sat.	None

Geometry - Navigator

Geometry - Tim Planning Suite

Set-n-Go Protocol	Off
Table position	H
Table position	64 mm
Inline Composing	Off

System - Miscellaneous

Positioning mode	FIX
Table position	H
Table position	64 mm
MSMA	S - C - T
Sagittal	R >> L
Coronal	A >> P
Transversal	H >> F
Coil Combine Mode	Adaptive Combine
Save uncombined	Off
Matrix Optimization	Off
Coil Focus	Flat
AutoAlign	---
Coil Select Mode	Off - All

System - Adjustments

B0 Shim mode	Cardiac
B1 Shim mode	TrueForm
Adjust with body coil	Off
Confirm freq. adjustment	Off
Assume Dominant Fat	Off
Assume Silicone	Off
Adjustment Tolerance	Auto

System - Adjust Volume

! Position	R3.1 A87.4 H56.9 mm
! Orientation	S > T-0.1
! Rotation	0.00 deg
! A >> P	117 mm
! F >> H	174 mm
! R >> L	220 mm
Reset	Off

System - pTx Volumes

B1 Shim mode	TrueForm
Excitation	Slice-sel.

System - Tx/Rx

Frequency 1H	123.255690 MHz
Correction factor	1
Gain	High
Img. Scale Cor.	1.000
Reset	Off
? Ref. amplitude 1H	0.000 V

Physio - Signal 1

1st Signal/Mode	None
TR	462.83 ms
Concatenations	5
Segments	96

Physio - Cardiac

Tagging	None
Magn. preparation	T2 prep. adiab.
T2 prep. duration 1	0 ms
T2 prep. duration 2	30 ms
T2 prep. duration 3	40 ms
T2 prep. duration 4	50 ms
T2 prep. duration 5	60 ms
T2 prep. duration 6	70 ms
T2 prep. duration 7	80 ms
T2 prep. duration 8	90 ms
T2 prep. duration 9	100 ms
T2 prep. duration 10	110 ms
T2 prep. duration 11	120 ms
Fat suppr.	None
Dark blood	Off
FoV read	400 mm
FoV phase	100.0 %
Phase resolution	100 %
Cine	Off
Trajectory	Cartesian
Dummy heartbeats	0
Motion Correction	Standard

Physio - PACE

Resp. control	Breath-hold
Concatenations	5

Inline - Common

Subtract	Off
StdDev	Off
Motion Correction	Standard
Measurements	1
Save Original images	On

Inline - Cardiac

Inline Evaluation	T2 map
Magn. Preparation	T2 Prep
Num. of Preps	11
Motion Correction	Standard
Save Original images	On
T2 Prep. Duration 1	0 ms
T2 Prep. Duration 2	30 ms
T2 Prep. Duration 3	40 ms
T2 Prep. Duration 4	50 ms
T2 Prep. Duration 5	60 ms
T2 Prep. Duration 6	70 ms
T2 Prep. Duration 7	80 ms
T2 Prep. Duration 8	90 ms
T2 Prep. Duration 9	100 ms
T2 Prep. Duration 10	110 ms
T2 Prep. Duration 11	120 ms
Recovery duration 1	2 beats
Contrasts	1
TE	2.36 ms
TR	462.83 ms

Inline - MIP

MIP-Sag	Off
MIP- Cor	Off
MIP- Tra	Off
MIP-Time	Off
Save original images	On

Inline - Composing

Inline Composing	Off
Distortion Corr.	On
Mode	2D
Unfiltered images	Off

Sequence - Part 1

Introduction	Off
Dimension	2D
Reordering	Linear
Asymmetric echo	Weak
Contrasts	1
Flow comp.	Slice/Read
Optimization	None
Multi-slice mode	Sequential
Sequence type	Gre
Bandwidth	744 Hz/Px

Sequence - Part 2

Define	Shots
Shots per slice	1
Segments	96
RF pulse type	Normal
Gradient mode	Fast
Excitation	Slice-sel.
Flip angle mode	Constant
RF spoiling	On
Phase Enc. Rewinder	On
Cine	Off

Sequence - Assistant

Mode	Off
Allowed delay	0 s

\\Research\Abdominal\SS BEAT Kidney\iBEAT_DKDv10.6\T2star_map_kidneys_cor-oblique_mbh
TA: 0:37 PM: FIX Voxel size: 0.8×0.8×5.0 mmPAT: 2 Rel. SNR: 1.00 : fl

Properties

Prio recon	Off
Load images to viewer	Off
Inline movie	Off
Auto store images	On
Load images to stamp segments	On
Load images to graphic segments	On
Auto open inline display	On
Auto close inline display	Off
Start measurement without further preparation	On
Wait for user to start	Off
Start measurements	Single measurement

Routine

Slice group	1
Slices	5
Dist. factor	50 %
Position	L15.3 P46.1 H71.0 mm
Orientation	C > T3.4
Phase enc. dir.	R >> L
AutoAlign	---
Phase oversampling	0 %
FoV read	400 mm
FoV phase	100.0 %
Slice thickness	5.0 mm
TR	96.0 ms
TE 1	3.69 ms
TE 2	7.38 ms
TE 3	11.07 ms
TE 4	14.76 ms
TE 5	18.45 ms
TE 6	22.14 ms
TE 7	25.83 ms
TE 8	29.52 ms
TE 9	33.21 ms
TE 10	36.90 ms
TE 11	40.59 ms
TE 12	44.28 ms
Averages	1
Concatenations	3
Filter	Distortion Corr.(2D)
Coil elements	BO1-3;SP6-8

Contrast - Common

TR	96.0 ms
TE 1	3.69 ms
TE 2	7.38 ms
TE 3	11.07 ms
TE 4	14.76 ms
TE 5	18.45 ms
TE 6	22.14 ms
TE 7	25.83 ms
TE 8	29.52 ms
TE 9	33.21 ms
TE 10	36.90 ms
TE 11	40.59 ms
TE 12	44.28 ms
MTC	Off
Magn. preparation	None
Flip angle	25 deg
Fat suppr.	None

Contrast - Common

Water suppr.	None
SWI	Off

Contrast - Dynamic

Averages	1
Averaging mode	Long term
Reconstruction	Magn./Phase
Measurements	1
Multiple series	Off

Resolution - Common

FoV read	400 mm
FoV phase	100.0 %
Slice thickness	5.0 mm
Base resolution	256
Phase resolution	100 %
Phase partial Fourier	Off
Interpolation	On

Resolution - iPAT

PAT mode	GRAPPA
Accel. factor PE	2
Ref. lines PE	24
Reference scan mode	GRE/separate

Resolution - Filter Image

Image Filter	Off
Distortion Corr.	On
Mode	2D
Unfiltered images	Off
Prescan Normalize	Off
Normalize	Off
B1 filter	Off

Resolution - Filter Rawdata

Raw filter	Off
Elliptical filter	Off

Geometry - Common

Slice group	1
Slices	5
Dist. factor	50 %
Position	L15.3 P46.1 H71.0 mm
Orientation	C > T3.4
Phase enc. dir.	R >> L
FoV read	400 mm
FoV phase	100.0 %
Slice thickness	5.0 mm
TR	96.0 ms
Multi-slice mode	Interleaved
Series	Interl. in B.-h.
Concatenations	3

Geometry - AutoAlign

Slice group	1
Position	L15.3 P46.1 H71.0 mm
Orientation	C > T3.4
Phase enc. dir.	R >> L
AutoAlign	---
Initial Position	L15.3 P46.1 H7.0

Geometry - AutoAlign

L	15.3 mm
P	46.1 mm
H	7.0 mm
Initial Rotation	0.00 deg
Initial Orientation	C > T
C > T	3.4
> S	0.0

Geometry - Saturation

Saturation mode	Standard
Fat suppr.	None
Water suppr.	None
Special sat.	None

Geometry - Tim Planning Suite

Set-n-Go Protocol	Off
Table position	H
Table position	64 mm
Inline Composing	Off

System - Miscellaneous

Positioning mode	FIX
Table position	H
Table position	64 mm
MSMA	S - C - T
Sagittal	R >> L
Coronal	A >> P
Transversal	H >> F
Coil Combine Mode	Adaptive Combine
Save uncombined	Off
Matrix Optimization	Off
Coil Focus	Flat
AutoAlign	---
Coil Select Mode	Default

System - Adjustments

B0 Shim mode	Standard
B1 Shim mode	TrueForm
Adjust with body coil	Off
Confirm freq. adjustment	Off
Assume Dominant Fat	Off
Assume Silicone	Off
Adjustment Tolerance	Auto

System - Adjust Volume

Position	L15.3 P46.1 H71.0 mm
Orientation	C > T3.4
Rotation	0.00 deg
R >> L	400 mm
F >> H	400 mm
A >> P	35 mm
Reset	Off

System - pTx Volumes

B1 Shim mode	TrueForm
Excitation	Slice-sel.

System - Tx/Rx

Frequency 1H	123.255690 MHz
Correction factor	1
Gain	High
Img. Scale Cor.	1.000
Reset	Off

System - Tx/Rx

? Ref. amplitude 1H	0.000 V

Physio - Signal1

1st Signal/Mode	None
TR	96.0 ms
Concatenations	3
Segments	1

Physio - Cardiac

Tagging	None
Magn. preparation	None
Fat suppr.	None
Dark blood	Off
FoV read	400 mm
FoV phase	100.0 %
Phase resolution	100 %

Physio - PACE

Resp. control	Breath-hold
Concatenations	3

Inline - Common

Subtract	Off
Measurements	1
StdDev	Off
Liver registration	Off
Save original images	On

Inline - MIP

MIP-Sag	Off
MIP-Cor	Off
MIP-Tra	Off
MIP-Time	Off
Save original images	On

Inline - Soft Tissue

Wash - In	Off
Wash - Out	Off
TTP	Off
PEI	Off
MIP - time	Off
Measurements	1

Inline - Composing

Inline Composing	Off
Distortion Corr.	On
Mode	2D
Unfiltered images	Off

Inline - MapIt

Noise threshold	15
Save original images	On
MapIt	T2* map
Flip angle	25 deg
Measurements	1
Contrasts	12
TR	96.0 ms
TE 1	3.69 ms
TE 2	7.38 ms
TE 3	11.07 ms
TE 4	14.76 ms
TE 5	18.45 ms
TE 6	22.14 ms

Inline - MapIt

TE 7	25.83 ms
TE 8	29.52 ms
TE 9	33.21 ms
TE 10	36.90 ms
TE 11	40.59 ms
TE 12	44.28 ms

Sequence - Part 1

Introduction	Off
Dimension	2D
Phase stabilisation	Off
Asymmetric echo	Off
Contrasts	12
Flow comp. 1	No
Readout mode	Bipolar
Multi-slice mode	Interleaved
Bandwidth 1	750 Hz/Px
Bandwidth 2	750 Hz/Px
Bandwidth 3	750 Hz/Px
Bandwidth 4	750 Hz/Px
Bandwidth 5	750 Hz/Px
Bandwidth 6	750 Hz/Px
Bandwidth 7	750 Hz/Px
Bandwidth 8	750 Hz/Px
Bandwidth 9	750 Hz/Px
Bandwidth 10	750 Hz/Px
Bandwidth 11	750 Hz/Px
Bandwidth 12	750 Hz/Px

Sequence - Part 2

Segments	1
Acoustic noise reduction	None RF
pulse type	Fast
Gradient mode	Fast
Excitation	Slice-sel.
RF spoiling	On

Sequence - Assistant

Mode	Off
Allowed delay	0 s

\\Research\Abdominal\SS BEAT Kidney\iBEAT_DKDv10.6\IVIM_kidneys_cor-oblique_fb
TA: 2:55 PM: FIX Voxel size: 2.3×2.3×2.3 mmPAT: 2 Rel. SNR: 1.00 : epse

Properties

Prio recon	Off
Load images to viewer	Off
Inline movie	Off
Auto store images	On
Load images to stamp segments	On
Load images to graphic segments	On
Auto open inline display	Off
Auto close inline display	Off
Start measurement without further preparation	On
Wait for user to start	Off
Start measurements	Single measurement

Routine

Slice group	1
Slices	30
Dist. factor	0 %
Position	L15.3 P46.1 H71.0 mm
Orientation	C > T3.4
Phase enc. dir.	R >> L
AutoAlign	---
Phase oversampling	0 %
FoV read	400 mm
FoV phase	100.0 %
Slice thickness	2.3 mm
TR	5100 ms
TE	70.0 ms
Averages	1
Concatenations	1
Filter	Raw filter, Dynamic Field Corr., Prescan Normalize
Coil elements	BO1-3;SP6-8

Contrast - Common

TR	5100 ms
TE	70.0 ms
MTC	Off
Magn. preparation	None
Fat suppr.	SPAIR
Fat sat. mode	Strong

Contrast - Dynamic

Averages	1
Averaging mode	Long term
Reconstruction	Magnitude
Measurements	1
Delay in TR	0 ms
Multiple series	Off

Resolution - Common

FoV read	400 mm
FoV phase	100.0 %
Slice thickness	2.3 mm
Base resolution	172
Phase resolution	100 %
Phase partial Fourier	Off
Interpolation	Off

Resolution - iPAT

Accel. mode	GRAPPA
Accel. factor PE	2
Ref. lines PE	32
Reference scan mode	GRE/separate

Resolution - Filter Image

Distortion Corr.	Off
Prescan Normalize	On
Dynamic Field Corr.	On
Unfiltered images	Off

Resolution - Filter Rawdata

Raw filter	On
Elliptical filter	Off

Geometry - Common

Slice group	1
Slices	30
Dist. factor	0 %
Position	L15.3 P46.1 H71.0 mm
Orientation	C > T3.4
Phase enc. dir.	R >> L
FoV read	400 mm
FoV phase	100.0 %
Slice thickness	2.3 mm
TR	5100 ms
Multi-slice mode	Interleaved
Series	Interleaved
Concatenations	1

Geometry - AutoAlign

Slice group	1
Position	L15.3 P46.1 H71.0 mm
Orientation	C > T3.4
Phase enc. dir.	R >> L
AutoAlign	---
Initial Position	L15.3 P46.1 H7.0
L	15.3 mm
P	46.1 mm
H	7.0 mm
Initial Rotation	0.00 deg
Initial Orientation	C > T
C > T	3.4
> S	0.0

Geometry - Saturation

Fat suppr.	SPAIR
Fat sat. mode	Strong
Special sat.	None

Geometry - Navigator

Geometry - Tim Planning Suite

Set-n-Go Protocol	Off
Table position	H
Table position	64 mm
Inline Composing	Off

System - Miscellaneous

Positioning mode	FIX
Table position	H
Table position	64 mm
MSMA	S - C - T
Sagittal	R >> L
Coronal	A >> P
Transversal	H >> F
Coil Combine Mode	Adaptive Combine
Matrix Optimization	Off
Coil Focus	Flat
AutoAlign	---
Coil Select Mode	Default

System - Adjustments

B0 Shim mode	Standard
B1 Shim mode	TrueForm C
Adjust with body coil	Off
Confirm freq. adjustment	Off
Assume Dominant Fat	Off
Assume Silicone	Off
Adjustment Tolerance	Auto

System - Adjust Volume

Position	L15.3 P46.1 H71.0 mm
Orientation	C > T3.4
Rotation	0.00 deg
R >> L	400 mm
F >> H	400 mm
A >> P	69 mm
Reset	Off

System - pTx Volumes

B1 Shim mode	TrueForm C
Excitation	Standard

System - Tx/Rx

Frequency 1H	123.255690 MHz
Correction factor	1
Gain	High
Img. Scale Cor.	3.000
Reset	Off
? Ref. amplitude 1H	0.000 V

Physio - Signal1

1st Signal/Mode	None
TR	5100 ms
Concatenations	1

Physio - PACE

Resp. control	Off
Concatenations	1

Diff - Neuro

Diffusion mode	Free
Diff. directions	30
Diffusion Scheme	Monopolar
Diff. weightings	1
b-value	600 s/mm²
b-value	1
Diff. weighted images	On
Trace weighted images	Off
ADC maps	Off
FA maps	Off

Diff - Neuro

Mosaic	Off
Tensor	Off
Noise level	10

Diff - Body

Diffusion mode	Free
Diff. directions	30
Diffusion Scheme	Monopolar
Diff. weightings	1
b-value	600 s/mm²
b-value	1
Diff. weighted images	On
Trace weighted images	Off
ADC maps	Off
Exponential ADC Maps	Off
FA maps	Off
Invert Gray Scale	Off
Calculated Image	Off
b-Value >=	0 s/mm²
Noise level	10

Diff - Composing

Inline Composing	Off
Distortion Corr.	Off

Sequence - Part 1

Introduction	On
Optimization	Min. TE
Multi-slice mode	Interleaved
Free echo spacing	Off
Echo spacing	0.55 ms
Bandwidth	2076 Hz/Px

Sequence - Part 2

EPI factor	172
RF pulse type	Normal
Gradient mode	Performance
Excitation	Standard

Sequence - pTX Pulses

\\Research\Abdominal\SS BEAT Kidney\iBEAT_DKDv10.6\DTI_kidneys_cor-oblique_fb
TA: 12:47 PM: FIX Voxel size: 2.3×2.3×2.3 mmPAT: 2 Rel. SNR: 1.00 : epse

Properties

Prio recon	Off
Load images to viewer	Off
Inline movie	Off
Auto store images	On
Load images to stamp segments	On
Load images to graphic segments	On
Auto open inline display	Off
Auto close inline display	Off
Start measurement without further preparation	On
Wait for user to start	Off
Start measurements	Single measurement

Routine

Slice group	1
Slices	30
Dist. factor	0 %
Position	L15.3 P46.1 H71.0 mm
Orientation	C > T3.4
Phase enc. dir.	R >> L
AutoAlign	---
Phase oversampling	0 %
FoV read	400 mm
FoV phase	100.0 %
Slice thickness	2.3 mm
TR	5100 ms
TE	70.0 ms
Averages	1
Concatenations	1
Filter	Raw filter, Dynamic Field Corr., Prescan Normalize
Coil elements	BO1-3;SP6-8

Contrast - Common

TR	5100 ms
TE	70.0 ms
MTC	Off
Magn. preparation	None
Fat suppr.	SPAIR
Fat sat. mode	Strong

Contrast - Dynamic

Averages	1
Averaging mode	Long term
Reconstruction	Magnitude
Measurements	1
Delay in TR	0 ms
Multiple series	Off

Resolution - Common

FoV read	400 mm
FoV phase	100.0 %
Slice thickness	2.3 mm
Base resolution	172
Phase resolution	100 %
Phase partial Fourier	Off
Interpolation	Off

Resolution - iPAT

Accel. mode	GRAPPA
Accel. factor PE	2
Ref. lines PE	32
Reference scan mode	GRE/separate

Resolution - Filter Image

Distortion Corr.	Off
Prescan Normalize	On
Dynamic Field Corr.	On
Unfiltered images	Off

Resolution - Filter Rawdata

Raw filter	On
Elliptical filter	Off

Geometry - Common

Slice group	1
Slices	30
Dist. factor	0 %
Position	L15.3 P46.1 H71.0 mm
Orientation	C > T3.4
Phase enc. dir.	R >> L
FoV read	400 mm
FoV phase	100.0 %
Slice thickness	2.3 mm
TR	5100 ms
Multi-slice mode	Interleaved
Series	Interleaved
Concatenations	1

Geometry - AutoAlign

Slice group	1
Position	L15.3 P46.1 H71.0 mm
Orientation	C > T3.4
Phase enc. dir.	R >> L
AutoAlign	---
Initial Position	L15.3 P46.1 H7.0
L	15.3 mm
P	46.1 mm
H	7.0 mm
Initial Rotation	0.00 deg
Initial Orientation	C > T
C > T	3.4
> S	0.0

Geometry - Saturation

Fat suppr.	SPAIR
Fat sat. mode	Strong
Special sat.	None

Geometry - Navigator

Geometry - Tim Planning Suite

Set-n-Go Protocol	Off
Table position	H
Table position	64 mm
Inline Composing	Off

System - Miscellaneous

Positioning mode	FIX
Table position	H
Table position	64 mm
MSMA	S - C - T
Sagittal	R >> L
Coronal	A >> P
Transversal	H >> F
Coil Combine Mode	Adaptive Combine
Matrix Optimization	Off
Coil Focus	Flat
AutoAlign	---
Coil Select Mode	Default

System - Adjustments

B0 Shim mode	Standard
B1 Shim mode	TrueForm C
Adjust with body coil	Off
Confirm freq. adjustment	Off
Assume Dominant Fat	Off
Assume Silicone	Off
Adjustment Tolerance	Auto

System - Adjust Volume

Position	L15.3 P46.1 H71.0 mm
Orientation	C > T3.4
Rotation	0.00 deg
R >> L	400 mm
F >> H	400 mm
A >> P	69 mm
Reset	Off

System - pTx Volumes

B1 Shim mode	TrueForm C
Excitation	Standard

System - Tx/Rx

Frequency 1H	123.255690 MHz
Correction factor	1
Gain	High
Img. Scale Cor.	3.000
Reset	Off
? Ref. amplitude 1H	0.000 V

Physio - Signal1

1st Signal/Mode	None
TR	5100 ms
Concatenations	1

Physio - PACE

Resp. control	Off
Concatenations	1

Diff - Neuro

Diffusion mode	Free
Diff. directions	146
Diffusion Scheme	Monopolar
Diff. weightings	1
b-value	600 s/mm²
b-value	1
Diff. weighted images	On
Trace weighted images	Off
ADC maps	Off
FA maps	Off

Diff - Neuro

Mosaic	Off
Tensor	Off
Noise level	10

Diff - Body

Diffusion mode	Free
Diff. directions	146
Diffusion Scheme	Monopolar
Diff. weightings	1
b-value	600 s/mm²
b-value	1
Diff. weighted images	On
Trace weighted images	Off
ADC maps	Off
Exponential ADC Maps	Off
FA maps	Off
Invert Gray Scale	Off
Calculated Image	Off
b-Value >=	0 s/mm²
Noise level	10

Diff - Composing

Inline Composing	Off
Distortion Corr.	Off

Sequence - Part 1

Introduction	On
Optimization	Min. TE
Multi-slice mode	Interleaved
Free echo spacing	Off
Echo spacing	0.55 ms
Bandwidth	2076 Hz/Px

Sequence - Part 2

EPI factor	172
RF pulse type	Normal
Gradient mode	Performance
Excitation	Standard

Sequence - pTX Pulses

\\Research\Abdominal\SS BEAT Kidney\iBEAT_DKDv10.6\MT_OFF_kidneys_cor-oblique_bh
TA: 0:17 PM: FIX Voxel size: 1.6×1.6×3.0 mmPAT: 3 Rel. SNR: 1.00 : fl

Properties

Prio recon	Off
Load images to viewer	On
Inline movie	Off
Auto store images	On
Load images to stamp segments	On
Load images to graphic segments	On
Auto open inline display	Off
Auto close inline display	Off
Start measurement without further preparation	On
Wait for user to start	Off
Start measurements	Single measurement

Routine

Slab group	1
Slabs	1
Dist. factor	0 %
Position	L15.3 P46.1 H71.0 mm
Orientation	C > T3.4
Phase enc. dir.	R >> L
AutoAlign	---
Phase oversampling	0 %
Slice oversampling	0.0 %
Slices per slab	16
FoV read	400 mm
FoV phase	100.0 %
Slice thickness	3.00 mm
TR	34.0 ms
TE	6.15 ms
Averages	1
Concatenations	1
Filter	Distortion Corr.(2D)
Coil elements	BO1-3;SP6-8

Contrast - Common

TR	34.0 ms
TE	6.15 ms
MTC	Off
Magn. preparation	None
Flip angle	10 deg
Fat suppr.	None
Water suppr.	None
SWI	Off

Contrast - Dynamic

Averages	1
Averaging mode	Short term
Reconstruction	Magnitude
Measurements	1
Multiple series	Each measurement

Resolution - Common

FoV read	400 mm
FoV phase	100.0 %
Slice thickness	3.00 mm
Base resolution	128
Phase resolution	80 %
Slice resolution	100 %
Phase partial Fourier	6/8
Slice partial Fourier	6/8

Resolution - Common

Interpolation	On

Resolution - iPAT

PAT mode	GRAPPA
Accel. factor PE	3
Ref. lines PE	24
Accel. factor 3D	1
Reference scan mode	Integrated

Resolution - Filter Image

Image Filter	Off
Distortion Corr.	On
Mode	2D
Unfiltered images	Off
Prescan Normalize	Off
Normalize	Off
B1 filter	Off

Resolution - Filter Rawdata

Raw filter	Off
Elliptical filter	Off

Geometry - Common

Slab group	1
Slabs	1
Dist. factor	0 %
Position	L15.3 P46.1 H71.0 mm
Orientation	C > T3.4
Phase enc. dir.	R >> L
Slice oversampling	0.0 %
Slices per slab	16
FoV read	400 mm
FoV phase	100.0 %
Slice thickness	3.00 mm
TR	34.0 ms
Multi-slice mode	Interleaved
Series	Interl. in B.-h.
Concatenations	1

Geometry - AutoAlign

Slab group	1
Position	L15.3 P46.1 H71.0 mm
Orientation	C > T3.4
Phase enc. dir.	R >> L
AutoAlign	---
Initial Position	L15.3 P46.1 H7.0
L	15.3 mm
P	46.1 mm
H	7.0 mm
Initial Rotation	0.00 deg
Initial Orientation	C > T
C > T	3.4
> S	0.0

Geometry - Saturation

Saturation mode	Standard
Fat suppr.	None
Water suppr.	None
Special sat.	None

Geometry - Tim Planning Suite

Set-n-Go Protocol	Off
Table position	H
Table position	64 mm
Inline Composing	Off

System - Miscellaneous

Positioning mode	FIX
Table position	H
Table position	64 mm
MSMA	S - C - T
Sagittal	R >> L
Coronal	A >> P
Transversal	H >> F
Coil Combine Mode	Sum of Squares
Save uncombined	Off
Matrix Optimization	Off
Coil Focus	Flat
AutoAlign	---
Coil Select Mode	Off - AutoCoilSelect

System - Adjustments

B0 Shim mode	Standard
B1 Shim mode	TrueForm
Adjust with body coil	Off
Confirm freq. adjustment	Off
Assume Dominant Fat	Off
Assume Silicone	Off
Adjustment Tolerance	Auto

System - Adjust Volume

Position	L15.3 P46.1 H71.0 mm
Orientation	C > T3.4
Rotation	0.00 deg
R >> L	400 mm
F >> H	400 mm
A >> P	48 mm
Reset	Off

System - pTx Volumes

B1 Shim mode	TrueForm
Excitation	Slab-sel.

System - Tx/Rx

Frequency 1H	123.255690 MHz
Correction factor	1
Gain	Low
Img. Scale Cor.	1.000
Reset	Off
? Ref. amplitude 1H	0.000 V

Physio - Signal1

1st Signal/Mode	None
TR	34.0 ms
Concatenations	1
Segments	1

Physio - Cardiac

Tagging	None
Magn. preparation	None
Fat suppr.	None
Dark blood	Off
FoV read	400 mm
FoV phase	100.0 %

Physio - Cardiac

Phase resolution	80 %

Physio - PACE

Resp. control	Breath-hold
Concatenations	1

Inline - Common

Subtract	Off
Measurements	1
StdDev	Off
Liver registration	Off
Save original images	On

Inline - MIP

MIP-Sag	Off
MIP-Cor	Off
MIP-Tra	Off
MIP-Time	Off
Save original images	On

Inline - Soft Tissue

Wash - In	Off
Wash - Out	Off
TTP	Off
PEI	Off
MIP - time	Off
Measurements	1

Inline - Composing

Inline Composing	Off
Distortion Corr.	On
Mode	2D
Unfiltered images	Off

Inline - MapIt

Save original images	On
MapIt	None
Flip angle	10 deg
Measurements	1
Contrasts	1
TR	34.0 ms
TE	6.15 ms

Sequence - Part 1

Introduction	Off
Dimension	3D
Elliptical scanning	On
Phase stabilisation	Off
Asymmetric echo	Off
Contrasts	1
Flow comp.	No
Multi-slice mode	Interleaved
Bandwidth	740 Hz/Px

Sequence - Part 2

Segments	1
Acoustic noise reduction	None
RF pulse type	Normal
Gradient mode	Fast
Excitation	Slab-sel.
RF spoiling	On

Sequence - Assistant

Mode	Off
Allowed delay	30 s

\\Research\Abdominal\SS BEAT Kidney\iBEAT_DKDv10.6\MT_ON_kidneys_cor-oblique_bh
TA: 0:17 PM: FIX Voxel size: 1.6×1.6×3.0 mmPAT: 3 Rel. SNR: 1.00 : fl

Properties

Prio recon	Off
Load images to viewer	On
Inline movie	Off
Auto store images	On
Load images to stamp segments	On
Load images to graphic segments	On
Auto open inline display	Off
Auto close inline display	Off
Start measurement without further preparation	On
Wait for user to start	Off
Start measurements	Single measurement

Routine

Slab group	1
Slabs	1
Dist. factor	0 %
Position	L15.3 P46.1 H71.0 mm
Orientation	C > T3.4
Phase enc. dir.	R >> L
AutoAlign	---
Phase oversampling	0 %
Slice oversampling	0.0 %
Slices per slab	16
FoV read	400 mm
FoV phase	100.0 %
Slice thickness	3.00 mm
TR	34.0 ms
TE	6.15 ms
Averages	1
Concatenations	1
Filter	Distortion Corr.(2D)
Coil elements	BO1-3;SP6-8

Contrast - Common

TR	34.0 ms
TE	6.15 ms
MTC	On
Magn. preparation	None
Flip angle	10 deg
Fat suppr.	None
Water suppr.	None
SWI	Off

Contrast - Dynamic

Averages	1
Averaging mode	Short term
Reconstruction	Magnitude
Measurements	1
Multiple series	Each measurement

Resolution - Common

FoV read	400 mm
FoV phase	100.0 %
Slice thickness	3.00 mm
Base resolution	128
Phase resolution	80 %
Slice resolution	100 %
Phase partial Fourier	6/8
Slice partial Fourier	6/8

Resolution - Common

Interpolation	On

Resolution - iPAT

PAT mode	GRAPPA
Accel. factor PE	3
Ref. lines PE	24
Accel. factor 3D	1
Reference scan mode	Integrated

Resolution - Filter Image

Image Filter	Off
Distortion Corr.	On
Mode	2D
Unfiltered images	Off
Prescan Normalize	Off
Normalize	Off
B1 filter	Off

Resolution - Filter Rawdata

Raw filter	Off
Elliptical filter	Off

Geometry - Common

Slab group	1
Slabs	1
Dist. factor	0 %
Position	L15.3 P46.1 H71.0 mm
Orientation	C > T3.4
Phase enc. dir.	R >> L
Slice oversampling	0.0 %
Slices per slab	16
FoV read	400 mm
FoV phase	100.0 %
Slice thickness	3.00 mm
TR	34.0 ms
Multi-slice mode	Interleaved
Series	Interl. in B.-h.
Concatenations	1

Geometry - AutoAlign

Slab group	1
Position	L15.3 P46.1 H71.0 mm
Orientation	C > T3.4
Phase enc. dir.	R >> L
AutoAlign	---
Initial Position	L15.3 P46.1 H7.0
L	15.3 mm
P	46.1 mm
H	7.0 mm
Initial Rotation	0.00 deg
Initial Orientation	C > T
C > T	3.4
> S	0.0

Geometry - Saturation

Saturation mode	Standard
Fat suppr.	None
Water suppr.	None
Special sat.	None

Geometry - Tim Planning Suite

Set-n-Go Protocol	Off
Table position	H
Table position	64 mm
Inline Composing	Off

System - Miscellaneous

Positioning mode	FIX
Table position	H
Table position	64 mm
MSMA	S - C - T
Sagittal	R >> L
Coronal	A >> P
Transversal	H >> F
Coil Combine Mode	Sum of Squares
Save uncombined	Off
Matrix Optimization	Off
Coil Focus	Flat
AutoAlign	---
Coil Select Mode	Off - AutoCoilSelect

System - Adjustments

B0 Shim mode	Standard
B1 Shim mode	TrueForm
Adjust with body coil	Off
Confirm freq. adjustment	Off
Assume Dominant Fat	Off
Assume Silicone	Off
Adjustment Tolerance	Auto

System - Adjust Volume

Position	L15.3 P46.1 H71.0 mm
Orientation	C > T3.4
Rotation	0.00 deg
R >> L	400 mm
F >> H	400 mm
A >> P	48 mm
Reset	Off

System - pTx Volumes

B1 Shim mode	TrueForm
Excitation	Slab-sel.

System - Tx/Rx

Frequency 1H	123.255690 MHz
Correction factor	1
Gain	Low
Img. Scale Cor.	1.000
Reset	Off
? Ref. amplitude 1H	0.000 V

Physio - Signal1

1st Signal/Mode	None
TR	34.0 ms
Concatenations	1
Segments	1

Physio - Cardiac

Tagging	None
Magn. preparation	None
Fat suppr.	None
Dark blood	Off
FoV read	400 mm
FoV phase	100.0 %

Physio - Cardiac

Phase resolution	80 %

Physio - PACE

Resp. control	Breath-hold
Concatenations	1

Inline - Common

Subtract	Off
Measurements	1
StdDev	Off
Liver registration	Off
Save original images	On

Inline - MIP

MIP-Sag	Off
MIP-Cor	Off
MIP-Tra	Off
MIP-Time	Off
Save original images	On

Inline - Soft Tissue

Wash - In	Off
Wash - Out	Off
TTP	Off
PEI	Off
MIP - time	Off
Measurements	1

Inline - Composing

Inline Composing	Off
Distortion Corr.	On
Mode	2D
Unfiltered images	Off

Inline - MapIt

Save original images	On
MapIt	None
Flip angle	10 deg
Measurements	1
Contrasts	1
TR	34.0 ms
TE	6.15 ms

Sequence - Part 1

Introduction	Off
Dimension	3D
Elliptical scanning	On
Phase stabilisation	Off
Asymmetric echo	Off
Contrasts	1
Flow comp.	No
Multi-slice mode	Interleaved
Bandwidth	740 Hz/Px

Sequence - Part 2

Segments	1
Acoustic noise reduction	None
RF pulse type	Normal
Gradient mode	Fast
Excitation	Slab-sel.
RF spoiling	On

Sequence - Assistant

Mode	Off
Allowed delay	30 s

\\Research\Abdominal\SS BEAT Kidney\iBEAT_DKDv10.6\ASL_planning_bh
TA: 0:17 PM: REF Voxel size: 2.0×2.0×8.0 mmPAT: Off Rel. SNR: 1.00 : tfi

Properties

Prio recon	Off
Load images to viewer	On
Inline movie	Off
Auto store images	On
Load images to stamp segments	On
Load images to graphic segments	On
Auto open inline display	Off
Auto close inline display	Off
Start measurement without further preparation	Off
Wait for user to start	Off
Start measurements	Single measurement

Routine

Slice group	1
Slices	21
Dist. factor	40 %
Position	L19.8 P3.9 H0.5 mm
Orientation	Sagittal
Phase enc. dir.	A >> P
AutoAlign	---
Phase oversampling	0 %
FoV read	380 mm
FoV phase	80.2 %
Slice thickness	8.0 mm
TR	802.07 ms
TE	1.05 ms
Averages	1
Concatenations	21
Filter	Distortion Corr.(2D), Prescan Normalize
Coil elements	BO1-3;SP6-8

Contrast - Common

TR	802.07 ms
TE	1.05 ms
TD	0 ms
Magn. preparation	None
Flip angle	40 deg
Fat suppr.	None
Wrap-up Magn.	Restore

Contrast - Dynamic

Averages	1
Averaging mode	Short term
Reconstruction	Magnitude
Measurements	1
Multiple series	Each measurement

Resolution - Common

FoV read	380 mm
FoV phase	80.2 %
Slice thickness	8.0 mm
Base resolution	192
Phase resolution	100 %
Phase partial Fourier	Off
Trajectory	Cartesian
Interpolation	Off

Resolution - iPAT

PAT mode	None

Resolution - Filter Image

Image Filter	Off
Distortion Corr.	On
Mode	2D
Unfiltered images	Off
Prescan Normalize	On
Unfiltered images	Off
Normalize	Off
B1 filter	Off

Resolution - Filter Rawdata

Raw filter	Off
Elliptical filter	Off
POCS	Off

Geometry - Common

Slice group	1
Slices	21
Dist. factor	40 %
Position	L19.8 P3.9 H0.5 mm
Orientation	Sagittal
Phase enc. dir.	A >> P
FoV read	380 mm
FoV phase	80.2 %
Slice thickness	8.0 mm
TR	802.07 ms
Multi-slice mode	Sequential
Series	Interleaved
Concatenations	21

Geometry - AutoAlign

Slice group	1
Position	L19.8 P3.9 H0.5 mm
Orientation	Sagittal
Phase enc. dir.	A >> P
AutoAlign	---
Initial Position	L19.8 P3.9 H0.5
L	19.8 mm
P	3.9 mm
H	0.5 mm
Initial Rotation	0.00 deg
Initial Orientation	Sagittal

Geometry - Saturation

Fat suppr.	None
Wrap-up Magn.	Restore
Special sat.	None

Geometry - Navigator

Geometry - Tim Planning Suite

Set-n-Go Protocol	Off
Table position	H
Table position	0 mm
Inline Composing	Off

System - Miscellaneous

Positioning mode	REF
Table position	H
Table position	0 mm
MSMA	S - C - T
Sagittal	R >> L
Coronal	A >> P
Transversal	F >> H
Coil Combine Mode	Adaptive Combine
Save uncombined	Off
Matrix Optimization	Off
Coil Focus	Flat
AutoAlign	---
Coil Select Mode	Default

System - Adjustments

B0 Shim mode	Standard
B1 Shim mode	TrueForm
Adjust with body coil	On
Confirm freq. adjustment	Off
Assume Dominant Fat	Off
Assume Silicone	Off
Adjustment Tolerance	Auto

System - Adjust Volume

Position	L19.8 P3.9 H0.5 mm
Orientation	Sagittal
Rotation	0.00 deg
A >> P	305 mm
F >> H	380 mm
R >> L	232 mm
Reset	Off

System - pTx Volumes

B1 Shim mode	TrueForm
Excitation	Slice-sel.

System - Tx/Rx

Frequency 1H	123.255690 MHz
Correction factor	1
Gain	High
Img. Scale Cor.	1.000
Reset	Off
? Ref. amplitude 1H	0.000 V

Physio - Signal1

1st Signal/Mode	None
TR	802.07 ms
Concatenations	21
Segments	154

Physio - Cardiac

Tagging	None
Magn. preparation	None
Fat suppr.	None
Dark blood	Off
FoV read	380 mm
FoV phase	80.2 %
Phase resolution	100 %
Cine	Off
Trajectory	Cartesian
Dummy heartbeats	0

Physio - PACE

Resp. control	Off
Concatenations	21

Inline - Common

Subtract	Off
Measurements	1
StdDev	Off
Save original images	On

Inline - Cardiac

Inline Evaluation	Off
Magn. preparation	None
Contrasts	1
TE	1.05 ms
TR	802.07 ms
Save original images	On

Inline - MIP

MIP-Sag	Off
MIP-Cor	Off
MIP-Tra	Off
MIP-Time	Off
Save original images	On

Inline - Composing

Inline Composing	Off
Distortion Corr.	On
Mode	2D
Unfiltered images	Off

Sequence - Part 1

Introduction	Off
Dimension	2D
Reordering	Linear
Asymmetric echo	Weak
Contrasts	1
Optimization	Min. TE
Multi-slice mode	Sequential
Echo spacing	2.5 ms
Sequence type	Trufi
Bandwidth	1132 Hz/Px

Sequence - Part 2

Define	Shots
Shots per slice	1
Segments	154
Trufi delta freq.	0 Hz RF
pulse type	Fast
Gradient mode	Fast
Excitation	Slice-sel.
Flip angle mode	Constant
Cine	Off

Sequence - Assistant

Mode	Off
Allowed delay	0 s

\\Research\Abdominal\SS BEAT Kidney\iBEAT_DKDv10.6\ASL_kidneys_pCASL_cor-oblique_fb
TA: 5:05 PM: REF Voxel size: 4.7×4.7×5.0 mmRel. SNR: 1.00 : tgse

Properties

Prio recon	Off
Load images to viewer	On
Inline movie	Off
Auto store images	On
Load images to stamp segments	On
Load images to graphic segments	On
Auto open inline display	Off
Auto close inline display	Off
Start measurement without further preparation	Off
Wait for user to start	Off
Start measurements	Single measurement

Routine

Slab group	1
Slabs	1
Dist. factor	50 %
Position	L11.8 P48.1 H0.0 mm
Orientation	C > T4.6
Phase enc. dir.	F >> H
AutoAlign	---
Phase oversampling	0 %
Slices per slab	16
FoV read	300 mm
FoV phase	50.0 %
Slice thickness	5.00 mm
TR	5000 ms
TE	19.28 ms
Averages	1
Concatenations	1
Filter	Raw filter, Distortion Corr.(2D)
Coil elements	BO2,3;SP7,8

Contrast - Common

TR	5000 ms
TE	19.28 ms
Flip angle	180 deg
Fat suppr.	Fat sat.
Fat sat. mode	Weak

Contrast - Dynamic

Averages	1
Averaging mode	Long term
Reconstruction	Magnitude
Measurements	30
Delay in TR	0 ms
Multiple series	Off

Resolution - Common

FoV read	300 mm
FoV phase	50.0 %
Slice thickness	5.00 mm
Base resolution	64
Phase resolution	100 %
Phase partial Fourier	Off
Slice partial Fourier	Off
Interpolation	Off

Resolution - Filter Image

Distortion Corr.	On
Mode	2D
Prescan Normalize	Off

Resolution - Filter Rawdata

Raw filter	On
Elliptical filter	Off
Hamming	Off

Geometry - Common

Slab group	1
Slabs	1
Dist. factor	50 %
Position	L11.8 P48.1 H0.0 mm
Orientation	C > T4.6
Phase enc. dir.	F >> H
Slices per slab	16
FoV read	300 mm
FoV phase	50.0 %
Slice thickness	5.00 mm
TR	5000 ms
Multi-slice mode	Interleaved
Series	Ascending
Concatenations	1

Geometry - AutoAlign

Slab group	1
Position	L11.8 P48.1 H0.0 mm
Orientation	C > T4.6
Phase enc. dir.	F >> H
AutoAlign	---
Initial Position	L11.8 P48.1 H0.0
L	11.8 mm
P	48.1 mm
F	0.0 mm
Initial Rotation	90.00 deg
Initial Orientation	C > T
C > T	4.6
> S	0.0

Geometry - Saturation

Sat. region	1
Thickness	10 mm
Position	L0.0 P0.0 H100.0 mm
Orientation	Transversal
Sat. region	2
Thickness	176 mm
Position	R0.8 P1.5 H4.6 mm
Orientation	T > C-1.1 > S0.5
Sat. region	3
Thickness	111 mm
Position	R0.7 P0.0 H162.4 mm
Orientation	T > S0.2
Fat sat. mode	Weak
Special sat.	None

Geometry - Tim Planning Suite

Set-n-Go Protocol	Off
Table position	H
Table position	0 mm

Geometry - Tim Planning Suite

Inline Composing	Off

System - Miscellaneous

Positioning mode	REF
Table position	H
Table position	0 mm
MSMA	S - C - T
Sagittal	R >> L
Coronal	A >> P
Transversal	F >> H
Coil Combine Mode	Adaptive Combine
Matrix Optimization	Off
Coil Focus	Flat
AutoAlign	---
Coil Select Mode	Default

System - Adjustments

B0 Shim mode	Standard
B1 Shim mode	TrueForm
Adjust with body coil	Off
Confirm freq. adjustment	Off
Assume Dominant Fat	Off
Assume Silicone	Off
Adjustment Tolerance	Auto

System - Adjust Volume

Position	L11.8 P48.1 H0.0 mm
Orientation	C > T4.6
Rotation	90.00 deg
F >> H	150 mm
R >> L	300 mm
A >> P	80 mm
Reset	Off

System - pTx Volumes

B1 Shim mode	TrueForm

System - Tx/Rx

Frequency 1H	123.255690 MHz
Correction factor	1
Gain	Low
Img. Scale Cor.	1.000
Reset	Off
? Ref. amplitude 1H	0.000 V

Physio - Signal1

1st Signal/Mode	None
TR	5000 ms
Concatenations	1
Segments	1

Sequence - Part 1

Introduction	Off
Dimension	3D
Reordering	Centric
Multi-slice mode	Interleaved
Echo spacing	0.37 ms
Bandwidth	3552 Hz/Px

Sequence - Part 2

EPI factor	32
Segments	1
RF pulse type	Low SAR
Gradient mode	Fast
Turbo factor	16

Sequence - Special

Perfusion mode	pCASL
Start TI	3000 ms
Number of TI	1 #
PCASL duration	1500 ms
PCASL flip angle	28.0 deg
Pre sat scale	1.5
Spoiler duration	5000 us
No of sats	3
Mean Gz	1.00 mT/m
T1 Blood	1200.0 ms
RBF filter	Off
Motion correction	On
Save original	Off

\\Research\Abdominal\SS BEAT Kidney\iBEAT_DKDv10.6\DCE_kidneys_cor-oblique_fb
TA: 7:07 PM: REF Voxel size: 1.0×1.0×7.5 mmPAT: 2 Rel. SNR: 1.00 : tfl

Properties

Prio recon	Off
Load images to viewer	On
Inline movie	Off
Auto store images	On
Load images to stamp segments	On
Load images to graphic segments	On
Auto open inline display	On
Auto close inline display	On
Start measurement without further preparation	Off
Wait for user to start	On
Start measurements	Single measurement

Routine

Slice group	1
Slices	8
Dist. factor	20 %
Position	L11.8 P42.4 H0.0 mm
Orientation	C > T4.6
Phase enc. dir.	R >> L
Slice group	2
Slices	1
Dist. factor	20 %
Position	L10.9 P11.4 H80.7 mm
Orientation	Transversal
Phase enc. dir.	A >> P
AutoAlign	---
Phase oversampling	0 %
FoV read	400 mm
FoV phase	100.0 %
Slice thickness	7.5 mm
TR	179.0 ms
TE	0.97 ms
Averages	1
Concatenations	9
Filter	Distortion Corr.(2D)
Coil elements	BO1-3;SP6-8

Contrast - Common

TR	179.0 ms
TE	0.97 ms
TD	0 ms
Magn. preparation	Non-sel. SR perf
TI 1	85 ms
Flip angle	10 deg
Fat suppr.	None
Water suppr.	None

Contrast - Dynamic

Averages	1
Averaging mode	Short term
Reconstruction	Magnitude
Measurements	265
Pause after meas.	0.0 s
Multiple series	Off

Resolution - Common

FoV read	400 mm
FoV phase	100.0 %
Slice thickness	7.5 mm

Resolution - Common

Base resolution	192
Phase resolution	75 %
Phase partial Fourier	7/8
Interpolation	On

Resolution - iPAT

PAT mode	GRAPPA
Accel. factor PE	2
Ref. lines PE	24
Reference scan mode	Integrated

Resolution - Filter Image

Image Filter	Off
Distortion Corr.	On
Mode	2D
Unfiltered images	Off
Prescan Normalize	Off
Normalize	Off
B1 filter	Off

Resolution - Filter Rawdata

Raw filter	Off
Elliptical filter	Off

Geometry - Common

Slice group	1
Slices	8
Dist. factor	20 %
Position	L11.8 P42.4 H0.0 mm
Orientation	C > T4.6
Phase enc. dir.	R >> L
Slice group	2
Slices	1
Dist. factor	20 %
Position	L10.9 P11.4 H80.7 mm
Orientation	Transversal
Phase enc. dir.	A >> P
FoV read	400 mm
FoV phase	100.0 %
Slice thickness	7.5 mm
TR	179.0 ms
Multi-slice mode	Sequential
Series	Interleaved
Concatenations	9

Geometry - AutoAlign

Slice group	1
Position	L11.8 P42.4 H0.0 mm
Orientation	C > T4.6
Phase enc. dir.	R >> L
Slice group	2
Position	L10.9 P11.4 H80.7 mm
Orientation	Transversal
Phase enc. dir.	A >> P
AutoAlign	---
Initial Position	L11.8 P42.4 H0.0
L	11.8 mm
P	42.4 mm
F	0.0 mm
Initial Rotation	0.00 deg

Geometry - AutoAlign

Initial Orientation	C > T
C > T	4.6
> S	0.0

Geometry - Navigator

Geometry - Tim Planning Suite

Set-n-Go Protocol	Off
Table position	H
Table position	0 mm
Inline Composing	Off

System - Miscellaneous

Positioning mode	REF
Table position	H
Table position	0 mm
MSMA	S - C - T
Sagittal	R >> L
Coronal	A >> P
Transversal	F >> H
Coil Combine Mode	Adaptive Combine
Save uncombined	Off
Matrix Optimization	Off
Coil Focus	Flat
AutoAlign	---
Coil Select Mode	Default

System - Adjustments

B0 Shim mode	Tune up
B1 Shim mode	TrueForm
Adjust with body coil	Off
Confirm freq. adjustment	Off
Assume Dominant Fat	Off
Assume Silicone	Off
Adjustment Tolerance	Auto

System - Adjust Volume

Position	Isocenter
Orientation	Transversal
Rotation	0.00 deg
A >> P	263 mm
R >> L	350 mm
F >> H	350 mm
Reset	Off

System - pTx Volumes

B1 Shim mode	TrueForm
Excitation	Slice-sel.

System - Tx/Rx

Frequency 1H	123.255690 MHz
Correction factor	1
Gain	High
Img. Scale Cor.	1.000
Reset	Off
? Ref. amplitude 1H	0.000 V

Physio - Signal1

1st Signal/Mode	None
TR	179.0 ms
Concatenations	9

Physio - Cardiac

Magn. preparation	Non-sel. SR perf
TI 1	85 ms
Fat suppr.	None
Dark blood	Off
FoV read	400 mm
FoV phase	100.0 %
Phase resolution	75 %

Physio - PACE

Resp. control	Off
Concatenations	9

Inline - Common

Subtract	Off
Measurements	265
StdDev	Off
Save original images	On

Inline - MIP

MIP-Sag	Off
MIP-Cor	Off
MIP-Tra	Off
MIP-Time	Off
Save original images	On

Inline - Composing

Inline Composing	Off
Distortion Corr.	On
Mode	2D
Unfiltered images	Off

Inline - MapIt

Save original images	On
MapIt	None
Flip angle	10 deg
Measurements	265
TR	179.0 ms
TE	0.97 ms

Sequence - Part 1

Introduction	Off
Dimension	2D
Asymmetric echo	Allowed
Flow comp.	No
Multi-slice mode	Sequential
Echo spacing	2.2 ms
Bandwidth	930 Hz/Px

Sequence - Part 2

RF pulse type	Fast
Gradient mode	Fast
Excitation	Slice-sel.
RF spoiling	On
Incr. Gradient spoiling	On
Turbo factor	126

Sequence - Assistant

Mode	Off

\\Research\Abdominal\SS BEAT Kidney\iBEAT_DKDv10.6\T1w_abdomen_post_contrast_dixon_cor_bh
TA: 0:20 PM: FIX Voxel size: 1.3×1.3×1.5 mmPAT: 6 Rel. SNR: 1.00 : fl

Properties

Prio recon	Off
Load images to viewer	On
Inline movie	Off
Auto store images	On
Load images to stamp segments	On
Load images to graphic segments	On
Auto open inline display	On
Auto close inline display	Off
Start measurement without further preparation	Off
Wait for user to start	On
Start measurements	Single measurement

Routine

Slab group	1
Slabs	1
Dist. factor	20 %
Position	L15.3 P10.3 H70.1 mm
Orientation	Coronal
Phase enc. dir.	R >> L
AutoAlign	---
Phase oversampling	20 %
Slice oversampling	22.2 %
Slices per slab	144
FoV read	400 mm
FoV phase	100.0 %
Slice thickness	1.5 mm
TR	4.01 ms
TE 1	1.34 ms
TE 2	2.57 ms
Averages	1
Concatenations	1
Filter	Distortion Corr.(2D), Prescan Normalize
Coil elements	BO1-3;SP6-8

Contrast - Common

TR	4.01 ms
TE 1	1.34 ms
TE 2	2.57 ms
Flip angle	9.0 deg
Fat suppr.	None
Water suppr.	None
Dixon	On

Contrast - Dynamic

Averages	1
Averaging mode	Long term
Reconstruction	Magnitude
Measurements	1
Multiple series	Off

Resolution - Common

FoV read	400 mm
FoV phase	100.0 %
Slice thickness	1.5 mm
Base resolution	320
Phase resolution	80 %
Slice resolution	60 %

Resolution - Common

Phase partial Fourier	7/8
Slice partial Fourier	7/8
Trajectory	Cartesian
View sharing	Off
Interpolation	Off

Resolution - iPAT

PAT mode	CAIPIRINHA
Accel. factor PE	3
Ref. lines PE	24
Accel. factor 3D	2
Ref. lines 3D	28
Reordering Shift 3D	0
Reference scan mode	GRE/separate
CAIPIRINHA mode	Body Tra
Total PAT factor	6

Resolution - Filter Image

Image Filter	Off
Distortion Corr.	On
Mode	2D
Unfiltered images	Off
Prescan Normalize	On
Unfiltered images	Off
Normalize	Off
B1 filter	Off

Resolution - Filter Rawdata

Raw filter	Off
Elliptical filter	Off
POCS	Off

Geometry - Common

Slab group	1
Slabs	1
Dist. factor	20 %
Position	L15.3 P10.3 H70.1 mm
Orientation	Coronal
Phase enc. dir.	R >> L
Slice oversampling	22.2 %
Slices per slab	144
FoV read	400 mm
FoV phase	100.0 %
Slice thickness	1.5 mm
TR	4.01 ms
Multi-slice mode	Sequential
Series	Ascending
Concatenations	1

Geometry - AutoAlign

Slab group	1
Position	L15.3 P10.3 H70.1 mm
Orientation	Coronal
Phase enc. dir.	R >> L
AutoAlign	---
Initial Position	L15.3 P10.3 H6.1
L	15.3 mm
P	10.3 mm
H	6.1 mm

Geometry - AutoAlign

Initial Rotation	0.00 deg
Initial Orientation	Coronal

Geometry - Saturation

Fat suppr.	None
Water suppr.	None
Dixon	On
Special sat.	None

Geometry - Tim Planning Suite

Set-n-Go Protocol	Off
Table position	H
Table position	64 mm
Inline Composing	Off

System - Miscellaneous

Positioning mode	FIX
Table position	H
Table position	64 mm
MSMA	S - C - T
Sagittal	R >> L
Coronal	A >> P
Transversal	H >> F
Coil Combine Mode	Adaptive Combine
Save uncombined	Off
Matrix Optimization	Off
Coil Focus	Flat
AutoAlign	---
Coil Select Mode	Off - All

System - Adjustments

B0 Shim mode	Standard
B1 Shim mode	TrueForm
Adjust with body coil	Off
Confirm freq. adjustment	Off
Assume Dominant Fat	Off
Assume Silicone	Off
Adjustment Tolerance	Auto

System - Adjust Volume

Position	L15.3 P10.3 H70.1 mm
Orientation	Coronal
Rotation	0.00 deg
R >> L	400 mm
F >> H	400 mm
A >> P	216 mm
Reset	Off

System - pTx Volumes

B1 Shim mode	TrueForm
Excitation	Slab-sel.

System - Tx/Rx

Frequency 1H	123.255690 MHz
Correction factor	1
Gain	Low
Img. Scale Cor.	1.000
Reset	Off
? Ref. amplitude 1H	0.000 V

Physio - PACE

Resp. control	Breath-hold
Concatenations	1

Inline - Common

View sharing	Off
Flip angle	9.0 deg
Measurements	1
Burn time-to-center	Off
Temporal interpolation	1
3D centric reordering	Off
Time to center	10.1 s

Inline - Inline

Subtract	Off
Measurements	1
StdDev	Off
Liver registration	Off
Save original images	On

Inline - MIP

MIP-Sag	Off
MIP-Cor	Off
MIP-Tra	Off
MIP-Time	Off
Save original images	On

Inline - Soft Tissue

Wash - In	Off
Wash - Out	Off
TTP	Off
PEI	Off
MIP - time	Off
Measurements	1

Inline - Composing

Inline Composing	Off
Distortion Corr.	On
Mode	2D
Unfiltered images	Off

Inline - MapIt

Save original images	On
MapIt	None
Flip angle	9.0 deg
Measurements	1
Contrasts	2
TR	4.01 ms
TE 1	1.34 ms
TE 2	2.57 ms

Sequence - Part 1

Introduction	Off
Dimension	3D
Elliptical scanning	Off
Asymmetric echo	Weak
Contrasts	2
Readout mode	Bipolar
Optimization	Opp/In
Multi-slice mode	Sequential
Bandwidth 1	820 Hz/Px
Bandwidth 2	1040 Hz/Px

Sequence - Part 2

RF pulse type	Fast
Gradient mode	Fast
Excitation	Slab-sel.
RF spoiling	On

Sequence - Part 2

Incr. Gradient spoiling	On

Sequence - Assistant

Mode	Off
Allowed delay	60 s
