# Supplementary material for: Prognostic imaging biomarkers for diabetic kidney disease (iBEAt): study protocol
Source: BMC Nephrol. 2020 Jun 29;21:242. doi: 10.1186/s12882-020-01901-x (PMC7323369; doi:10.1186/s12882-020-01901-x)

Prognostic Imaging Biomarkers for Diabetic Kidney Disease (iBEAt)

Ultrasound Standard Operating Procedures

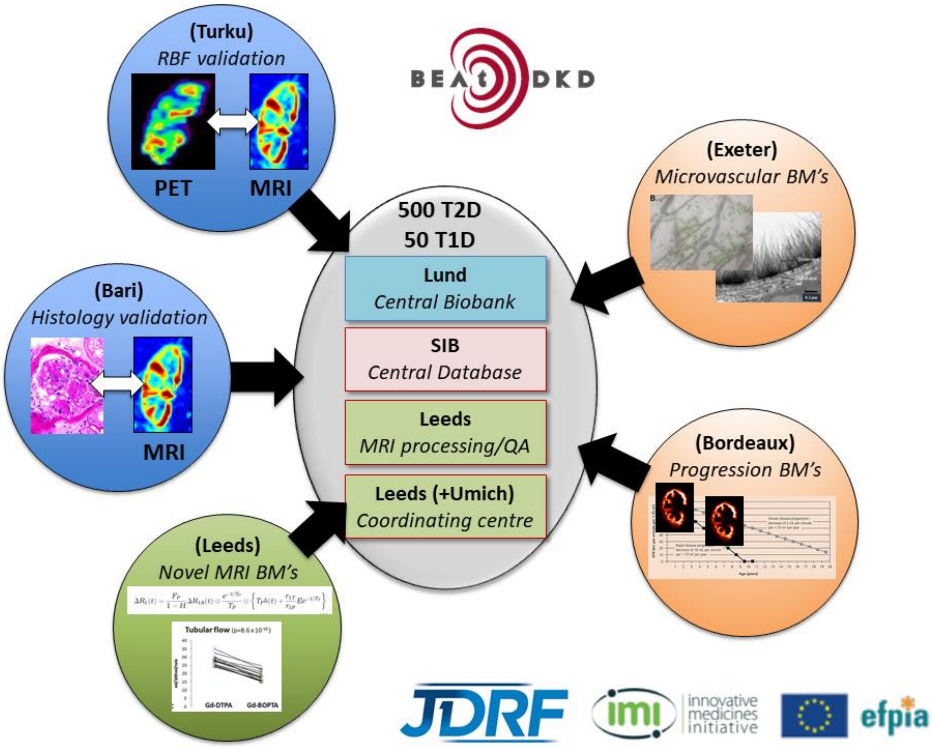

Version 1.0
09.10.2019

Authors: Nicolas Grenier, Sapna Puppala

Description: Standard Operating Procedure for Ultrasound scanning in iBEAt

Preparation:
Fasting is not required. Hydration before the examination may be necessary to fill the bladder.

Technique:
Each kidney has to be scanned with a low frequency probe (3-5MHz) in longitudinal and transverse planes.

<u>Longitudinal plane:</u>
This plane is important:

- to evaluate the cortical echogenicity compared liver or spleen (usually lower)
- to evaluate the corticomedullary differentiation: pyramids (medulla) normally show lower echogenicity than cortex
- to measure the long axis of each kidney: difficult, requires placing the probe along the exact long axis of each kidney taking into account their double obliquity (in frontal and sagittal planes)
- to detect pyelectasis
  At least one image of each kidney, including a measurement of the maximum renal length will be stored.

<u>Transverse plane:</u>
This plane shows renal contours (smooth and regular) and the pelvis.
At least one image of each kidney, including renal hilum with vessels and renal pelvis will be stored.

<u>Renal resistive index assessment</u>
This Doppler sampling is performed with the same probe, using color and spectral modes.
Resistive index (RI) is the most commonly used variable in Doppler imaging to evaluate blood flow in vessels. As it is a calculation of the relationship between systole and diastole, it is an indicator of the resistance to flow within the kidney.
RI is very reproducible. It is calculated as a ratio of two velocities obtained from the same transducer angle (so no need for angle correction, unlike velocity assessment).
In the kidney, RI is determined by assessing systolic and diastolic blood velocity in the interlobar (or segmental) arteries and applying the following formula:

Peak systolic velocity - end diastolic velocity / peak systolic velocity

Three measurements must be done for each kidney: upper pole, mid-pole, lower pole. Then a mean value is calculated from the three samplings.
Normal renal RI is approximately 0.6 (ranging between 0.56 and 0.66).

Example of RI sampling

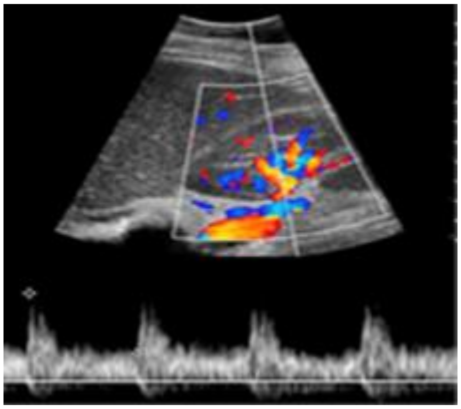

Anatomy of intrarenal vessels

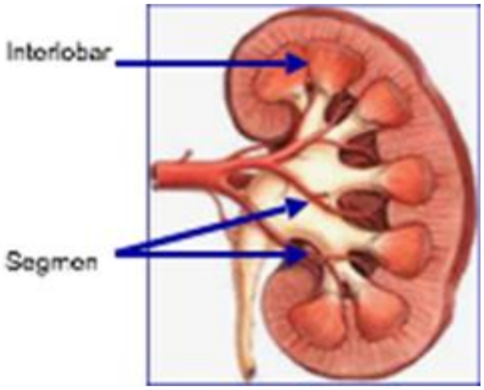

Normal intrarenal flow patterns

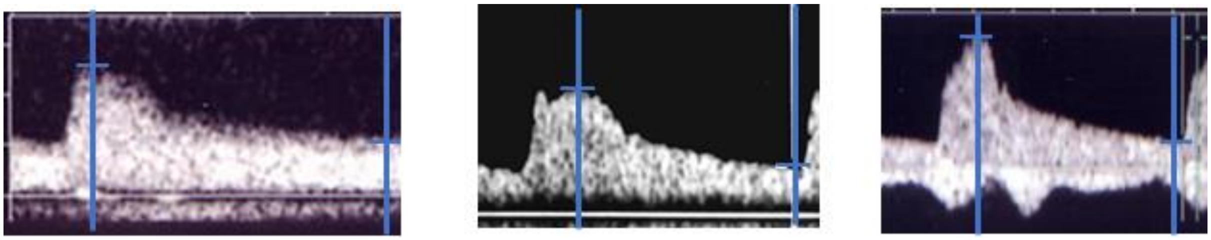

Any abnormality detected within abdominal or pelvic organ or on abdominal aorta must require a specialized advice

Elastography (in Bordeaux)
Shearwave velocity will be measured on each kidney using SSI (Supersonic Shearwave Imaging) technique (Aixplorer, Supersonic Imagine, Aix en Provence, France) Five measurements will be performed on each kidney.

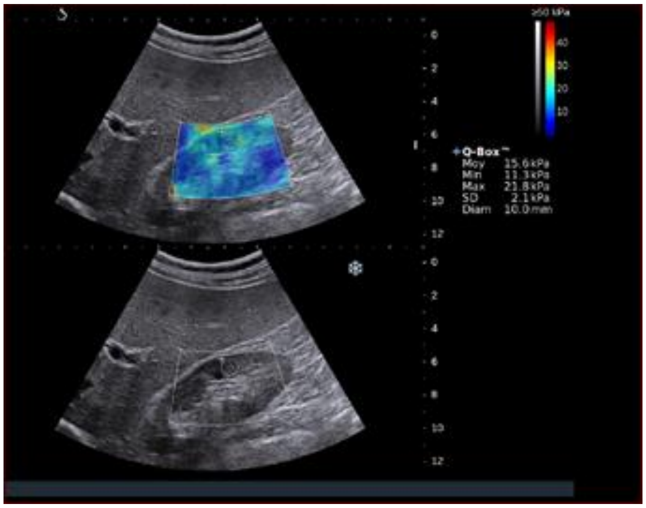

Supplement: Supplementary file 1 — Additional file 1: 1.1 MRI biomarkers. File type: PDF file. Title: List of primary MRI biomarkers. Description: A table listing the biomarkers that will be derived from the MRI data to address the primary objectives. 1.2 MRI acquisition protocol. PDF file. MRI acquisition protocol (reference scanner). MRI sequence parameters for the iBEAt protocol on the reference scanner (Siemens 3 T). 1.3 Renal ultrasound SOP. PDF file. Ultrasound Standard Operating Procedures. Standard operating procedures for Ultrasound scanning in iBEAt. [file 12882_2020_1901_MOESM1_ESM.zip › Additional file 1.3. Renal ultrasound SOP_ESM.pdf]
