# Supplementary material for: Prognostic imaging biomarkers for diabetic kidney disease (iBEAt): study protocol
Source: BMC Nephrol. 2020 Jun 29;21:242. doi: 10.1186/s12882-020-01901-x (PMC7323369; doi:10.1186/s12882-020-01901-x)
Supplement: Supplementary file 2 — Additional file 2: 2.1 Biofluid collection SOPs. PDF file. Biofluid collection protocol. The protocol for the collection of blood and urine samples within iBEAt. 2.2 SOPs Biofluid processing. PDF file. Biofluid processing protocol. The protocol for processing blood and urine samples within iBEAt. 2.3 Biofluid schematics. PDF file. iBEAt kit contents and biofluid processing schematics. Schematics of iBEAt collection kits, and processing and storage protocols for collected blood and urine samples within iBEAt. [file 12882_2020_1901_MOESM2_ESM.zip › Additional file 2.3 Biofluid SchematicsR1.pdf]

Prognostic Imaging Biomarkers for Diabetic Kidney Disease (iBEAT)

iBEAT kit contents and biofluid processing schematics

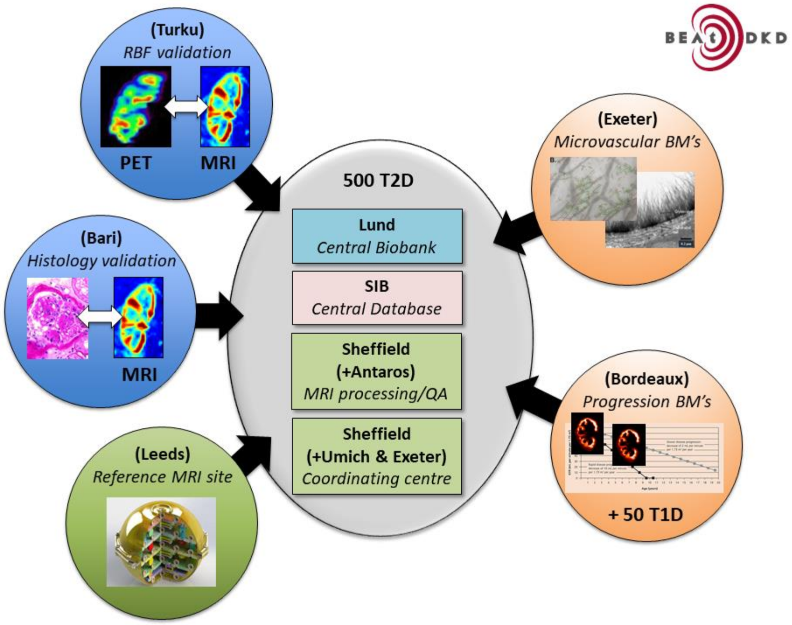

Version 2.0
03.04.2019

One iBEAt biofluids kit contains 2 bags

One iBEAT kit/participant; codes under tubes reflect the agreed texts on labels

”Collection tubes (to the clinic)”

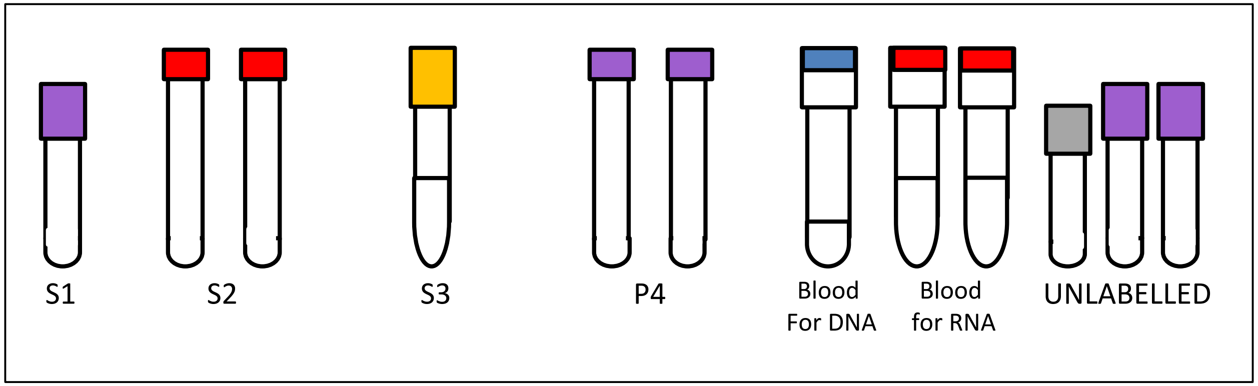

”Processing tubes”

NOTE: Tubes sorted in 2 smaller bags

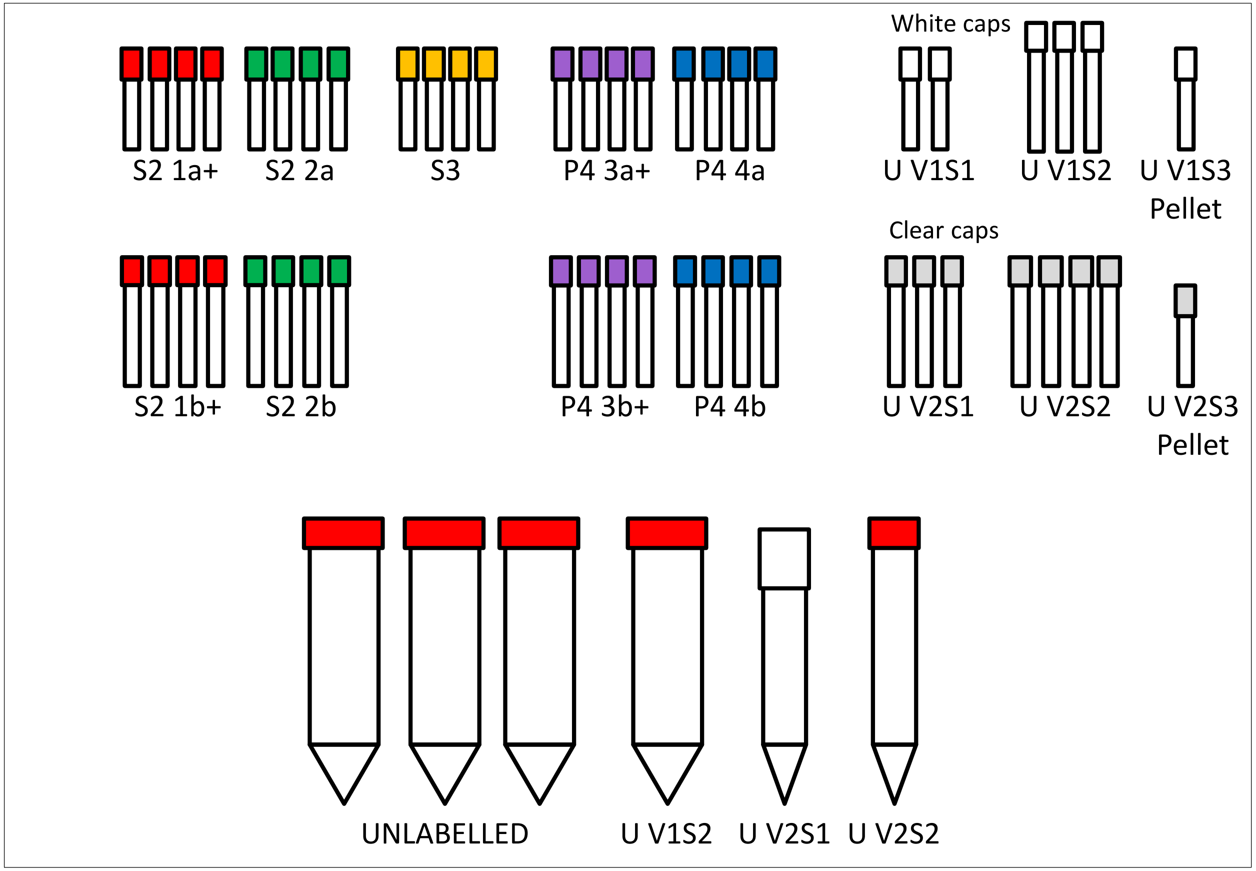

Only the tubes highlighted below (solid lines) will be sent back to the Central Biorepository Lab in Malmö

”Collection tubes” (back from clinic)

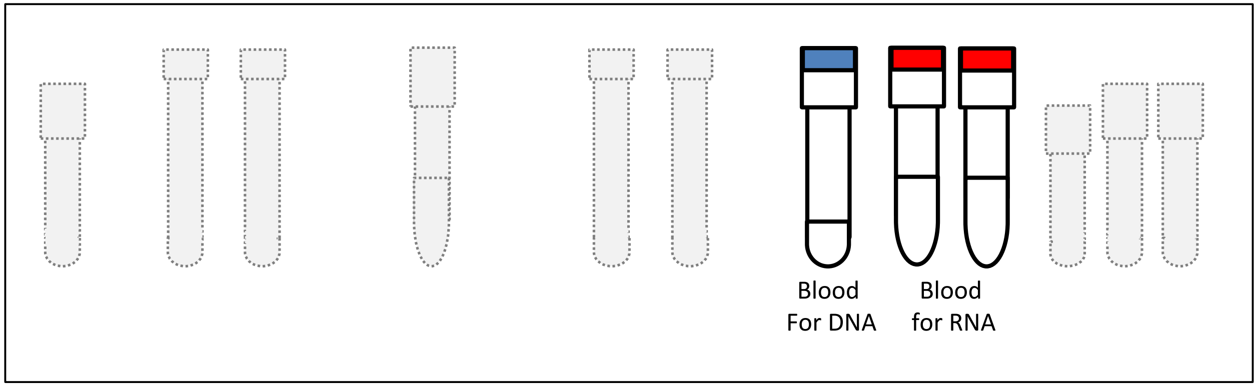

”Processing tubes”

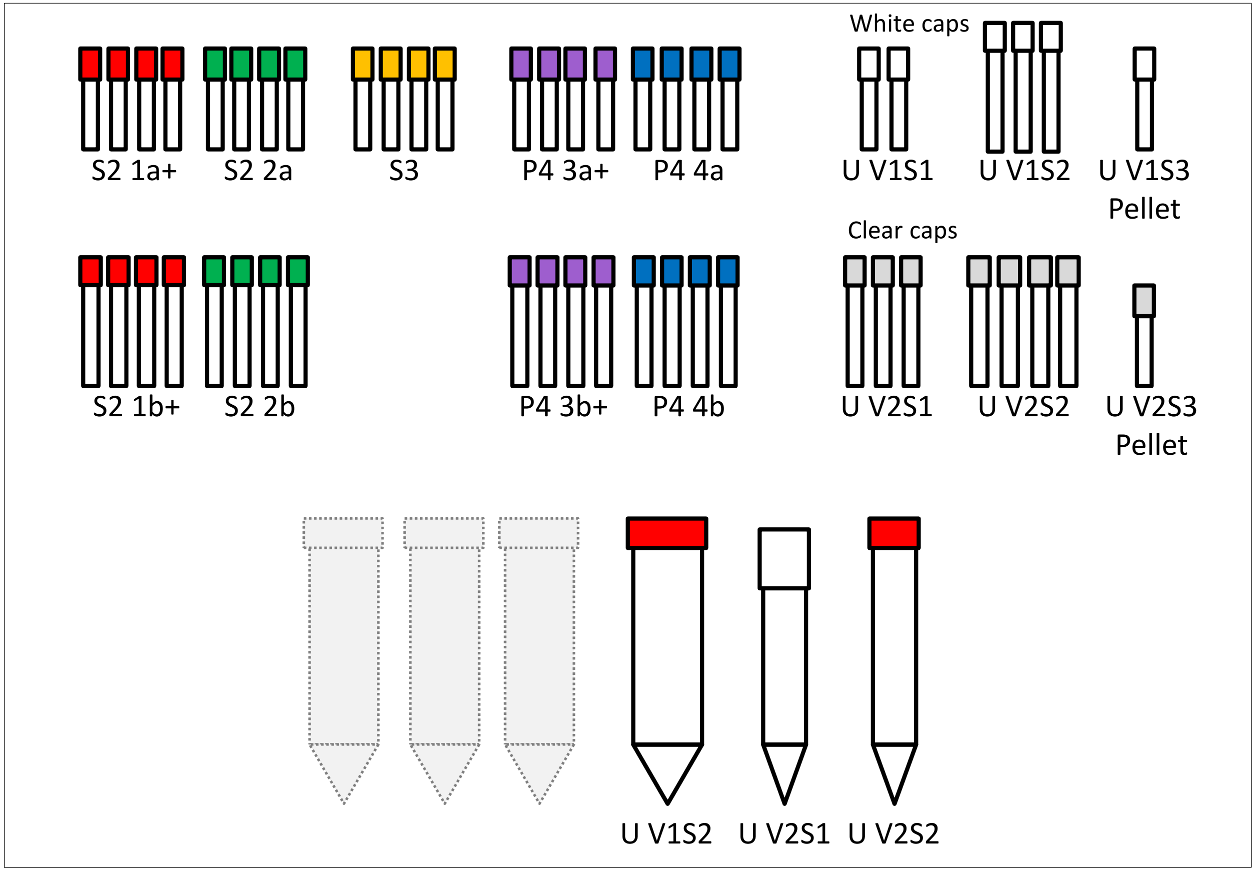

Tube legend schematics

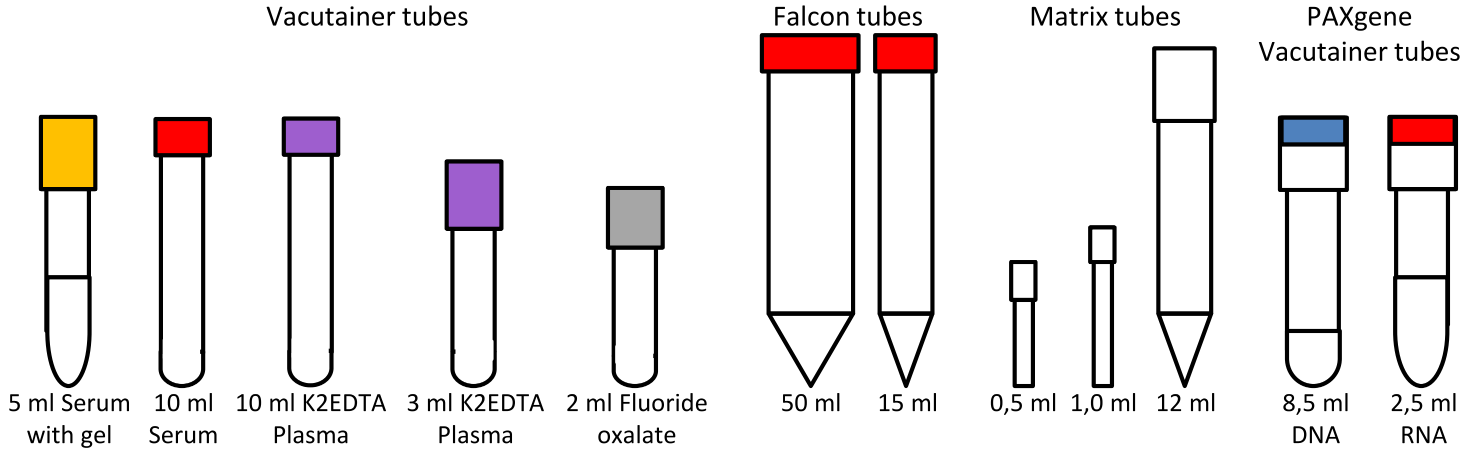

Each center receives one additional bag of spare tubes including:

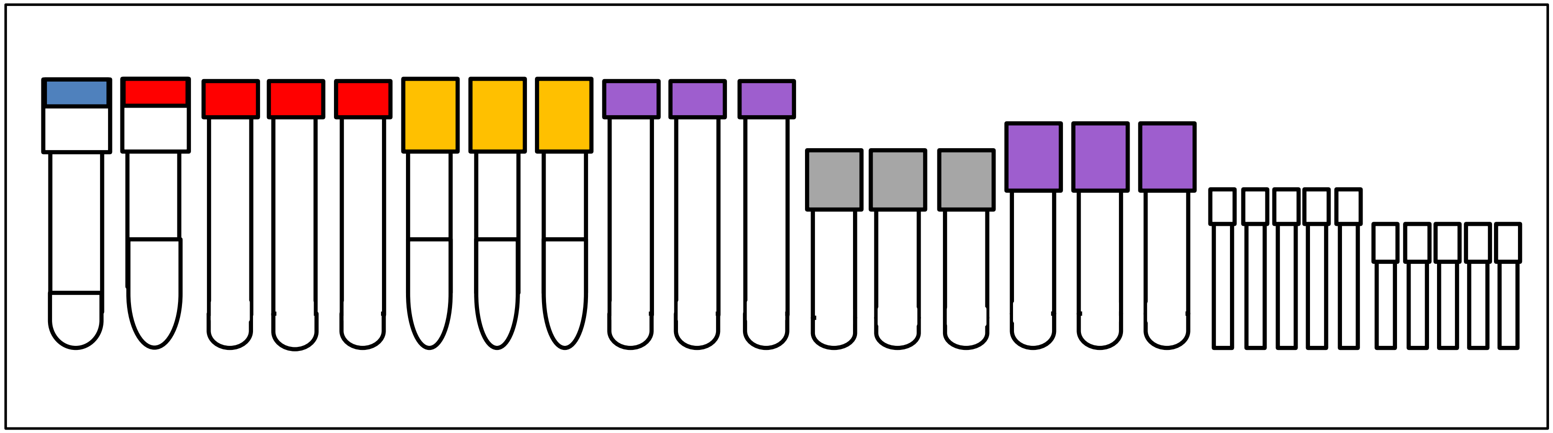

NOTE: Please keep track of expiry dates for the spare tubes and alert the Central Biorepository Lab know if new tubes are needed

Urine Void 1 processing

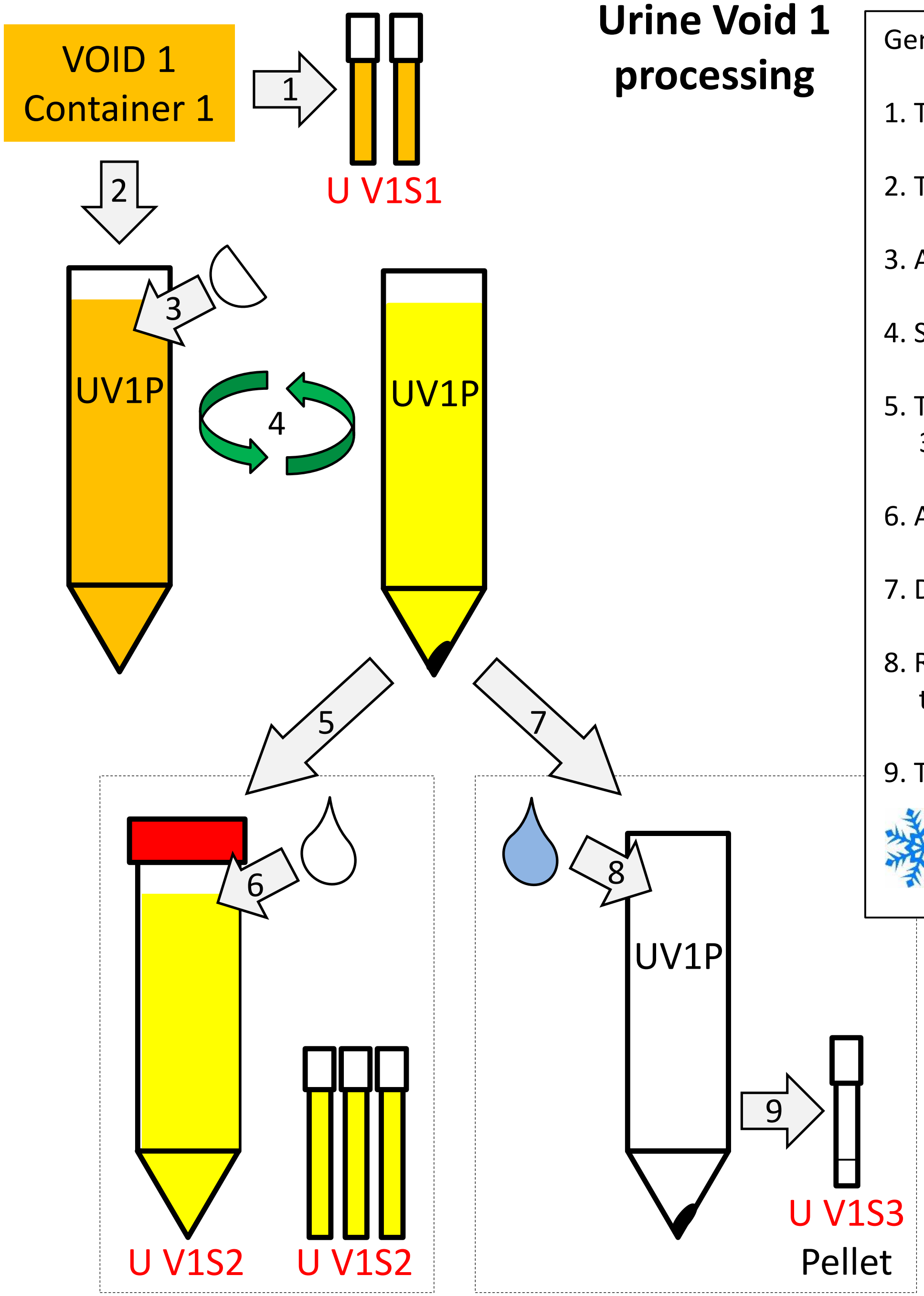

Gently invert the Void 1 container 1 8-10 times before processing

1. Transfer 2x500 µl unspun urine to 2x **U V1S1** (0,5 ml cryovials)

2. Transfer 50 ml unspun urine to a processing tube (mark as UV1P)

3. Add ½ protease inhibitor tablet

4. Spin for 10 min @ 3000xG at room temperature, w/o brake

5. Transfer 45 ml supernatant to **U V1S2** (50 ml tube) &
   3x900 µl to 3x **U V1S2** (1,0 ml cryovials)

6. Add 2,5 ml citrate buffer to **U V1S2** (50 ml tube)

7. Drain residual supernatant from processing tube

8. Resuspend the pellet by adding 100-300 µl RNAlater (depending on the size of the pellet) to the processing tube (UV1P)

9. Transfer the resupended pellet to **U V1S3** (0,5 ml cryovial)

Freeze all RED labeled tubes and cryovials at -80 °C

Urine Void 2 processing

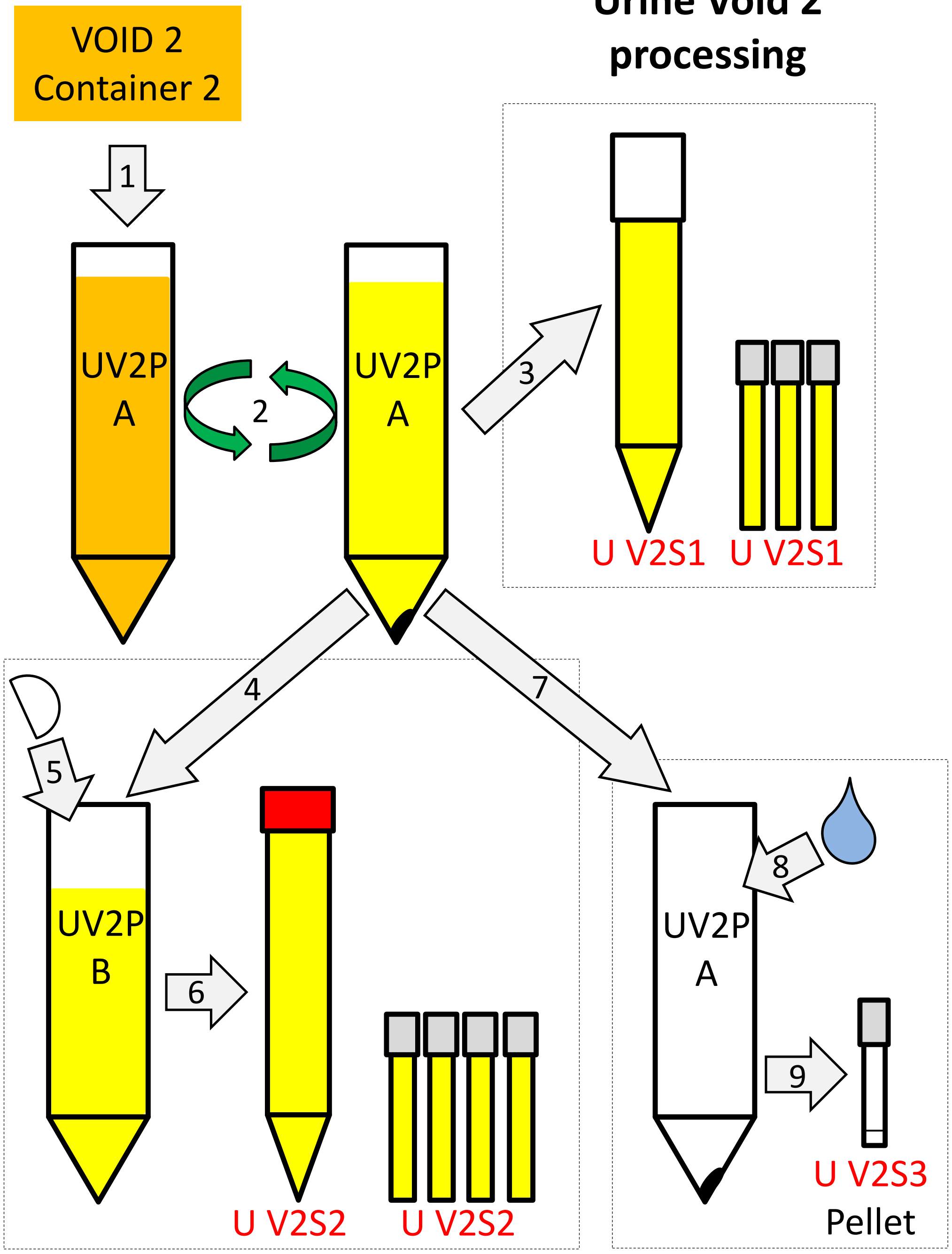

Gently invert the Void 2 container 2 8-10 times before processing

1. Transfer 50 ml whole urine to a processing tube (mark as U V2P A)

2. Spin for 10 min @ 3000xG at room temperature, w/o brake Note the time

3. Transfer 9 ml supernatant to **U V2S1** (12 ml tube) & 3x900 µl to 3x **U V2S1** (1,0 ml cryovials)

4. Transfer 38 ml supernatant to the second processing tube (mark as U V2P B)

5. Add ½ protease inhibitor tablet

6. Transfer 13 ml supernatant to **U V2S2** (15 ml tube) & 4x900 µl to 4x **U V2S2** (1,0 ml cryovials)

7. Drain residual supernatant from processing tube (UV2P A)

8. Resuspend the pellet by adding 100-300 µl RNAlater (depending on the size of the pellet) to the processing tube (UV2P A)

9. Transfer the resupended pellet to **U V2S3** (0,5 ml cryovial)

Freeze all RED labeled tubes and cryovials at -80 °C

Blood collection

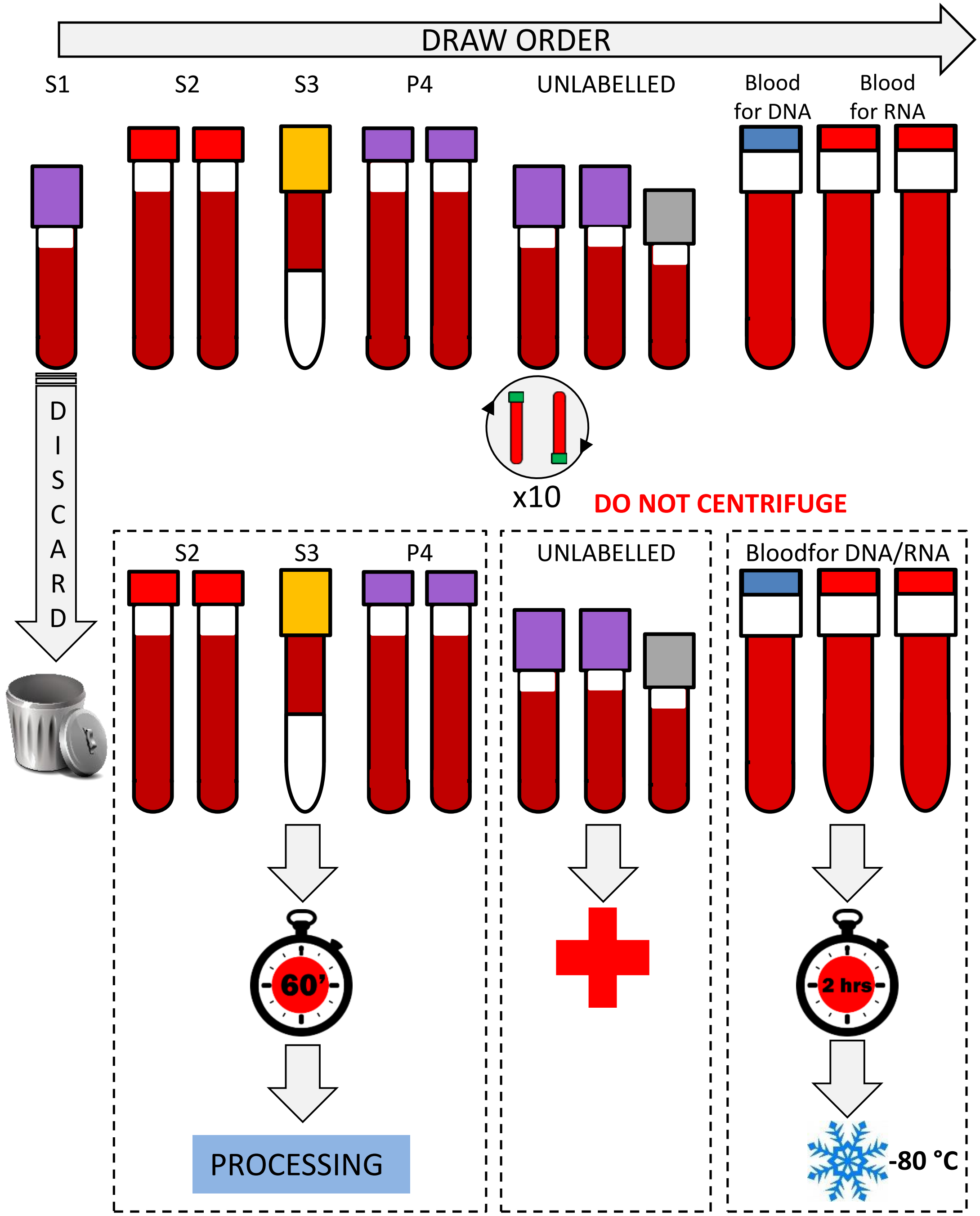

All tubes

- Invert all tubes gently 8-10 times after blood is drawn.
- Note the time the blood draw begins

S2, S3 and P4

- Store at room temperature for 30-60 minutes
- Send to processing lab or refrigerate at 4 °C for no longer than 4 hours prior to processing

UNLABELLED (Samples 5-6)

- Label with hospital patient ID and send to local lab for measurements

Blood for DNA/RNA (Samples 7-8)

- Store at room temperature for 2 hours
- Freeze at -80 °C

What happens with each blood collection tube?

NOTE: If the S1 tube (WASTE) is sent back from the clinic, dispose of it according to site practice

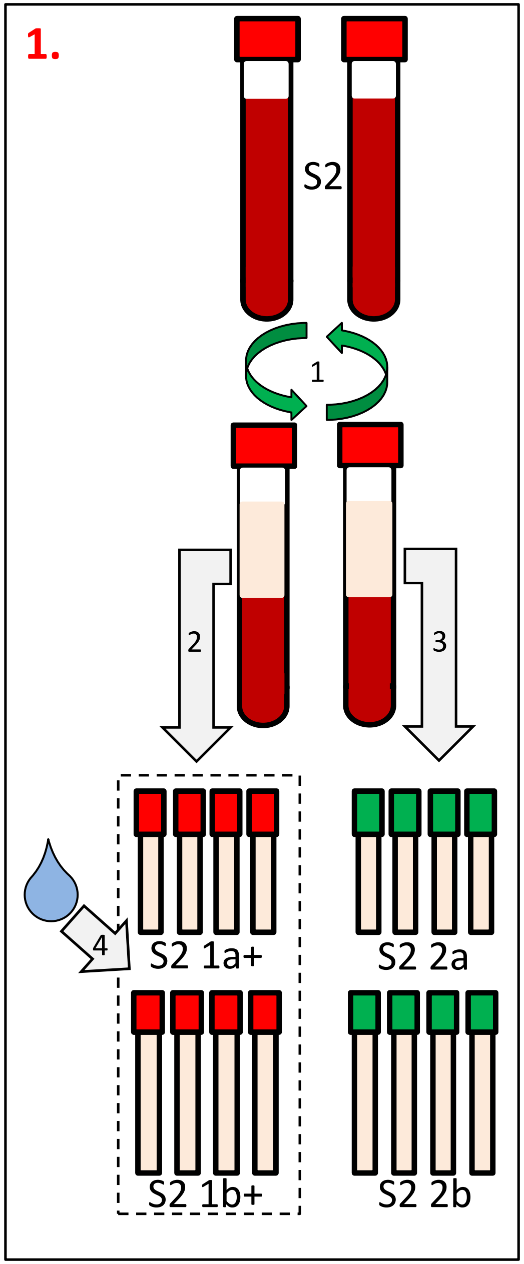

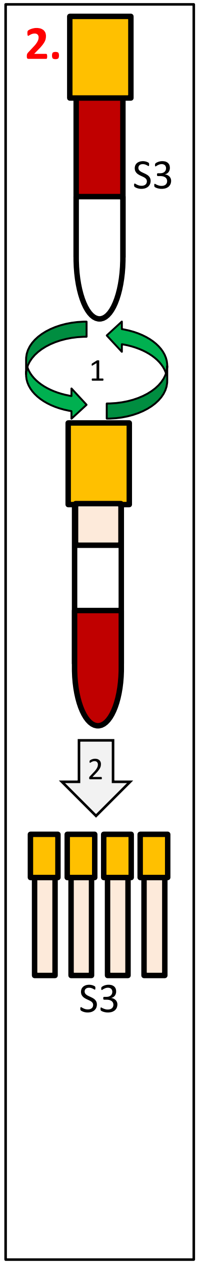

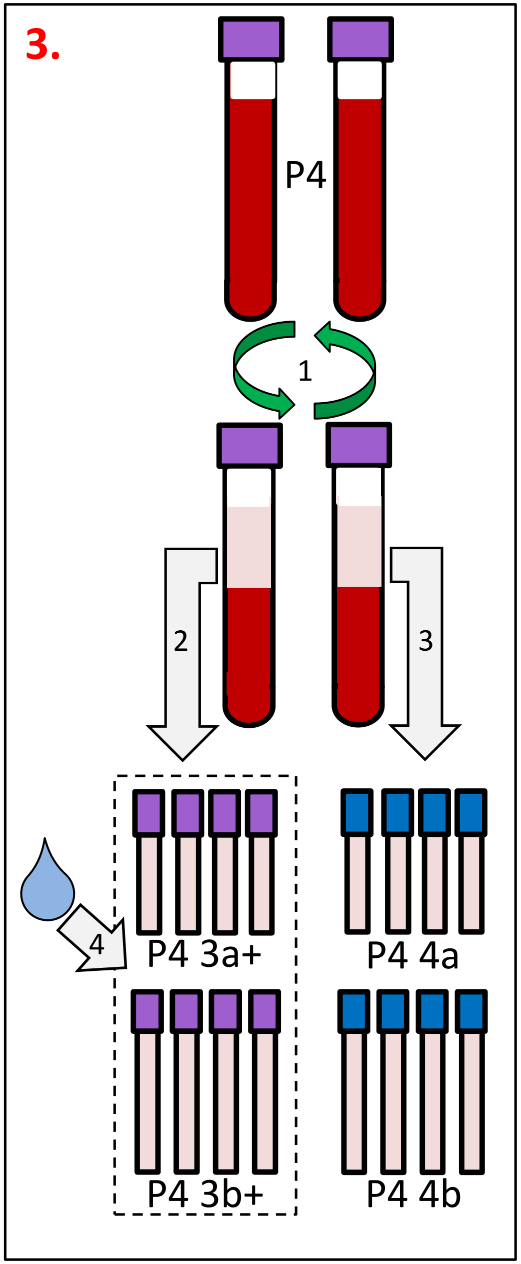

1. S2 (10 ml serum tubes x2)

1.Centrifuge at 2000xG for 12 min. at room temperature

2. Transfer 4x 500 µl serum to 4x **S2 1a+** (0,5 ml cryovials)
   Transfer 4x 900 µl serum to 4x **S2 1b+** (1,0 ml cryovials)

3. Transfer 4x 500 µl serum to 4x **S2 2a** (0,5 ml cryovials)
   Transfer 4x 900 µl serum to 4x **S2 2b** (1,0 ml cryovials)

4. Add 5 µl BHT to the 4x **S2 1a+** and 9 µl BHT 4x **S2 1b+** vials (red caps)

2. S3 (5 ml serum tube with gel)

1. Centrifuge at 2000xG for 12 min. at room temperature

2. Transfer 4x500 µl serum to 4x **S3** vials (0,5 ml cryovials)

3. P4 (10 ml K2EDTA plasma tubes x2)

1. Centrifuge at 2000xG for 12 min. at room temperature

2. Transfer 4x 500 µl plasma to 4x **P4 3a+** (0,5 ml cryovials)
   Transfer 4x 900 µl plasma to 4x **P4 3b+** (1,0 ml cryovials)

3. Transfer 4x 500 µl plasma to 4x **P4 4a** (0,5 ml cryovials)
   Transfer 4x 900 µl plasma to 4x **P4 4b** (1,0 ml cryovials)

4. Add 5 µl BHT to the 4x **P4 3a+** and 9 µl BHT to the 4x **P4 3b+** vials (purple caps)

What happens with each blood collection tube? (continued)

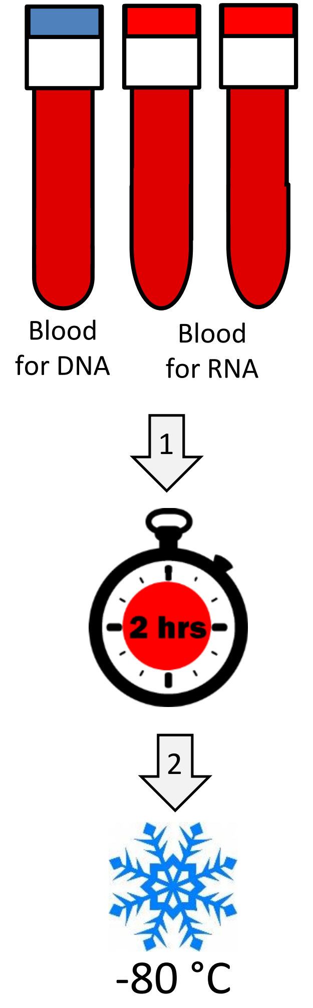

Blood for RNA and DNA (PAXGene tubes x3)

Do <u>not</u> centrifuge!

1. Place upright at room temperature for 120 minutes
   Record exact time kept at room temperature

2. Freeze at -80 °C
