# Supplementary material for: Prognostic imaging biomarkers for diabetic kidney disease (iBEAt): study protocol
Source: BMC Nephrol. 2020 Jun 29;21:242. doi: 10.1186/s12882-020-01901-x (PMC7323369; doi:10.1186/s12882-020-01901-x)
Supplement: Supplementary file 2 — Additional file 2: 2.1 Biofluid collection SOPs. PDF file. Biofluid collection protocol. The protocol for the collection of blood and urine samples within iBEAt. 2.2 SOPs Biofluid processing. PDF file. Biofluid processing protocol. The protocol for processing blood and urine samples within iBEAt. 2.3 Biofluid schematics. PDF file. iBEAt kit contents and biofluid processing schematics. Schematics of iBEAt collection kits, and processing and storage protocols for collected blood and urine samples within iBEAt. [file 12882_2020_1901_MOESM2_ESM.zip › Additional file 2.1 biofluid collection SOPSR1.pdf]

Prognostic Imaging Biomarkers for Diabetic Kidney Disease (iBEAt)

Biofluids collection protocol

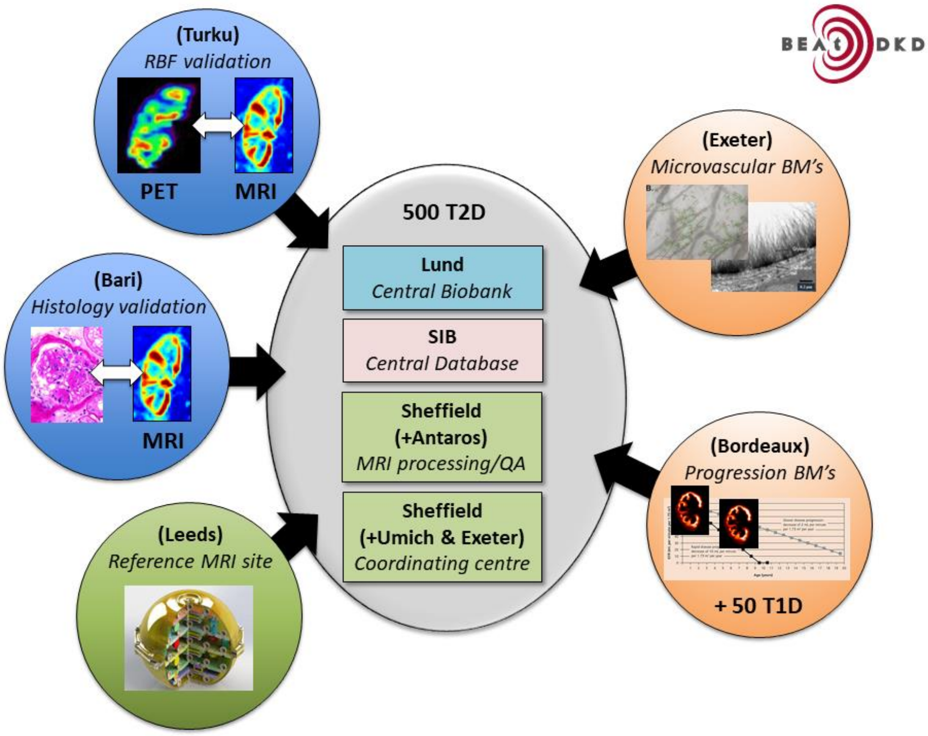

Version 2.0
07.08.2019

TABLE OF CONTENTS

1. Introduction 3
2. Blood Collection 3
   - *2.1 Required Supplies:* 3
   - *2.2 Draw order* 3
   - *2.3 Waste Sample 1:* 4
   - *2.4 Serum Samples 2 and 3:* 4
   - *2.5 K2EDTA Plasma Samples 4 and 5:* 5
   - *2.6 Fluoride Oxalate Sample 6:* 5
   - *2.7 PAXgene DNA & RNA Vacutainers Samples 7-8:* 5
3. Urine Collection 5
   - *3.1 Required supplies* 5

1. Introduction

This biofluids collection protocol describes the process for collection of biofluids for the iBEAt study participants. Blood collection must be done by a qualified individual (phlebotomist, nurse, physician, research practitioner) in an appropriate clinical setting. When possible, sample collection should occur at the beginning of the study visit and participants provided with the standardized snack post-collection.

All blood and urine samples from a patient must be collected on the same day and the same visit. The patient must be fasted for a minimum of 8 hours. Thorough documentation of sample collection, handling, and processing must be collected on the source document and entered into the corresponding case report form (CRF).

2. Blood Collection

Obtain an iBEAt biofluids kit with corresponding participant study ID to the scheduled participant. Included in the kit will be collection *and* processing materials. Remove the 12 collection vacutainers for procuring the blood sample. Provide vacutainers to the qualified individual performing phlebotomy.

PLEASE NOTE: It is important to keep the collection materials with the corresponding processing materials as some of the tubes will have barcoded participant ID that must match the remaining processed bio-fluid samples.

Sites will be provided necessary adaptors if not using BD system. To avoid hemolysis of samples, it is suggested to use a minimum of 21g needle for blood procurement. Direct blood collection into study vacutainers is recommended as an additional step to avoid hemolysis.

2.1 Required Supplies:

Blood collection supplies included in the kit are listed in table 1. Please place the remaining processing supplies aside for later use.

Provided by site:

- Blood collection supplies: alcohol swabs, needle or butterfly, vacutainer receiver, tourniquet, tube racks, any other locally required supplies

- Post-collection materials: cotton ball and/or gauze, band-aid or Coban

2.2 Draw order

Note the time the blood draw begins on the Bio-fluids source document. Using your site’s local blood collection protocol, collect the following blood tubes in order:

Table 1: Overview of blood collection tubes and their draw order

Sample (draw order)	Tube Type and Size	Number of Tubes	Total Volume (mL)	Stopper colour	Purpose and Disposition
**1**	Sample for waste	1	3		On-site disposal
**2**	Serum Vacutainer - 10 mL (without gel)	2	20	**RED**	Omics: biorepository
**3**	Serum Vacutainer - 5 mL (with gel)	1	5	**YELLOW**	Minimal + extended datasets: biorepository
**4**	K2EDTA Plasma Vacutainer - 10 mL	2	20	**PURPLE**	Omics: biorepository
**5**	K2EDTA Plasma Vacutainer - 2x3 mL	2	6	**PURPLE**	Hct, HgB, HbA1C: Local Lab
**6**	Fluoride oxalate 2 mL Vacutainer	1	2	**GREY**	Glucose: Local Lab
**7**	PAXgene DNA Vacutainer - 8,5mL	1	8.5	**BLUE**	Omics: biorepository
**8**	PAXgene RNA Vacutainer - 2,5mL	2	5	**ORANGE**	Omics: biorepository
**Total**		**12**	**69.5**		

2.3 Waste Sample 1:

1. In a small vacutainer or syringe, withdraw 2-3 mL for waste prior to beginning blood draw for research samples.

2. Discard according to site practice.

2.4 Serum Samples 2 and 3:

1. Collect samples in 2 x 10 mL and 1 x 5 mL serum vacutainers.
2. Invert tubes gently 8-10 times.
3. Store at room temperature for a minimum of 30 minutes to a maximum of 60 minutes to allow clot to form.
4. If not processed immediately following clotting period, please refrigerate at 4°C for no longer than 4 hours until processed.

2.5 K2EDTA Plasma Samples 4 and 5:

1. Collect samples in 2 x 10 mL and 2 x 3 mL K2EDTA plasma vacutainers.
2. Invert tubes gently 8-10 times.
3. Sample 4 should be stored at room temperature for 30 minutes or at 4 degrees C (refrigerated) for no longer than 4 hours prior to processing.
4. Sample 5 should be labeled with patient label and directed to local lab for HgB, Hct, and A1C measures.

2.6 Fluoride Oxalate Sample 6:

1. Collect sample in 1 x 2 mL Fluoride oxalate vacutainer.
2. Sample should be labeled with patient label and directed to local lab for glucose measure.

2.7 PAXgene DNA & RNA Vacutainers Samples 7-8:

1. Collect samples in each PAXgene vacutainer.
2. Invert tubes gently 8-10 times.
3. Store at room temperature for 2 hours.
4. No further processing is needed, freeze at -80°C.

3. Urine Collection

Consented participants should have been instructed to collect a first morning void upon waking, prior to their scheduled visit (please see PATIENT HANDOUT: Clean Catch Urine Collection Guidelines). Participants have been instructed to maintain the fresh sample(s) refrigerated if the study visit will be greater than 4 hours from the time of procurement.

Document the time of the first void (Void 1) in the Bio-fluids source document for transfer to the CRF.

An additional void sample (Void 2) should be collected on the study morning according to the same collection instructions, and the time should be documented.

Pour off 2-4 mL of the first morning void and submit to local lab for UACR reading.

3.1 Required supplies

Urine collection supplies included in kit are listed in table 2:

Table 2. Urine collection supplies

Sample	Sample Type	Label	Container Type and Volume	Number of Containers	Total Volume to be collected	Purpose and Disposition
SC[a]	Random	Local*	Urine 100 mL collection container from local supply**	1	20-30 mL	Local lab: UACR
BL[b]	1st morning void	Local*,#	Urine 100 mL collection container from local supply**	1	2-5 mL	Local lab: UACR
1	1st morning void	VOID 1 Container 1	Urine 100 mL collection container	1	75-90 mL	Exosomes + Protein Analyses: biorepository
2	Additional morning void	VOID 2 Container 2	Urine 100 mL collection container	1	75-90 mL	Omics: biorepository

[a] Screening visit
[b] Baseline visit
*For immediate submission to local laboratory with patient label for UACR measure.
**As this will be a locally processed clinical laboratory result, please obtain container from local supply to adhere to site policy for sample processing
[#] Should be poured off the VOID 1 (1st morning void sample).
