# Supplementary material for: Prognostic imaging biomarkers for diabetic kidney disease (iBEAt): study protocol
Source: BMC Nephrol. 2020 Jun 29;21:242. doi: 10.1186/s12882-020-01901-x (PMC7323369; doi:10.1186/s12882-020-01901-x)
Supplement: Supplementary file 2 — Additional file 2: 2.1 Biofluid collection SOPs. PDF file. Biofluid collection protocol. The protocol for the collection of blood and urine samples within iBEAt. 2.2 SOPs Biofluid processing. PDF file. Biofluid processing protocol. The protocol for processing blood and urine samples within iBEAt. 2.3 Biofluid schematics. PDF file. iBEAt kit contents and biofluid processing schematics. Schematics of iBEAt collection kits, and processing and storage protocols for collected blood and urine samples within iBEAt. [file 12882_2020_1901_MOESM2_ESM.zip › Additional file 2.2 biofluid processing SOPsR1.pdf]

Prognostic Imaging Biomarkers for Diabetic Kidney Disease (iBEAt)

Bio-fluids Processing Procedures

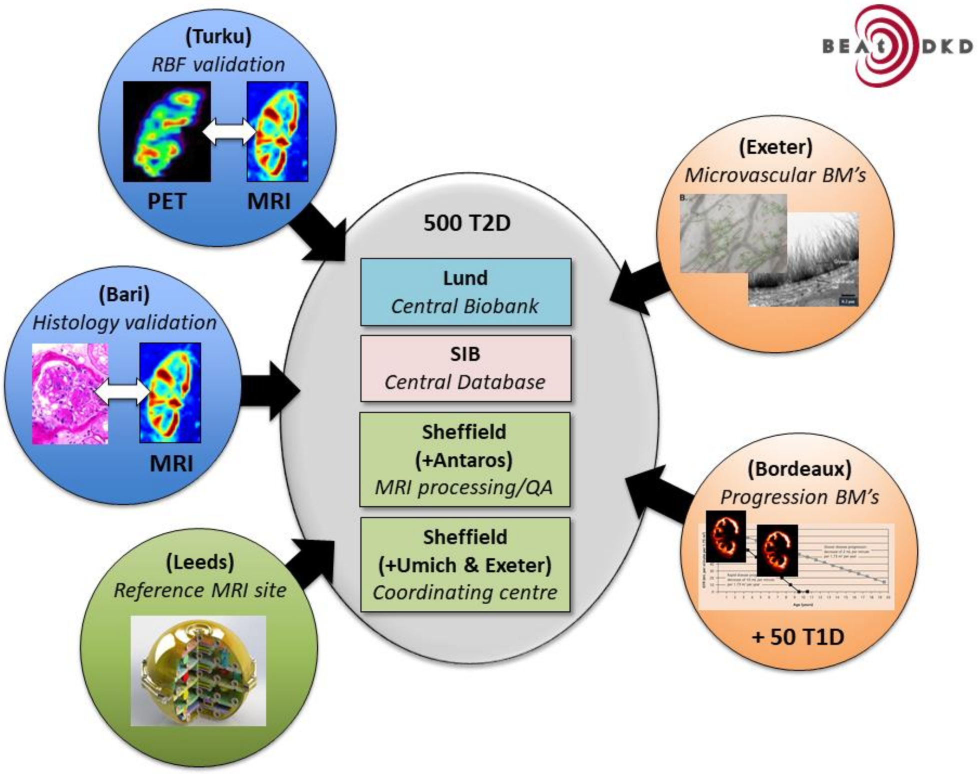

Version 2.0
03.06.2019

TABLE OF CONTENTS

Contents

1 Introduction 3

2 General sample processing guidelines 3

3 Required equipment and supplies 3

3.1 Provided by iBEAT central biorepository 3

3.1.1 Chemicals and reagents 3

3.1.2 Tubes for processing the collected blood samples 1-4 4

3.1.3 Tubes for processing the collected urine samples 4

3.2 Equipment and consumables to be provided by local site 5

4 Blood processing 6

4.1 *Processing of samples 2, 3 and 4* 6

4.1.1 Serum sample 2 7

4.1.2 Serum sample 3 7

4.1.3 K2-EDTA plasma sample 4 7

4.2 *Samples 5 & 6: Local laboratory processing* 8

4.3 *PAXgene DNA & RNA* 8

5 Urine processing 8

5.1 *Void 1 sample processing* 9

5.1.1 Void 1 Container 1 9

5.2 *Void 2 sample processing* 9

5.2.1 Void 2 Container 2 10

1. Introduction

This protocol describes how to process the samples collected at the study visit. Blood processing must be done by a qualified individual (phlebotomist, laboratory technician, nurse, physician, etc) who has been trained and certified for iBEAt biofluid processing by the study manager, in an appropriate laboratory setting, with all local institutional biofluid handling guidelines.

Thorough documentation of sample collection, handling, and processing must be collected on the biofluid source document and entered into the corresponding case report form (CRF).

2. General sample processing guidelines

As indicated above, local institutional biofluid handling policies must be followed, in addition to the following guidelines:

- Universal precautions and Occupational Safety and Health Administration (OSHA) must be followed (please see www.osha.gov/SLTC/biologicalagents.index.html for further information)
- Gloves must be worn at all times, including during:
  - Removal of rubber stopper from blood tubes
  - Centrifugation
  - Pipetting and transferring of all biofluids
  - Handling biofluid procurement container
  - Spill clean-up
- Tubes, needles, and pipettes must be properly disposed in appropriate biohazard containers
- Please carefully review the MSDS documentation included with the kits

3. Required equipment and supplies

3.1 Provided by iBEAt Central Biorepository Lab

3.1.1 Chemicals & Reagents

- Protease Inhibitor tablets to be cut in halves locally
- Butylated hydroxytoluene (BHT) powder in 15 mL Falcon tube to be diluted in absolute ethanol and stored in 20 µL aliquots in 100 Eppendorf tubes as 1000x BHT stock in -20˚C freezer (labelled with red dot)
- PBS pouch to be prepared and stored in 200 µL aliquots in 100 Eppendorf tubes (labelled with

blue dot)

- RNA-Later[TM] stored in 3 aliquots of 10 mL in 15 mL Falcon tubes
- Citrate-EDTA buffer 300 ml aliquoted in 50 ml Falcon tubes

3.1.2 Tubes for processing the collected blood samples 1-4 (iBEAt kit contents and biofliud processing schematics supplement):

- For sample 2 (S2):
  - Set 1a+: 4 x 0.5 mL cryovials: RED CAPS
  - Set 1b+: 4 x 1.0 mL cryovials: RED CAPS
  - Set 2a: 4 x 0.5 mL cryovials: GREEN CAPS
  - Set 2b: 4 x 1.0 mL cryovials: GREEN CAPS
- For sample 3 (S3):
  - 4 x 0.5 mL cryovials: YELLOW CAPS
- For sample 4 (P4):
  - Set 3a+: 4 x 0.5 mL cryovials: PURPLE CAPS
  - Set 3b+: 4 x 1.0 mL cryovials: PURPLE CAPS
  - Set 4a: 4 x 0.5 mL cryovials: BLUE CAPS
  - Set 4b: 4 x 1.0 mL cryovials: BLUE CAPS

3.1.3. Tubes for processing the collected urine samples (iBEAt kit contents and biofliud processing schematics supplement):

- For first morning void
  - Sample 1:
    - 2 x 0.5 mL cryovials labeled Void 1 Sample 1 (U V1S1): WHITE CAPS
  - Sample 2:
    - 1 x 50 mL processing Falcon tube (unlabelled*, for processing only)
    - 1 x 50 mL Falcon tube labelled Void 1 Sample 2 (U V1S2)

    - 3 x 1.0 mL cryovials labelled Void 1 Sample 2 (U V1S2): WHITE CAPS
  - Sample 3:
    - 1 x 0.5 mL cryovial labelled Void 1 Sample 3 (U V1S3 Pellet): WHITE CAP

- For second collected morning void
  - Sample 1:
    - 1 x 12 mL matrix tube labelled Void 2 Sample 1 (U V2S1)
    - 3 x 1.0 mL cryovials labelled Void 2 Sample 1 (U V2S1): CLEAR CAPS
  - Sample 2:
    - 2 x 50 mL processing Falcon tubes (unlabelled*, for processing only)
    - 1 x 15 mL Falcon tube labelled Void 2 Sample 2 (U V2S2)
    - 4 x 1.0 mL cryovials labelled Void 2 Sample 2 (U V2S2)
  - Sample 3:
    - 1 x 0.5 mL cryovial labelled Void 2 Sample 3 (U V2S3 Pellet): CLEAR CAP

*Please note that the 3 Falcon tubes for processing only have no barcode labels, but could be marked with a pen (e.g. U V1P for void 1 and U V2PA, U V2PB for void 2, as indicated in the iBEAt schematics) to avoid mixing void 1 and void 2 samples if processing in parallel.

3.2 Equipment and consumables to be provided by local site:

- Blood and urine samples from study participant
- Horizontal rotor (swing-out head) centrifuge capable of 3000 x G
- Aerosol spray covers for centrifuge buckets
- Pipettes and tips
- Gloves, goggles, and lab coat
- Biospecimen hood (if required by local policy)

4. Blood processing

The following table summarized all blood samples collected per patient and visit (please refer to the biofluid collection SOP supplement for more details):

Table 1: Overview of blood samples collection

Sample	Tube Type & Size	Number of tubes	Total Volume (mL)	Prior to processing
**1**	Local tube/syringe	1	3-5	*Discard if provided for processing*
**2**	Serum Vacutainer – 10 mL (without gel)	2	20	Allow sample to stand at room temperature for 30 minutes minimum; 60 minutes maximum
**3**	Serum Vacutainer – 5 mL (with gel)	1	5	Allow sample to stand at room temperature for 30 minutes minimum; 60 minutes maximum
**4**	K2-EDTA Plasma Vacutainer – 10 mL	2	20	Allow sample to stand at room temperature for 30 minutes minimum; 60 minutes maximum
**5**	K2-EDTA Plasma Vacutainer – 2x3 mL	2	6	Send immediately to local lab for Hct, Hgb, HbA1C analysis
**6**	Fluoride Oxalate – 2 mL	1	2	Send immediately to local lab for glucose analysis
**7**	PAXgene DNA Vacutainer – 8.5 mL	1	8,5	Allow sample to stand at room temperature for 2 hours prior to freezing
**8**	PAXgene RNA Vacutainer – 2x2.5 mL	2	5	Allow sample to stand at room temperature for 2 hours prior to freezing
	**TOTAL:**	**11-12**	**69.5**	

4.1 Processing of serum samples 2, 3 (S2, S3) and K2-EDTA plasma sample 4 (P4):

1. If samples cannot be processed within 60 minutes, please refrigerate at 4^0C for up to 4 hours until processed.
2. Following the 30-60 minute incubation period, balance samples 2, 3, and 4 per your site's centrifuge protocol and handling policy.

3. Centrifuge vacutainers at 2000 x G for 12 minutes at room temperature. Note the time on the biofluid source document (this should be the time the samples start to spin in the centrifuge).

4.1.1 Serum sample 2 (S2):

1. Remove cryovial caps and place them upside down on cleaned laboratory bench.
2. Remove rubber stopper from serum vacutainer.
3. Carefully collect the serum layer without disturbing the red blood cells below. This can be achieved by leaving a residual amount of serum atop the red blood cell layer.
4. Fill the serum cryovials by pipetting the serum into the following cryovials:
   a. Set 1a+: 500 µL into each of the 4 cryovials: RED CAPS
   b. Set 1b+: 900 µL into each of the 4 cryovials: RED CAPS
   c. Set 2a: 500 µL into each of the 4 cryovials: GREEN CAPS
   d. Set 2b: 900 µL into each of the 4 cryovials: GREEN CAPS
5. Add BHT for final concentration of 0.05% (vol/vol) to sets 1a+ and 1b+ (RED CAPS) as follows:
   a. Set 1a+: Add 5 µL of prepared 100x BHT solution
   b. Set 1b+: Add 9 µL of prepared 100x BHT solution
6. Re-cap and close tightly.
7. Store at -80^{0}C.

4.1.2 Serum sample 3 (S3):

1. Remove cryovial caps and place them upside down on cleaned laboratory bench.
2. Remove rubber stopper from serum vacutainer.
3. Carefully collect the serum layer without disturbing the red blood cells below, this can be achieved by leaving a residual amount of serum atop the red blood cell layer.
4. Fill the serum cryovials by pipetting the supernatant into the following cryovials:
   a. 500 µL in each of the 4 cryovials: YELLOW CAPS
5. Re-cap and close tightly.
6. Store at -80^{0}C.

4.1.3 K2-EDTA plasma sample 4 (P4):

1. Remove cryovial caps and place upside down on cleaned laboratory bench.

2. Remove rubber stopper from K2-EDTA vacutainers.

3. Carefully collect the plasma layer without disturbing the buffy coat layer (white layer atop the spun red blood cells). This can be achieved by leaving a residual amount of plasma atop the buffy coat layer.

4. Fill the K2-EDTA plasma cryovials by pipetting the supernatant into the following cryovials:

    a. Set 3a+: 500 µL in each of the 4 cryovials: PURPLE CAPS

    b. Set 3b+: 900 µL in each of the 4 cryovials: PURPLE CAPS

    c. Set 4a: 500 µL in each of the 4 cryovials: BLUE CAPS

    d. Set 4b: 900 µL in each of the 4 cryovials: BLUE CAPS

5. Add BHT for final concentration of 0.05% (vol/vol) to sets 3a+ and 3b+ (PURPLE CAPS) as follows:

    a. Set 3a: 5 µL of prepared BHT solution

    b. Set 3b: 9 µL of prepared BHT solution

6. Re-cap and close tightly.

7. Store at -80^{0}C.

4.2 Samples 5 & 6 (unlabeled): Local lab processing

1. Please label with local Patient Hospital ID and send to local lab for analysis of these whole blood samples.

2. Research nurse/coordinator should retrieve results from the patient medical record and enter on the corresponding CRF.

4.3 PAXgene DNA & RNA samples 7-8 (Blood for DNA/RNA)

1. PAXgene DNA & RNA tubes must remain at room temperature for a minimum of 2 hours (120 minutes).

2. Please document total time in minutes on the biofluid source document for transfer into the CRF.

3. After incubation period store at -80^{0}C.

5. Urine processing

Samples can be stored at 4°C (refrigerated or kept on ice as needed) for up to a maximum of 6 hours prior to processing. Local procedures should be implemented for UACR samples collected at Screening / Consent and the Baseline UACR specimen.

5.1 Void 1 sample processing

Gather the Void 1 sample processing supplies listed in Section 3.1.2 as well as the Void 1 Container 1 with the urine specimen. 75-90 mL urine have been requested, if less than 75 mL is provided, priority should go to the Void 1 Sample 2 (U V1S2).

5.1.1 Void 1 Container 1:

1. Gently invert Void 1 Container 1 8-10 times to re-suspend any sediment that may have settled.
2. Transfer urine from Void 1 Container 1 into the following tube/cryovials:
    a. U V1S1: 500 µL whole urine into each of the 2 cryovials
    b. "U V1P": 50 mL whole urine into 50 mL processing Falcon tube. This tube is for processing only and hence, do not have a bar coded label. Please mark it "U V1P" to prevent confusion with void 2 samples.
    c. Add 1⁄2 Protease Inhibitor tablet to "U V1P"
3. Balance sample per your site's centrifuge protocol and handling policy.
4. Note the time on the biofluid source document (this should be the time the sample starts to spin in the centrifuge).
5. Centrifuge "U V1P" at 3000 x G for 10 minutes at room temperature without brake.
6. Remove the sample from the centrifuge taking care not to disturb the separated contents.
7. Transfer supernatant from "U V1P" urine to the following tubes/cryovials:
    a. U V1S2: 900 µL into each of the 3 cryovials
    b. U V1S2: 45 mL into 50 mL Falcon tube
    c. Add 2.5 mL citrate buffer to the 50 mL tube containing U V1S2
8. Drain any remaining residual urine supernatant from "U V1P" to isolate the urine pellet.
    a. Resuspend the pellet by adding 100-300 µL RNA-LaterTM depending on the size of the pellet
    b. Transfer the resuspended pellet to 1 x 0.5 mL cryovial labelled U V1S3
9. Store all labelled samples at -80°C.

5.2 Void 2 sample processing

Gather the Void 2 sample processing supplies listed in Section 3.1.2 as well as the Void 2 Container 2 with the urine specimen.

5.2.1 Void 2 Container 2:

1. Gently invert Void 2 Container 2 8-10 times to resuspend any sediment that may have settled.
2. Transfer urine from Void 2 Container 2 urine into the following tube:
    a. "U V2P A": 50 mL whole urine into 50 mL processing Falcon tube. This tube is for processing only and hence, do not have a bar coded label. Please mark it "U V2P A" to prevent confusion with void 1 sample.
3. Balance sample per your site's centrifuge protocol and handling policy.
4. Note the time on the biofluid source document (this should be the time the sample starts to spin in the centrifuge).
5. Centrifuge "U V2P A" at 3000 x G for 10 minutes at room temperature without brake.
6. Transfer supernatant from "U V2P A" into the following tubes:
    a. U V2S1: 9 mL into the 12 mL tube
    b. U V2S1: 900 µL into each of the 3 cryovials
    c. "U V2P B": 38 ml into the second 50 mL processing Falcon tube. This tube is also for processing only and hence, do not have a bar coded label. Please mark it "U V2P B" to prevent confusion.
7. Add 1⁄2 Protease Inhibitor tablet to "U V2P B", gently mix and aliquot into the following tubes/cryovials:
    a. U V2S2: 13 ml into a 15 mL Falcon tube
    b. U V2S2: 900 µL into each of the 4 cryovials
8. Drain any remaining residual urine supernatant from "U V2P A" to isolate the urine pellet.
    a. Resuspend the pellet by adding 100-300 mL RNA-LaterTM depending on the size of the pellet
    b. Transfer the resuspended pellet to 1 x 0.5 mL cryovial labelled U V2S3
9. Store all labelled samples at -80^{0}C.
