# Supplementary material for: Prognostic imaging biomarkers for diabetic kidney disease (iBEAt): study protocol
Source: BMC Nephrol. 2020 Jun 29;21:242. doi: 10.1186/s12882-020-01901-x (PMC7323369; doi:10.1186/s12882-020-01901-x)
Supplement: Supplementary file 3 — Additional file 3: 3.0 CRF Screening. PDF file. Study recruitment – prescreening / screening. Clinical record form for prescreening / screening data. 3.1 CRF Adherence Checklist. PDF file. Baseline visit (V1) – adherence checklist. Clinical record form documenting participant adherence to guidance for the baseline visit. 3.2 CRF Limited Clinical Exam. PDF file. Limited Clinical Exam. Clinical record form for clinical examination data including, for example, blood pressure, height and weight. 3.3 CRF Medical and Family Hx. PDF file. Baseline (V1) – Medical and family history V2. Clinical record form for medical and family history (version 2). 3.4 CRF Local Study Labs. PDF file. Baseline (V1) – local study labs. Clinical record form for laboratory measurements performed at recruiting centre. 3.5 CRF Routine Labs. PDF file. Baseline visit (V1) – labs. Clinical record form for documenting all available laboratory values in the year prior to the baseline visit. 3.6 CRF Medications. PDF file. Medication log. Clinical record form documenting all current medications. 3.7 CRF Ultrasound. PDF file. Baseline visit (V1) – Ultrasound. Clinical record form for the renal ultrasound measurements. 3.8 CRF Biosamples. PDF file. Study biosamples. Clinical record form / checklist documenting what biofluid samples were collected and processed for the iBEAt study. [file 12882_2020_1901_MOESM3_ESM.zip › Additional file 3.0 CRF ScreeningR1.pdf]

Study Recruitment – Pre-Screening / Screening

Instructions: Potential participants are identified by a study team member and/or investigators' review of patients seen in clinic for primary diagnosis of kidney disease in the management of their diabetes. Patients in pre-defined registries are eligible as well. Please use the following checklist to determine initial eligibility.

Patient Initials:_________ Pre-screening date:_______________ Screening contact date: _______________

Gender: M / F Age: __________ Race:____

(Options are: White, etc

**INCLUSION**			
**Q**		**Response**	**Patient Confirmation?**
1	Does patient have Diabetes Type 2?	O Y O N	O Y O N
2	Is the patient's eGFR ≥ 30 ml/min/1.73m^2?	O Y O N	O Y O N
3	Is the patient's age between 18-80?	O Y O N	O Y O N
4	Has there been a change to their anti-diabetic/anti-hypertensive medications in the past 3 months?	O Y O N	O Y O N
5	Is the patient able to provide informed consent?	O Y O N	O Y O N
6	**Does potential participant meet ALL inclusion criteria?** If yes, please proceed to exclusion criteria.	O Y O N	
**EXCLUSION**			
7	Has patient had a transplant? *(corneal permitted)*	O Y O N	O Y O N
8	Is the patient on permanent dialysis?	O Y O N	O Y O N
9	Does the patient have significant co-morbidities with <1 year life expectancy?	O Y O N	O Y O N
10	Has the patient used any investigational drugs within the past 1 month?	O Y O N	O Y O N
11	Is the patient currently pregnant?	O Y O N	O Y O N
12	Does the patient have a positive history for Hepatitis B or Hepatitis C?	O Y O N	O Y O N
13	Does the patient have a history of antiretroviral medication usage?	O Y O N	O Y O N
14	Does the patient have a history or current renal or urinary tract malignancy?	O Y O N	O Y O N
15	Does the patient have biopsy proven non-diabetic primary renal disease?	O Y O N	O Y O N
16	Does the patient have autosomal dominant Polycystic Kidney Disease?	O Y O N	O Y O N
17	Does the patient have renal stones causing chronic kidney disease?	O Y O N	O Y O N
18	Does the patient have liver disease, or non-cirrhotic liver disease where ALT >2x upper normal limit?	O Y O N	O Y O N
19	Does the patient have a current metastatic malignancy?	O Y O N	O Y O N
20	Does the patient have a current malignancy with expected survival < study follow-up of 4 years?	O Y O N	O Y O N

21	Has the patient had melanomatous skin cancer < 5 years ago?	O Y O N	O Y O N
22	Does the patient have a known history of urinary obstruction on renal US of post-void residual > 100 mL or pyelactasis/hydronephrosis?	O Y O N	O Y O N
23	Does the patient have a known aortic endoprosthesis at the renal level?	O Y O N	O Y O N
24	Does the patient have a current or known history of renal or urinary tract malignancy?	O Y O N	O Y O N
25	Are there any other significant diseases or disorders which, in the opinion of the investigators, may either put the patient at risk because of study participation, or may influence the results of the study, or the patient's ability to participate in the study?	O Y O N	O Y O N
**STANDARD MRI EXCLUSIONS**			
26	Does the patient's weight exceed 250 kg?	O Y O N	O Y O N
27	Does the patient have a cochlear implant?	O Y O N	O Y O N
28	Does the patient have aneurysm clips?	O Y O N	O Y O N
29	Does the patient have a neurological stimulator?	O Y O N	O Y O N
30	Does the patient have implanted cardiac devices?	O Y O N	O Y O N
31	Does the patient have a metal heart valve?	O Y O N	O Y O N
32	Does the patient have a history of metal foreign orbital bodies?	O Y O N	O Y O N
33	Does the patient have other implanted metal devices?	O Y O N	O Y O N
34	Does the patient have a known allergy to Gadolinium contrast?	O Y O N	O Y O N
35	Does the patient have or report claustrophobia?	O Y O N	O Y O N
36	**Are ALL exclusion criteria negative?**<br>If yes, please approach the patient for study participation.	O Y O N	

Instructions: If all inclusion criteria are met, and all exclusion criteria are negative, please approach patient for study participation. After introducing the study, confirm with patient all criteria and proceed with the consent process, collect a random urine sample for local submission for UACR results, and schedule Baseline Visit (V1).

Consent obtained: Y / N Date consent obtained(dd/mm/yyyy):______________________

Contact details:

Patient Name: ______________________

Street Address: ______________________

City, Country, Code: ______________________

Contact phone number: ______________________
