# Supplementary material for: Prognostic imaging biomarkers for diabetic kidney disease (iBEAt): study protocol
Source: BMC Nephrol. 2020 Jun 29;21:242. doi: 10.1186/s12882-020-01901-x (PMC7323369; doi:10.1186/s12882-020-01901-x)
Supplement: Supplementary file 3 — Additional file 3: 3.0 CRF Screening. PDF file. Study recruitment – prescreening / screening. Clinical record form for prescreening / screening data. 3.1 CRF Adherence Checklist. PDF file. Baseline visit (V1) – adherence checklist. Clinical record form documenting participant adherence to guidance for the baseline visit. 3.2 CRF Limited Clinical Exam. PDF file. Limited Clinical Exam. Clinical record form for clinical examination data including, for example, blood pressure, height and weight. 3.3 CRF Medical and Family Hx. PDF file. Baseline (V1) – Medical and family history V2. Clinical record form for medical and family history (version 2). 3.4 CRF Local Study Labs. PDF file. Baseline (V1) – local study labs. Clinical record form for laboratory measurements performed at recruiting centre. 3.5 CRF Routine Labs. PDF file. Baseline visit (V1) – labs. Clinical record form for documenting all available laboratory values in the year prior to the baseline visit. 3.6 CRF Medications. PDF file. Medication log. Clinical record form documenting all current medications. 3.7 CRF Ultrasound. PDF file. Baseline visit (V1) – Ultrasound. Clinical record form for the renal ultrasound measurements. 3.8 CRF Biosamples. PDF file. Study biosamples. Clinical record form / checklist documenting what biofluid samples were collected and processed for the iBEAt study. [file 12882_2020_1901_MOESM3_ESM.zip › Additional file 3.1 CRF Adherence ChecklistR1.pdf]

*Baseline Visit (V1) – Adherence Checklist*

**Study ID:__________________**
**Study visit date:__________________**

**Instructions:** Please review the following adherence instructions for the baseline visit. NOT meeting all elements should not impede the study visit – but rather that all elements are documented. If the blood glucose measure does not meet the standard, the visit must be rescheduled.

| **ADHERENCE CHECKLIST** | | | |
|---|---|---|---|
| **Q** | | **Value** | **Unit** |
| 1 | Does the participant report any hypoglycaemic events prior to start of study visit? | **O** Y **O** N | |
| 2 | Has participant brought a first morning void to the study visit? | **O** Y **O** N | |
| 3 | Study Visit start time | | **HH:MM (24h format)** |
| 4 | Has participant fasted for at least 8 hours prior to study visit? | **O** Y **O** N | |
| 5 | Time since last meal: | | **HH:MM (24h format)** |
| 6 | Time since last drink (excluding water): | | **HH:MM (24h format)** |
| 7 | Total time of fast: | | **HH:MM** |
| 8 | Has participant abstained from smoking for a minimum of 4 hours prior to the scheduled study visit? | **O** Y **O** N | |
| 9 | Time of last cigarette | | **HH:MM (24h format)** |
| 10 | Duration since last cigarette: | | **HH:MM** |
| 11 | Has the participant avoided strenuous / out of the ordinary exercise for the past 24 hours? | **O** Y **O** N | |
| 12 | Total time inactive: | | **HH:MM** |
| 13 | Have hypoglycaemic medications been modified for study purposes? | **O** Y **O** N | |
| 14 | Blood glucose measure (must be > 3.5 mmol/l) | | |
| 15 | Based on the morning of study visit blood glucose measure, should the visit proceed as scheduled? | **O** Y **O** N | |
