# Supplementary material for: Prognostic imaging biomarkers for diabetic kidney disease (iBEAt): study protocol
Source: BMC Nephrol. 2020 Jun 29;21:242. doi: 10.1186/s12882-020-01901-x (PMC7323369; doi:10.1186/s12882-020-01901-x)
Supplement: Supplementary file 3 — Additional file 3: 3.0 CRF Screening. PDF file. Study recruitment – prescreening / screening. Clinical record form for prescreening / screening data. 3.1 CRF Adherence Checklist. PDF file. Baseline visit (V1) – adherence checklist. Clinical record form documenting participant adherence to guidance for the baseline visit. 3.2 CRF Limited Clinical Exam. PDF file. Limited Clinical Exam. Clinical record form for clinical examination data including, for example, blood pressure, height and weight. 3.3 CRF Medical and Family Hx. PDF file. Baseline (V1) – Medical and family history V2. Clinical record form for medical and family history (version 2). 3.4 CRF Local Study Labs. PDF file. Baseline (V1) – local study labs. Clinical record form for laboratory measurements performed at recruiting centre. 3.5 CRF Routine Labs. PDF file. Baseline visit (V1) – labs. Clinical record form for documenting all available laboratory values in the year prior to the baseline visit. 3.6 CRF Medications. PDF file. Medication log. Clinical record form documenting all current medications. 3.7 CRF Ultrasound. PDF file. Baseline visit (V1) – Ultrasound. Clinical record form for the renal ultrasound measurements. 3.8 CRF Biosamples. PDF file. Study biosamples. Clinical record form / checklist documenting what biofluid samples were collected and processed for the iBEAt study. [file 12882_2020_1901_MOESM3_ESM.zip › Additional file 3.3 CRF Medical and Family HxR1.pdf]

Medical and Family History – V2
Continuous form (please use for all visits)

Study ID:_________________
Baseline visit date:_________________
Follow-up Y1 date:_________________
Follow-up Y2 date:_________________
Follow-up Y3 date:_________________

Instructions: Participants should respond to all questions. Shaded areas should be taken from the patient available medical chart data. For baseline, only the 2 columns under "Baseline" are applicable, for follow-up, please use the columns under each respective year.

**MEDICAL HISTORY**		**Baseline**		**Year 1 FU**		**Year 2 FU**		**Year 3 FU**	
**Q**		**Response**							
1	Date of diagnosis Type 2 Diabetes (year)		**YYYY**						
2	Does the participant take medical therapy for their diabetes?	**O** Y **O** N		**O** Y **O** N		**O** Y **O** N		**O** Y **O** N	
3	Date Type 2 Diabetes medical therapy initiated (year)		**YYYY**	**O** Y<br>**O** N<br>**O** No chg	**If yes, year:**	**O** Y<br>**O** N<br>**O** No chg	**If yes, year:**	**O** Y<br>**O** N<br>**O** No chg	**If yes, year:**
4	Does the participant have a diagnosis of hypertension?	**O** Y **O** N<br>**O** Unknown		**O** Y<br>**O** N<br>**O** No chg	**If yes, year:**	**O** Y<br>**O** N<br>**O** No chg	**If yes, year:**	**O** Y<br>**O** N<br>**O** No chg	**If yes, year:**
5	Date of diagnosis for hypertension (year)		**YYYY**	**O** Y<br>**O** N<br>**O** No chg	**If yes, year:**	**O** Y<br>**O** N<br>**O** No chg	**If yes, year:**	**O** Y<br>**O** N<br>**O** No chg	**If yes, year:**
6	Does the participant take hypertensive medication?	**O** Y **O** N		**O** Y<br>**O** N<br>**O** No chg	**If yes, year:**	**O** Y<br>**O** N<br>**O** No chg	**If yes, year:**	**O** Y<br>**O** N<br>**O** No chg	**If yes, year:**
7	Date hypertensive medical therapy initiated (year)		**YYYY**	**O** Y<br>**O** N<br>**O** No chg	**If yes, year:**	**O** Y<br>**O** N<br>**O** No chg	**If yes, year:**	**O** Y<br>**O** N<br>**O** No chg	**If yes, year:**
8	Does the patient have diabetic retinopathy? **From available medical chart data (If no DR diagnosis is >14 months then record as unknown)**	**O** Y **O** N<br>**O** Unknown		**O** Y **O** N<br>**O** Unknown		**O** Y **O** N<br>**O** Unknown		**O** Y **O** N<br>**O** Unknown	

		Baseline	Year 1 FU	Year 2 FU	Year 3 FU
9	Grade of diabetic retinopathy: **From available medical chart data (If DR grade is >14 months record as unknown unless they have documented previous treatment for proliferative DR)**	**1** No DR<br>**2** Non-proliferative DR (including background/ microaneurysms only)<br>**3** Proliferative DR<br>**4** Previous Treatment for proliferative DR<br>**5** Unknown	**1**<br>**2**<br>**3**<br>**4**<br>**5**	**1**<br>**2**<br>**3**<br>**4**<br>**5**	**1**<br>**2**<br>**3**<br>**4**<br>**5**
10	Does the patient have maculopathy?<br>**From available medical chart data**<br>To be taken from clinical data if available. If data is > 14 months:<br>• Class no macular oedema as "Unknown"<br>• If "Documented macular oedema" (DMO) or previous treatment for DMO then class as 2 and 3, respectively	**1** No macular edema<br>**2** Documented macular edema<br>**3** Previous treatment for macular edema<br>**4** Unknown	**1**<br>**2**<br>**3**<br>**4**	**1**<br>**2**<br>**3**<br>**4**	**1**<br>**2**<br>**3**<br>**4**
11	Does the patient have diabetic neuropathy?<br>**From available medical chart data**<br>To be defined on monofilaments if data available. In patients with no neuropathy and no available data within last 14 months, document as "Unknown"	**O** Y **O** N<br>**O** Unknown	**O** Y **O** N<br>**O** Unknown	**O** Y **O** N<br>**O** Unknown	**O** Y **O** N<br>**O** Unknown
12	If yes, is the diabetic neuropathy painful?	**O** Y **O** N **O Not applicable**	**O** Y **O** N<br>**O Not applicable**	**O** Y **O** N<br>**O Not applicable**	**O** Y **O** N<br>**O Not applicable**
13	Does the patient have heart failure stage II or IV?	**O** Y **O** N<br>**O** Unknown	**O** Y **O** N<br>**O** Unknown	**O** Y **O** N<br>**O** Unknown	**O** Y **O** N<br>**O** Unknown

		Baseline		Year 1 FU		Year 2 FU		Year 3 FU	
14	Does the patient have any macrovascular diseases? (If yes then progress to questions 15, 16, & 17)	**O** Y **O** N<br>**O** Unknown		**O** Y<br>**O** N<br>**O** No chg	**If yes, year:**	**O** Y<br>**O** N<br>**O** No chg	**If yes, year:**	**O** Y<br>**O** N<br>**O** No chg	**If yes, year:**
15	Does the patient have history or evidence of coronary artery disease (check all that apply):	☐ Myocardial infarction<br>☐ Angina<br>☐ Angioplasty<br>☐ Stent<br>☐ CABG		☐ Myocardial infarction<br>☐ Angina<br>☐ Angioplasty<br>☐ Stent<br>☐ CABG		☐ Myocardial infarction<br>☐ Angina<br>☐ Angioplasty<br>☐ Stent<br>☐ CABG		☐ Myocardial infarction<br>☐ Angina<br>☐ Angioplasty<br>☐ Stent<br>☐ CABG	
16	If yes to history or evidence of coronary artery disease, is there evidence or history of peripheral artery disease?	**O** Y **O** N		**O** Y **O** N		**O** Y **O** N		**O** Y **O** N	
17	Does the patient have any history or evidence of cerebrovascular disease? (Ischemic stroke and TIA confirmed by specialist (excluding TIA not confirmed by specialist, hemorrhagic stroke, and stroke associated with blood disease, tumors, trauma or surgical procedures))	**O** Y **O** N<br>**O** Not applicable<br>**O** Unknown		**O** Y **O** N<br>**O** Not applicable<br>**O** Unknown		**O** Y **O** N<br>**O** Not applicable<br>**O** Unknown		**O** Y **O** N<br>**O** Not applicable<br>**O** Unknown	
18	Does the patient have a history of malignancy?	**O** Y **O** N **O** Unknown		**O** Y<br>**O** N<br>**O** No chg	**If yes, year:**	**O** Y<br>**O** N<br>**O** No chg	**If yes, year:**	**O** Y<br>**O** N<br>**O** No chg	**If yes, year:**
19	When? (year)		**YYYY**						

Study ID:___________________
Baseline visit date:___________________

	**SOCIAL HISTORY**	**Baseline**	**Year 1 FU**	**Year 2 FU**	**Year 3 FU**
20	Current or previous tobacco use? (check all that apply)	☐ Cigarettes<br>☐ Pipe<br>☐ Cigars, cigarillos, or little cigars<br>☐ E-cigs<br>☐ Smokeless tobacco<br>☐ Never used tobacco	☐ Cigarettes<br>☐ Pipe<br>☐ Cigars, cigarillos, or little cigars<br>☐ E-cigs<br>☐ Smokeless tobacco<br>☐ Never used tobacco	☐ Cigarettes<br>☐ Pipe<br>☐ Cigars, cigarillos, or little cigars<br>☐ E-cigs<br>☐ Smokeless tobacco<br>☐ Never used tobacco	☐ Cigarettes<br>☐ Pipe<br>☐ Cigars, cigarillos, or little cigars<br>☐ E-cigs<br>☐ Smokeless tobacco<br>☐ Never used tobacco
21	If current or previous smoker, number of cigarettes smoked a day:	**O** Not applicable	**O** Not applicable	**O** Not applicable	**O** Not applicable
22	If current or previous smoker, number of years smoking:	**O** Not applicable	**O** Not applicable	**O** Not applicable	**O** Not applicable
23	If previous tobacco user, number of years tobacco-free:	**O** Not applicable	**O** Not applicable	**O** Not applicable	**O** Not applicable
	**FAMILY HISTORY**				
24	Does patient have a family history of renal disease?	**O** Y **O** N<br>**O** Unknown	**O** Y **O** N<br>**O** Unknown	**O** Y **O** N<br>**O** Unknown	**O** Y **O** N<br>**O** Unknown
25	Does patient have a family history of hypertension?	**O** Y **O** N<br>**O** Unknown	**O** Y **O** N<br>**O** Unknown	**O** Y **O** N<br>**O** Unknown	**O** Y **O** N<br>**O** Unknown
26	Does patient have a family history of Type 2 diabetes?	**O** Y **O** N<br>**O** Unknown	**O** Y **O** N<br>**O** Unknown	**O** Y **O** N<br>**O** Unknown	**O** Y **O** N<br>**O** Unknown
27	Does patient have a family history of cardiovascular disease?	**O** Y **O** N<br>**O** Unknown	**O** Y **O** N<br>**O** Unknown	**O** Y **O** N<br>**O** Unknown	**O** Y **O** N<br>**O** Unknown
28	Does patient have a family history of malignancy?	**O** Y **O** N<br>**O** Unknown	**O** Y **O** N<br>**O** Unknown	**O** Y **O** N<br>**O** Unknown	**O** Y **O** N<br>**O** Unknown
29	Does patient have a family history of Type I Diabetes?	**O** Y **O** N<br>**O** Unknown	**O** Y **O** N<br>**O** Unknown	**O** Y **O** N<br>**O** Unknown	**O** Y **O** N<br>**O** Unknown
