# Supplementary material for: Prognostic imaging biomarkers for diabetic kidney disease (iBEAt): study protocol
Source: BMC Nephrol. 2020 Jun 29;21:242. doi: 10.1186/s12882-020-01901-x (PMC7323369; doi:10.1186/s12882-020-01901-x)
Supplement: Supplementary file 3 — Additional file 3: 3.0 CRF Screening. PDF file. Study recruitment – prescreening / screening. Clinical record form for prescreening / screening data. 3.1 CRF Adherence Checklist. PDF file. Baseline visit (V1) – adherence checklist. Clinical record form documenting participant adherence to guidance for the baseline visit. 3.2 CRF Limited Clinical Exam. PDF file. Limited Clinical Exam. Clinical record form for clinical examination data including, for example, blood pressure, height and weight. 3.3 CRF Medical and Family Hx. PDF file. Baseline (V1) – Medical and family history V2. Clinical record form for medical and family history (version 2). 3.4 CRF Local Study Labs. PDF file. Baseline (V1) – local study labs. Clinical record form for laboratory measurements performed at recruiting centre. 3.5 CRF Routine Labs. PDF file. Baseline visit (V1) – labs. Clinical record form for documenting all available laboratory values in the year prior to the baseline visit. 3.6 CRF Medications. PDF file. Medication log. Clinical record form documenting all current medications. 3.7 CRF Ultrasound. PDF file. Baseline visit (V1) – Ultrasound. Clinical record form for the renal ultrasound measurements. 3.8 CRF Biosamples. PDF file. Study biosamples. Clinical record form / checklist documenting what biofluid samples were collected and processed for the iBEAt study. [file 12882_2020_1901_MOESM3_ESM.zip › Additional file 3.6 CRF MedicationsR1.pdf]

Medication Log

Study ID:__________________
Study visit date:__________________

Instructions: Please review the patient clinical chart and record all available current medications and review with the patient in-person to be sure they are currently using. Please enter this data into the REDCap database.

MEDICATION LOG							
**Q**	**Medication Drug Name**	**Medication Common Name**	**Strength & Units**	**Dose & Units**	**Frequency**	**Start Date**	**Stop Date**
0	*Ex: Acetaminophen*	*Tylenol*	*200 mg*	*600 mg*	*BID*	*6/2017*	*Ongoing*
1							
2							
3							
4							
5							
6							
7							
8							
9							
10							
11							
12							
13							
14							
15							
16							
17							

Medication Log

Study ID:____________________
Study visit date:____________________
