# Supplementary material for: Prognostic imaging biomarkers for diabetic kidney disease (iBEAt): study protocol
Source: BMC Nephrol. 2020 Jun 29;21:242. doi: 10.1186/s12882-020-01901-x (PMC7323369; doi:10.1186/s12882-020-01901-x)
Supplement: Supplementary file 3 — Additional file 3: 3.0 CRF Screening. PDF file. Study recruitment – prescreening / screening. Clinical record form for prescreening / screening data. 3.1 CRF Adherence Checklist. PDF file. Baseline visit (V1) – adherence checklist. Clinical record form documenting participant adherence to guidance for the baseline visit. 3.2 CRF Limited Clinical Exam. PDF file. Limited Clinical Exam. Clinical record form for clinical examination data including, for example, blood pressure, height and weight. 3.3 CRF Medical and Family Hx. PDF file. Baseline (V1) – Medical and family history V2. Clinical record form for medical and family history (version 2). 3.4 CRF Local Study Labs. PDF file. Baseline (V1) – local study labs. Clinical record form for laboratory measurements performed at recruiting centre. 3.5 CRF Routine Labs. PDF file. Baseline visit (V1) – labs. Clinical record form for documenting all available laboratory values in the year prior to the baseline visit. 3.6 CRF Medications. PDF file. Medication log. Clinical record form documenting all current medications. 3.7 CRF Ultrasound. PDF file. Baseline visit (V1) – Ultrasound. Clinical record form for the renal ultrasound measurements. 3.8 CRF Biosamples. PDF file. Study biosamples. Clinical record form / checklist documenting what biofluid samples were collected and processed for the iBEAt study. [file 12882_2020_1901_MOESM3_ESM.zip › Additional file 3.8 CRF BiosamplesR1.pdf]

Study Biosamples

Instructions: Provided below are the sample procurement tables for study visits. Please note that SC (screening visit) and BL (baseline visit) are only collected at the respective time points.

Table 1. Urine Procurement

Sample	Sample Type	Label	Container Type and Volume	Number of Containers	Total Volume to be collected	Purpose and Disposition
SC[a]	Random	Local*	Urine 100 mL collection container from local supply**	1	20-30 mL	Local lab: UACR
BL[b]	1st morning void	Local*	Urine 100 mL collection container from local supply**	1	20-30 mL	Local lab: UACR
1	1st morning void	VOID 1 Container 1	Urine 100 mL collection container	1	75-90 mL	Exosomes + Protein Analyses: biorepository
2	2nd morning void	VOID 2 Container 2	Urine 100 mL collection container	1	75-90 mL	Omics: biorepository
				**Total**	150-180	

[a] Screening visit
[b] Baseline visit
*For immediate submission to local laboratory with patient label for UACR measure. Should be poured off the VOID 1 (1st morning void sample).
**As this will be a locally processed clinical laboratory result, please obtain container from local supply to adhere to site policy for sample processing

**Urine Procurement**			
**Q**		**Response**	
1	Did the participant bring a first morning void (FMV) to the study visit?	**O** Y **O** N	
2	If not on the date of the visit, please indicate the date of the FMV:	**O** Not applicable	
3	Volume of first morning void (within 10 mL):	**mL**	
4	Amount poured off first morning void for local submission for UACR:	**mL**	
5	Did the participant provide a second morning void?	**O** Y **O** N	
6	Volume of second morning void (within 10 mL):	**mL**	

Urine Processing – Void 1 Container 1		
7	Number of U V1S1 unspun urine cryovials (0,5 mL tubes):	
8	Number of U V1S2 50 mL Falcon tubes:	
9	Total volume U V1S2 sample:	**mL**
10	Number of U V1S2 1,0 mL cryovials:	
11	Number of U V1S3 pellet cryovials (0,5 mL tube):	
**Urine Processing – Void 2 Container 2**		
12	Number of U V2S1 unspun urine 12 mL tube:	
13	Total volume U V2S1 sample:	
14	Number of U V2S1 1,0 mL tubes:	
15	Number of U V2S2 spun urine 15 mL tube:	
16	Total volume U V2S2 sample:	
17	Number of U V2S2 1,0 mL tubes:	
18	Number of U V2S3 pellet cryovials (0,5 mL tube):	

☐ 19. Check this box if all urine samples procured and processed according to protocol

Table 2. Blood procurement

Sample	Tube Type and Size	Number of Tubes	Total Volume (mL)	Stopper	Purpose and Disposition
1	Sample for waste	1	3		On-site disposal
2	Serum Vacutainer - 10 mL (without gel)	2	20	**RED**	Omics: biorepository
3	Serum Vacutainer - 5 mL (with gel)	1	5	YELLOW	Minimal + extended datasets: biorepository
4	K2EDTA Plasma Vacutainer - 10 mL	2	20	**PURPLE**	Omics: biorepository
5	K2EDTA Plasma Vacutainer - 2x3 mL	2	6	**PURPLE**	Hct, HgB, A1C: **Local Lab**
6	Fluoride oxalate 2 mL Vacutainer	1	2	**GREY**	Glucose: **Local Lab**
7	PAXgene DNA Vacutainer - 8,5mL	1	8,5	**BLUE**	Omics: biorepository
8	PAXgene RNA Vacutainer - 2,5mL	2	5	**ORANGE**	Omics: biorepository
	**TOTAL**	**12**	**69,5**		

Study Biosamples

**Blood Procurement**		
20	Was blood procured on the date of the visit?	**O** Y **O** N
21	If not on the date of the visit, please indicate the date of blood draw:	**O** Not applicable
22	Was a waste tube drawn first?	**O** Y **O** N
23	Were 2 – 10 mL serum (without gel) tubes procured?	**O** Y **O** N
24	Total estimated volume:	**mL**
25	Was 1 – 5 mL serum (with gel) tube procured?	**O** Y **O** N
26	Total estimated volume:	**mL**
27	Were 2 – 10 mL K2EDTA plasma tubes procured?	**O** Y **O** N
28	Total estimated volume:	**mL**
29	Were 2 – 3 mL K2EDTA plasma tubes procured?	**O** Y **O** N
30	Were both tubes sent for local labs for Hct, HgB, A1C?	**O** Y **O** N
31	Was 1 – 2 mL fluoride oxalate tube procured?	**O** Y **O** N
32	Was the tube sent for local lab glucose measures?	**O** Y **O** N
33	Was 1 – 8.5 mL PAXgene DNA tube procured?	**O** Y **O** N
34	Total estimated volume:	**mL**
35	Were 2 – 2.5 mL PAXgene RNA tubes procured?	**O** Y **O** N
36	Total estimated volume:	**mL**
37	Was the waste tube disposed of properly?	**O** Y **O** N
**Blood Processing: S2 (10 mL serum tubes x 2)**		
38	Number of 4 x 500 uL serum S2 1a+ cryovials:	
39	Were 5 uL BHT added to each of these cryovials?	
40	Number of 4 x 900 uL serum S2 1b+ cryovials:	
41	Were 9 uL BHT added to each of these cryovials?	
42	Number of 4 x 500 uL serum S2 2a cryovials:	
43	Number of 4 x 900 uL serum S2 2b cryovials:	
**Blood Processing: S3 (5 mL serum tube with gel)**		
44	Number of 4 x 500 uL serum S3 cryovials:	
**Blood Processing: S4 (10 mL K2EDTA tubes x 2)**		
45	Number of 4 x 500 uL serum P4 3a+ cryovials:	
46	Were 5 uL BHT added to each of these cryovials?	
47	Number of 4 x 900 uL serum P4 3b+ cryovials:	
48	Were 9 uL BHT added to each of these cryovials?	
49	Number of 4 x 500 uL plasma P4 4a cryovials:	
50	Number of 4 x 900 uL plasma P4 4b cryovials:	
**No Processing: (PAXgene tubes x 3)**		
51	Total time (in minutes) DNA tube at room temperature:	
52	Total time (in minutes) RNA tubes x 2 at room temperature:	

☐ 53. Check this box if all blood samples procured and processed according to protocol
